# Oral nonpeptide GLP-1 receptor agonist VCT220 for obesity treatment: a randomized, double-blind, phase II trial

**DOI:** 10.1038/s41392-026-02859-2

**Published:** 2026-08-14

**Authors:** Linong Ji, Leili Gao, Ben Li, Mei Liu, Xianghua Zhang, Chao Meng

**Affiliations:** 1 Department of Endocrinology, Peking University People’s Hospital, Beijing, China; 2 Chengdu Vincentage Pharma Co. Ltd., Chengdu, China; 3 Department of General Medicine, Yueyang Central Hospital, Yueyang, Hunan China; 4 Department of General Medicine, Central Hospital Affiliated to Shandong First Medical University, Jinan, China

**Keywords:** Endocrinology, Biotechnology

## Abstract

VCT220 is a novel nonpeptide, orally available glucagon-like peptide-1 receptor agonist (GLP-1RA) designed to reduce the complex administration of injectable GLP-1RAs and the limited bioavailability of on-marketing oral agents for obesity care. We conducted a multicenter, randomized, double-blind, placebo-controlled, phase II trial (Registration no. CTR20233978 and NCT06569355) across 13 sites in China. Adults aged 18–75 years with overweight (BMI 24-28 kg/m² plus ≥ 1 comorbidity) or obesity (BMI ≥ 28 kg/m²) were randomly assigned (3:1) to receive once-daily VCT220 (80 mg, 120 mg, or 160 mg with slow or fast titration) or placebo for 16 weeks, alongside lifestyle counselling. The primary endpoint was the percentage change in body weight from baseline to week 16. Two-hundred and fifty participants were randomized (61 to placebo, 189 to VCT220). At week 16, mean body weight decreased by −5.75% (80 mg) to −9.73% (160 mg-FT) versus −1.61% with placebo (all *p* < 0.001). The proportion achieving ≥5% weight loss ranged from 55.4% (80 mg) to 90.3% (160 mg-ST) with VCT220 versus 13.1% with placebo. VCT220 also improved HbA1c, fasting insulin, and blood pressure. Adverse events were mainly mild-to-moderate gastrointestinal events, most common during titration, and rarely led to discontinuation. VCT220, a once-daily oral nonpeptide GLP-1RA, produced rapid and clinically meaningful weight loss with metabolic benefits over 16 weeks, showing a tolerability profile consistent with the class. These findings support further global phase III evaluation to further verify the efficacy and safety of VCT220 as well as potential cardiometabolic benefits.

## Introduction

Obesity and overweight are prevalent and growing global public health concerns that significantly increase the risk of long-term complications, including type 2 diabetes, cancer, hypertension, cardiovascular disease, and other serious health issues, compromising quality of life and life expectancy.^1^ The escalating prevalence of these conditions has placed an increasing burden on healthcare systems worldwide, necessitating urgent and effective public health interventions. Among the nations grappling with this epidemic, China stands out as having the highest absolute numbers of individuals affected by excess body weight. According to recent national data, the prevalence of overweight and obesity among Chinese adults has reached 34.3% and 16.4%, respectively.^2^ This rapid rise in obesity prevalence reflects the combined effects of sedentary lifestyles, dietary transitions toward high-calorie processed foods, and rapid urbanization over the past several decades. More concerning is the finding that obesity now ranks as the sixth leading risk factor for death and disability in China, posing a substantial challenge to the country’s healthcare system.^3^ Without effective and scalable interventions, the obesity epidemic is projected to worsen, further threatening public health and overwhelming healthcare resources in the coming decades.

In response to this growing burden of obesity and its associated complications, effective and sustainable pharmacological interventions have gained increasing prominence beyond comprehensive lifestyle management. Over the last decade, glucagon-like peptide-1 receptor agonists (GLP-1RAs) have revolutionized the therapeutic landscape of obesity and overweight, firmly establishing GLP-1RA-based pharmacotherapy as a cornerstone of body weight management.^4^ Several GLP-1RAs, including liraglutide, semaglutide, and the dual GLP-1/glucose-dependent insulinotropic polypeptide (GIP) receptor agonists, have been approved for obesity treatment by health authorities. Despite their clinical success, most GLP-1RAs require subcutaneous injections, which raise concerns about injection-site reactions and poor long-term adherence. While oral semaglutide has been approved for type 2 diabetes, its clinical application for weight loss in overweight and obesity is still under investigation in clinical trials.^5,6^ Although oral semaglutide represents a key milestone in expanding oral peptide-based therapy, its clinical use is limited by low bioavailability, strict administration requirements (e.g., fasting and specific timing relative to meals), and tolerability issues. These challenges can undermine patient compliance, highlighting the urgent need for alternative oral GLP-1RAs that bypass these barriers. Consequently, developing novel, nonpeptide GLP-1RAs with improved oral bioavailability and short titration algorithms could significantly enhance weight management outcomes and patient adherence.^7^

The small-molecule compound VCT220 was developed as a nonpeptide GLP-1RA for managing obesity and type 2 diabetes. As a once daily, orally administered agent, VCT220 eliminates the need for injections, thereby increasing the willingness to initiate treatment, enhancing the convenience of use, and improving long-term adherence. Unlike oral semaglutide, which requires absorption enhancers and strict fasting administration, VCT220 does not rely on specialized delivery technologies and can be taken with meals, further supporting sustained patient compliance.^7^ Phase I trials submitted to the Chinese health authority involving healthy participants (CTR20222374) and individuals with overweight, obesity (CTR20231482), or type 2 diabetes (CTR20230826) have shown favorable safety and tolerability profiles, comparable with those of established injectable GLP-1RAs. These initial findings laid the groundwork for the current phase II trial, which further evaluates the efficacy and safety of VCT220 in weight management.

To further rigorously investigate the optimized flexible titration regimen of oral GLP-1RA and body weight loss potential at high target maintenance dose, we conducted a randomized, double-blind, placebo-controlled, multicenter phase II trial across 13 clinical sites in China to assess the efficacy and safety of VCT220 in Chinese adults who had obesity or overweight with at least one weight-related comorbidity. All participants received once-daily oral VCT220 (80 mg, 120 mg, or 160 mg) or placebo, alongside standardized lifestyle counseling, over a 16-week treatment period, followed by a 2-week safety follow-up. Notably, a short dose escalation period (4–6 weeks) was designed in this study to fully leverage the flexible titration advantages of oral nonpeptide GLP‑1RA. This trial represents the first controlled evaluation of VCT220 in a Chinese population and addresses an urgent clinical need for effective and convenient oral anti-obesity therapies tailored to local demographics.

## Results

### Participant characteristics

**Fig. 1:**
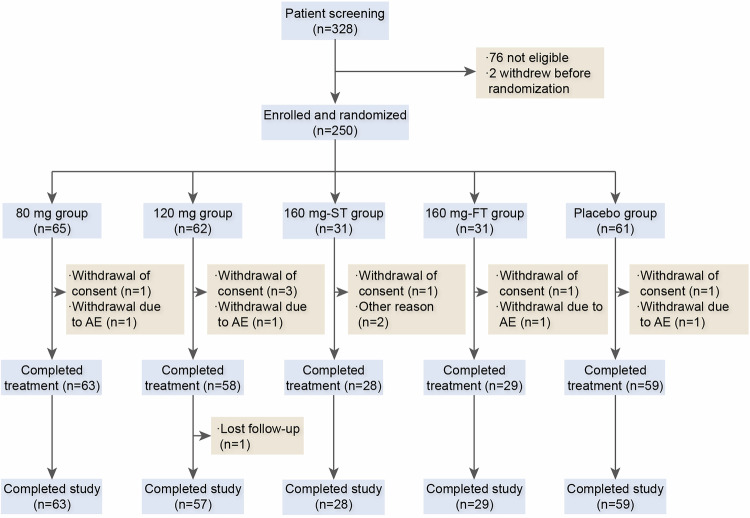
Flow diagram of the participants. The flow diagram illustrates the disposition of the participants screened and enrolled. ST slow titration, FT fast titration, AE adverse event

Between December 25, 2023 and March 22, 2024, 328 participants were screened, and 250 were randomized to receive VCT220 at doses of 80 mg, 120 mg, or 160 mg (62–65 participants per group, including 31 in each 160 mg titration subgroup) or a dose-matched placebo (*n* = 61) (Fig. 1). The baseline characteristics were well-balanced across the treatment groups (Table 1). The mean age of the participants was 32.4±7.46 years, with a mean body weight of 91.76±13.80 kg and a mean BMI of 32.03±3.27 kg/m^2^; 146 participants (58.4%) were male, and 104 (41.6%) were female. Among them, 237 participants (94.8%) completed the 16-week treatment, and 236 participants (94.4%) completed the study. All 250 participants met the inclusion criteria of both the full analysis set and the safety analysis set. Treatment was discontinued in 13 participants (5.2%). The most common reason for discontinuation was participant withdrawal (*n* = 7, 2.8%). Specific data on discontinuations due to adverse events (AEs), which occurred in 4 participants, is presented in Table 3.

**Table 1:** Demographic and clinical characteristics of the participants at baseline

	VCT220 80 mg (*N* = 65)	VCT220 120 mg (*N* = 62)	VCT220 160 mg-ST (*N* = 31)	VCT220 160 mg-FT (*N* = 31)	Placebo Pooled (*N* = 61)	Total (*N* = 250)
Age (years)	32.8 (7.06)	32.3 (7.27)	32.5 (7.02)	30.6 (6.78)	32.9 (8.63)	32.4 (7.46)
Sex [*n* (%)]						
Male	36 (55.4)	38 (61.3)	20 (64.5)	19 (61.3)	33 (54.1)	146 (58.4)
Female	29 (44.6)	24 (38.7)	11 (35.5)	12 (38.7)	28 (45.9)	104 (41.6)
Race [*n* (%)]						
Han	63 (96.9)	59 (95.2)	30 (96.8)	31 (100.0)	60 (98.4)	243 (97.2)
Others	2 (3.1)	3 (4.8)	1 (3.2)	0 (0.0)	1 (1.6)	7 (2.8)
Body weight (kg)	90.53 (13.09)	91.29 (14.62)	90.49 (11.49)	95.29 (14.73)	92.40 (14.37)	91.76 (13.80)
Height (m)	168.89 (9.49)	169.16 (9.19)	168.29 (8.78)	170.40 (8.66)	168.55 (9.02)	168.99 (9.06)
BMI (kg/m^2^)	31.68 (3.23)	31.77 (3.35)	31.91 (2.92)	32.71 (3.64)	32.39 (3.22)	32.03 (3.27)
BMI (kg/m^2^) [*n* (%)]						
≥24, < 28	4 (6.2)	5 (8.1)	1 (3.2)	3 (9.7)	3 (4.9)	16 (6.4)
≥28	61 (93.8)	57 (91.9)	30 (96.8)	28 (90.3)	58 (95.1)	234 (93.6)
Blood pressure (mmHg)						
SBP (mmHg)	122.5 (13.58)	124.0 (10.40)	123.1 (12.26)	125.7 (9.21)	124.7 (11.05)	123.9 (11.52)
DBP (mmHg)	82.6 (8.10)	80.9 (8.03)	82.3 (8.68)	83.7 (6.49)	82.5 (7.76)	82.3 (7.88)
Comorbidities at Screening [n (%)]						
Fatty Liver	55 (84.6)	52 (83.9)	28 (90.3)	25 (80.6)	58 (95.1)	218 (87.2)
Dyslipidemia	45 (69.2)	40 (64.5)	22 (71.0)	23 (74.2)	48 (78.7)	178 (71.2)
Prediabetes	17 (26.2)	17 (27.4)	1 (3.2)	6 (19.4)	10 (16.4)	51 (20.4)
Hypertension	13 (20.0)	9 (14.5)	8 (25.8)	4 (12.9)	12 (19.7)	46 (18.4)
Obstructive Sleep Apnea	1 (1.5)	0 (0.0)	0 (0.0)	0 (0.0)	1 (1.6)	2 (0.8)

Data presented are mean (SD) and *n* (%). *BMI* body mass index, *SBP* systolic blood pressure, *DBP* diastolic blood pressure, *SD* standard deviation

### Weight-related outcomes

**Fig. 2:**
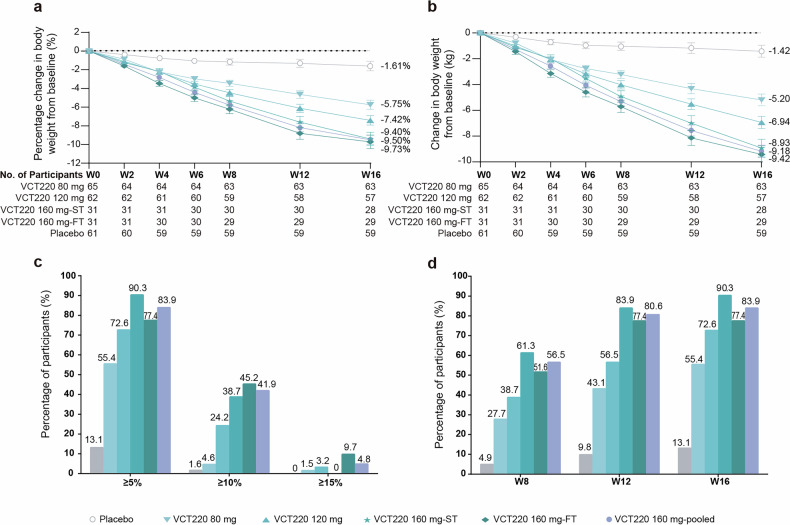
Comparison of body weight parameters with VCT220 versus placebo during the treatment period. **a** Observed mean percentage change in body weight over time. Numbers below the panels are the number of participants contributing to the mean. **b** Observed mean change in body weight (kg) over time. Numbers below the panels are the number of participants contributing to the mean. **c** Observed proportions of participants who had body weight reductions of at least 5%, 10%, and 15% at week 16. **d** Observed proportions of participants who had body weight reductions of at least 5% at week 8, week 12, and week 16. All data were analyzed using FAS with multiple imputation. Changes from baseline data are reported as the least squares mean ± SE. ST slow titration, FT fast titration, SE standard error, FAS full analysis set

VCT220 reduced weight in a dose-dependent manner and no apparent plateau was observed until week 16 (Fig. 2a). At week 16, the mean percentage weight reductions from baseline were significantly greater with VCT220 at all doses than with the pooled placebo: –5.75% (95% CI: –6.71 to –4.80) for 80 mg, –7.42% (95% CI: –8.41 to –6.44) for 120 mg, –9.40% (95% CI: –10.79 to –8.02) for 160 mg slow titration (ST), –9.73% (95% CI: –11.16 to –8.29) for 160 mg fast titration (FT), versus –1.61% (95% CI: –2.59 to –0.63) for the pooled placebo. The estimated treatment differences ranged from –4.14% (95% CI: –5.51 to –2.77) for 80 mg to –8.11% (95% CI: –9.85 to –6.38) for 160 mg-FT (Table 2). The absolute weight reductions (kg) reflected these percentage reductions (Fig. 2b, Table 2). Sensitivity analyses confirmed consistency with the results of the primary analysis (supplementary Tables 2, 3). Subgroup analysis results of weight changes using Chinese BMI category and comorbidity status were comparable with those using U.S. benchmark criteria (supplementary Table 8–11).

**Table 2:** Primary and selected secondary endpoints

	VCT220 80 mg (N = 65)	VCT220 120 mg (*N* = 62)	VCT220 160 mg-ST (*N* = 31)	VCT220 160 mg-FT (*N* = 31)	VCT220 160 mg Pooled (*N* = 62)	Placebo Pooled (*N* = 61)
Primary endpoint						
Body weight change from baseline^*^ (%)	−5.75 (−6.71, −4.80)	−7.42 (−8.41, −6.44)	−9.40 (−10.79, −8.02)	−9.73 (−11.16, −8.29)	−9.50 (−10.48, −8.51)	−1.61 (−2.59, −0.63)
ETD versus placebo (95% CI)	−4.14 (−5.51, −2.77)	−5.81 (−7.20, −4.43)	−7.79 (−9.49, −6.10)	−8.11 (−9.85, −6.38)	−7.89 (−9.28, −6.50)	NA
Secondary endpoint						
Body weight change from baseline (kg)	−5.20 (−6.11, −4.29)	−6.94 (−7.88, −6.01)	−8.93 (−10.28, −7.58)	−9.42 (−10.75, −8.09)	−9.18 (−10.12, −8.23)	−1.42 (−2.35, −0.49)
ETD versus placebo (95% CI)	−3.78 (−5.08, −2.48)	−5.53 (−6.84, −4.21)	−7.51 (−9.15, −5.87)	−8.00 (−9.62, −6.38)	−7.76 (−9.08, −6.44)	NA
Proportion of participants achieving body weight reduction ≥5%^†^, *n* (%)	36 (55.4)	45 (72.6)	28 (90.3)	24 (77.4)	52 (83.9)	8 (13.1)
ETD versus placebo^&^ (95% CI)	42.3 (26.17, 55.34)	59.5 (43.27, 70.91)	77.2 (58.61, 86.15)	64.3 (44.03, 77.15)	70.8 (55.36, 80.28)	NA
Proportion of participants achieving body weight reduction ≥10%^†^, *n* (%)	3 (4.6)	15 (24.2)	12 (38.7)	14 (45.2)	26 (41.9)	1 (1.6)
ETD versus placebo^&^ (95% CI)	3.0 (−4.73, 11.19)	22.6 (11.14, 34.59)	37.1 (20.50, 54.59)	43.5 (26.03, 60.64)	40.3 (26.83, 52.77)	NA
Proportion of participants achieving body weight reduction ≥15%^†^, *n* (%)	1 (1.5)	2 (3.2)	0 (0.0)	3 (9.7)	3 (4.8)	0 (0.0)
ETD versus placebo^&^ (95% CI)	1.5 (−4.52, 8.21)	3.2 (−3.14, 11.02)	NA	9.7 (1.01, 24.90)	4.8 (−1.88, 13.29)	NA
BMI change from baseline (kg/m^2^)	−1.80 (−2.11, −1.49)	−2.42 (−2.74, −2.10)	−3.13 (−3.59, −2.67)	−3.28 (−3.73, −2.83)	−3.21 (−3.53, −2.89)	−0.49 (−0.81, −0.18)
ETD versus placebo (95% CI)	−1.31 (−1.75, −0.86)	−1.93 (−2.37, −1.48)	−2.64 (−3.19, −2.08)	−2.79 (−3.34, −2.24)	−2.72 (−3.16, −2.27)	NA
Waist circumference change from baseline (cm)	−5.15 (−6.13, −4.17)	−6.48 (−7.48, −5.48)	−7.48 (−8.92, −6.04)	−7.73 (−9.15, −6.32)	−7.61 (−8.62, −6.60)	−1.99 (−2.98, −1.00)
ETD versus placebo (95% CI)	−3.16 (−4.56, −1.76)	−4.49 (−5.90, −3.08)	−5.49 (−7.24, −3.74)	−5.74 (−7.47, −4.01)	−5.62 (−7.03, −4.20)	NA
HbA1c change from baseline (%)	−0.11 (−0.17, −0.05)	−0.16 (−0.21, −0.10)	−0.19 (−0.27, −0.11)	−0.30 (−0.38, −0.21)	−0.24 (−0.30, −0.18)	0.06 (−0.00, 0.11)
ETD versus placebo (95% CI)	−0.17 (−0.25, −0.08)	−0.21 (−0.29, −0.13)	−0.25 (−0.35, −0.14)	−0.35 (−0.45, −0.25)	−0.30 (−0.38, −0.22)	NA
Fasting insulin change from baseline (mIU/L)	−2.13 (−4.65, 0.40)	−3.27 (−5.86, −0.68)	−4.18 (−7.90, −0.45)	−3.06 (−6.72, 0.61)	−3.61 (−6.22, −1.00)	3.22 (0.65, 5.79)
ETD versus placebo (95% CI)	−5.35 (−8.95, −1.74)	−6.49 (−10.15, −2.84)	−7.40 (−11.92, −2.87)	−6.28 (−10.76, −1.80)	−6.83 (-10.50, −3.16)	NA
Fasting plasma glucose change from baseline (mmol/L)	−0.17 (−0.27, −0.06)	−0.31 (−0.41, −0.20)	−0.30 (−0.45, −0.15)	−0.24 (−0.39, −0.10)	−0.27 (−0.38, −0.16)	−0.18 (−0.29, −0.08)
ETD versus placebo (95% CI)	0.02 (−0.13, 0.17)	−0.12 (−0.27, 0.03)	−0.11 (−0.30, 0.07)	−0.06 (−0.24, 0.12)	−0.09 (−0.23, 0.06)	NA
C-peptide change from baseline (μg/L)	0.09 (−0.13, 0.32)	0.03 (−0.20, 0.26)	−0.17 (−0.50, 0.16)	−0.12 (−0.45, 0.20)	−0.15 (−0.38, 0.09)	0.35 (0.12, 0.58)
ETD versus placebo (95% CI)	−0.26 (−0.58, 0.06)	−0.32 (−0.65, −0.00)	−0.52 (−0.92, −0.12)	−0.47 (−0.87, −0.08)	−0.50 (−0.82, −0.17)	NA
Uric acid change from baseline (μmol/L)	9.53 (−8.56, 27.62)	−0.53 (-19.07, 18.01)	0.23 (−26.45, 26.91)	−21.07 (-47.29, 5.16)	−10.60 (-29.31, 8.10)	15.05 (−3.33, 33.44)
ETD versus placebo (95% CI)	−5.52 (−31.31, 20.26)	−15.59 (−41.70, 10.53)	−14.82 (−47.23, 17.58)	−36.12 (−68.16, −4.08)	−25.66 (−51.89, 0.57)	NA
Total cholesterol change from baseline (mmol/L)	−0.31 (−0.47, −0.15)	−0.58 (−0.74, −0.42)	−0.49 (−0.73, −0.26)	−0.46 (−0.69, −0.23)	−0.48 (−0.64, −0.31)	−0.23 (−0.39, −0.06)
ETD versus placebo (95% CI)	−0.09 (−0.31, 0.14)	−0.36 (−0.58, −0.13)	−0.27 (−0.55, 0.02)	−0.23 (−0.51, 0.05)	−0.25 (−0.48, −0.02)	NA
Triglycerides change from baseline (mmol/L)	−0.05 (−0.21, 0.11)	−0.17 (−0.33, −0.00)	−0.48 (−0.71, −0.24)	−0.32 (−0.55, −0.08)	−0.39 (−0.56, −0.23)	0.11 (−0.06, 0.27)
ETD versus placebo (95% CI)	−0.15 (−0.38, 0.08)	−0.27 (−0.51, −0.04)	−0.58 (−0.87, −0.29)	−0.42 (−0.71, −0.14)	−0.50 (−0.73, −0.27)	NA
LDL-C change from baseline (mmol/L)	−0.08 (−0.21, 0.05)	−0.29 (−0.42, −0.16)	−0.24 (−0.42, −0.05)	−0.17 (−0.35, 0.02)	−0.20 (−0.33, −0.07)	−0.02 (−0.15, 0.11)
ETD versus placebo (95% CI)	−0.06 (−0.24, 0.12)	−0.27 (−0.45, −0.09)	−0.22 (−0.45, 0.01)	−0.15 (−0.37, 0.08)	−0.18 (−0.36, 0.00)	NA
HDL-C change from baseline (mmol/L)	−0.09 (−0.13, −0.05)	−0.11 (−0.15, −0.07)	−0.08 (−0.13, −0.02)	−0.16 (−0.21, −0.10)	−0.12 (−0.15, −0.08)	−0.03 (−0.07, 0.00)
ETD versus placebo (95% CI)	−0.06 (−0.11, −0.00)	−0.08 (−0.13, −0.03)	−0.04 (−0.11, 0.02)	−0.12 (−0.19, −0.06)	−0.08 (−0.13, −0.03)	NA

Data are *n* (%) or least square mean (95% CI) change from baseline to week 16, unless otherwise stated. Analysis was performed using an ANCOVA model, with baseline parameters as a covariate and treatment groups as explanatory variables. *: Missing body weight data at Week 16 were imputed using the MI method under the MAR assumption. †: Point estimates and 95% CIs for the event rate in each treatment group were calculated with the Clopper–Pearson exact method. &: The between-group rate difference and its 95% CI were computed using Newcombe’s method, and groups were compared with the χ² test. *CI* confidence interval, *ETD* estimated treatment difference, *BMI* body mass index, *HbA1c* glycated hemoglobin, *LDL-C* low-density lipoprotein cholesterol, *HDL-C* high-density lipoprotein cholesterol, *NA* not applicable, *ANCOVA* analysis of covariance, *MI* multiple imputation, *MAR* missing at random

At week 16, 55.4% (80 mg) to 90.3% (160 mg-ST) of participants treated with VCT220 achieved at least 5% weight loss, compared to 13.1% in the placebo group. The results were dose-dependent, with the following proportions of participants achieving ≥ 5% weight loss: 80 mg: 55.4%, 120 mg: 72.6%, 160 mg-FT: 77.4%, 160 mg-ST: 90.3%. For the ≥ 10% weight loss threshold, the response ranged from 4.6% (80 mg) to 45.2% (160 mg-FT) across VCT220 doses, while only 1.6% of placebo participants achieved this level. Similarly, 1.5% (80 mg) to 9.7% (160 mg-FT) of participants in the VCT220 groups achieved ≥ 15% weight loss, whereas no participants in the placebo group did so. These results demonstrate a clear dose-dependent effect of VCT220 on clinically meaningful weight loss (Fig. 2c, d). Only participants in the 160 mg-FT group (1.6%) achieved weight loss of 20% or more. Moreover, while the study duration of 16 weeks did not show a plateau in weight loss, the ongoing trends, particularly in the higher-dose groups, suggest that longer-term treatment may lead to even greater weight loss. Furthermore, the decrease in BMI and waist circumference was dose-dependent, with VCT220 consistently leading to greater reductions compared to the placebo group. The mean changes from baseline in BMI and waist circumference at week 16 ranged from –1.80 to –3.21 kg/m^2^ and from –5.15 to –7.61 cm, respectively, across VCT220 doses, whereas the values were –0.49 kg/m^2^ and –1.99 cm, respectively, in the placebo group (Table 2).

### Cardiometabolic parameters

**Fig. 3:**
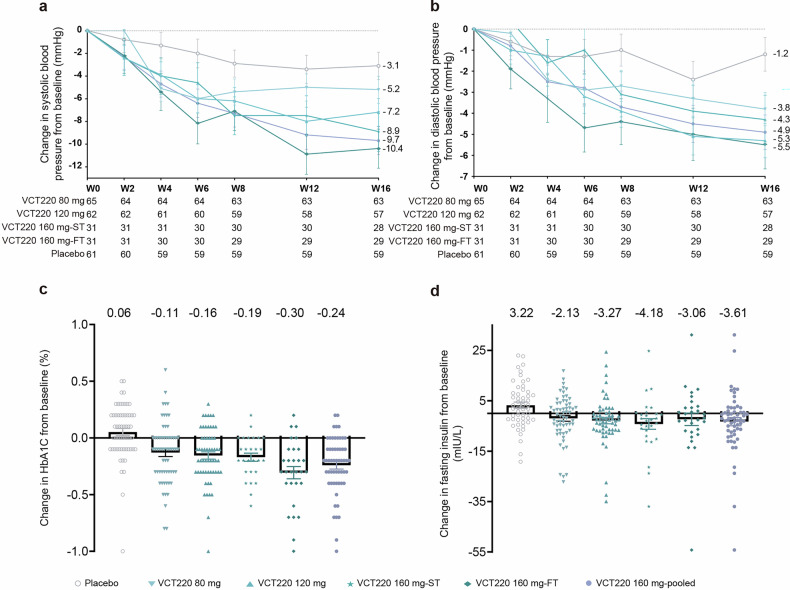
Comparison of cardiometabolic parameters with VCT220 versus placebo during the treatment period. **a** Observed mean change in systolic pressure (mmHg) over time. Numbers below the panels are the number of participants contributing to the mean. **b** Observed mean change in diastolic pressure (mmHg) over time. Numbers below the panels are the number of participants contributing to the mean. **c** Mean change in HbA1c (%) from baseline at week 16. d Mean change in fasting insulin (mIU/L) from baseline at week 16. All data were analyzed using FAS with multiple imputation. Changes from baseline data are reported as the least squares mean ± SE. ST slow titration, FT fast titration, HbA1c glycated hemoglobin A1c, SE standard error, FAS full analysis set

Improvements were also observed in several exploratory cardiometabolic parameters, including blood pressure, glycemic markers and lipid measures. At the end of treatment, participants receiving VCT220 showed mean reductions in systolic blood pressure (SBP) ranging from −5.2 mmHg to −9.7 mmHg and in diastolic blood pressure (DBP) ranging from −3.8 mmHg to −5.3 mmHg, whereas the corresponding reductions were −3.1 mmHg and −1.2 mmHg in the placebo group (Fig. 3a, b; supplementary Tables 4, 5). These improvements in the blood pressure did not result in significant changes in the heart rate until the end of treatment.

Significant improvements in HbA1c and fasting insulin were observed with VCT220 treatment compared to placebo (Fig. 3c, d; Table 2). HbA1c levels decreased from baseline to week 16 across all VCT220 dose groups, with more significant reductions than placebo. The estimated mean treatment differences were –0.17% for VCT220 80 mg, –0.21% for VCT220 120 mg, –0.25% for VCT220 160 mg-ST and –0.35% for VCT220 160 mg-FT. Fasting insulin concentrations decreased significantly in a dose-dependent manner as VCT220 dose levels increased. Higher doses of VCT220 tended to produce greater reductions in total cholesterol and triglycerides. The mean changes from baseline in total cholesterol and triglycerides at week 16 ranged from –0.31 to –0.58 mmol/L and from –0.05 to –0.48 mmol/L, respectively, across VCT220 doses, whereas the values were –0.23 mmol/L and 0.11 mmol/L, respectively, in the placebo group (Table 2).

### Safety outcomes

VCT220 was well tolerated across all dose groups, without any identified new safety signals. The proportion of participants reporting treatment-emergent adverse events (TEAEs) was similar between the VCT220 groups (89.2–100%) and the placebo group (93.4%) (Table 3). Treatment-related adverse events (TRAEs) of VCT220 were 78.5% for 80 mg, 91.9% for 120 mg, 80.6% for 160 mg-ST and 87.1% for 160 mg-FT, versus 60.7% for placebo. Most TEAEs and TRAEs were mild or moderate, transient, and manageable. Treatment discontinuation because of TEAEs occurred in one participant each in the 80 mg, 120 mg, 160 mg-FT, and placebo groups.

**Table 3:** Adverse events during the trial period (safety analysis set)

	VCT220 80 mg (*N* = 65)	VCT220 120 mg (*N* = 62)	VCT220 160mg-ST (*N* = 31)	VCT220 160mg-FT (*N* = 31)	VCT220 160mg-Pooled (*N* = 62)	Placebo Pooled (*N* = 61)
TEAEs, *n* (%)	58 (89.2)	62 (100.0)	28 (90.3)	31 (100.0)	59 (95.2)	57 (93.4)
Treatment-related AEs, *n* (%)	51 (78.5)	57 (91.9)	25 (80.6)	27 (87.1)	52 (83.9)	37 (60.7)
TEAEs leading to discontinuation, *n* (%)	1 (1.5)	1 (1.6)	0 (0.0)	1 (3.2)	1 (1.6)	1 (1.6)
Grade ≥3 TEAEs, *n* (%)	3 (4.6)	4 (6.5)	0 (0.0)	2 (6.5)	2 (3.2)	1 (1.6)
Treatment-related Grade ≥3 AEs, *n* (%)	0 (0.0)	2 (3.2)	0 (0.0)	0 (0.0)	0 (0.0)	0 (0.0)
SAE	2 (3.1)	1 (1.6)	0 (0.0)	0 (0.0)	0 (0.0)	0 (0.0)
Treatment-related SAE	0 (0.0)	0 (0.0)	0 (0.0)	0 (0.0)	0 (0.0)	0 (0.0)
Gastrointestinal TEAEs, *n* (%)	38 (58.5)	46 (74.2)	20 (64.5)	25 (80.6)	45 (72.6)	17 (27.9)
Nausea	27 (41.5)	26 (41.9)	14 (45.2)	18 (58.1)	32 (51.6)	5 (8.2)
Vomiting	9 (13.8)	20 (32.3)	11 (35.5)	13 (41.9)	24 (38.7)	5 (8.2)
Diarrhea	12 (18.5)	11 (17.7)	5 (16.1)	9 (29.0)	14 (22.6)	5 (8.2)
Abdominal bloating	3 (4.6)	4 (6.5)	3 (9.7)	2 (6.5)	5 (8.1)	0 (0.0)

Data are *n* (%). *TEAE* treatment-emergent adverse event, *AE* adverse event, *SAE* serious adverse event

Gastrointestinal adverse events (GI-AEs) were the most frequently reported AEs, with an overall incidence of 58.4% (146 participants). The incidence of GI-AEs increased in a dose-dependent manner, ranging from 58.5% at 80 mg to 74.2% at 120 mg and 72.6% at pooled 160 mg, compared to 27.9% in the placebo group, representing a 2.1- to 2.7-fold increase in GI-AE rates with VCT220 relative to placebo. Within the 160 mg dose group, the incidence of GI-AEs was significantly higher in participants receiving fast titration (80.6%) compared to placebo (27.9%). Notably, slow titration reduced this incidence to 64.5%, highlighting the impact of dosing strategy on tolerability. Nausea, vomiting, and diarrhea were the common GI-AEs with a dose-dependent manner in incidence. Nausea of VCT220 groups ranged from 41.5% (80 mg) to 58.1% (160 mg-FT), versus 8.2% in the placebo group. Vomiting of VCT220 groups ranged from 13.8% (80 mg) to 41.9% (160 mg-FT), versus 8.2% in the placebo group. Diarrhea of VCT220 groups ranged from 17.7% (120 mg) to 29.0% (160 mg-FT), versus 8.2% in the placebo group.

As expected for GLP-1RAs, the incidence of GI-AEs peaked during the dose titration phase (weeks 4-6) and decreased from week 4 to week 16. Most GI-AEs were mild (grade 1, 50.4%) or moderate (grade 2, 17.2%), with only the 120 mg group experiencing three instances (1.2%) of grade 3 GI-AEs. Importantly, permanent discontinuation due to gastrointestinal events was rare, occurring in only two participants across all VCT220 groups. VCT220 treatment reduced alanine aminotransferase (ALT) and aspartate aminotransferase (AST) levels, with more significant reductions observed in the 120 mg and 160 mg groups than in the 80 mg and placebo groups (supplementary Tables 6, 7). No significant safety concerns related to liver function were identified. As for hypoglycemic events, a total of 9 episodes among 5 (2.0%) participants were reported, with 1 participant in the placebo group, 1 participant in the 80 mg group, 2 participants in the 120 mg group, and 1 participant in the 160 mg group, respectively. All events were mild, and no severe hypoglycemia was reported. There were no significant differences in the incidence of hypoglycemia between groups.

Cardiovascular events were infrequent and mild (Grade 1), comparable between the VCT 220 groups and placebo group, with palpitation being the most common event. The proportion of different degrees of change in QTcF relative to baseline (ΔQTcF) in all VCT220 groups showed no significant difference from that in the placebo group, suggesting that VCT220 tablets do not increase the risk of QT interval prolongation. An increase in the calcitonin levels was rare, mild, transient, and resolved without intervention. No significant safety concerns were identified in the renal, hepatic, gallbladder, or psychiatric assessments. Additionally, no deaths and drug-related SAEs occurred during the trial.

## Discussion

In this randomized, placebo-controlled phase II trial, the nonpeptide oral GLP-1RA VCT220 produced dose-dependent and clinically meaningful reductions in body weight, waist circumference, HbA1c, fasting insulin, total cholesterol, triglycerides and blood pressure over 16 weeks in adults with overweight or obesity without diabetes. The magnitude of weight loss was notable given the short treatment duration, and the high proportion of participants achieving ≥5% and ≥10% weight loss reinforces the potential clinical utility of this agent.

Injectable GLP-1RAs typically achieve peak weight loss after 40-68 weeks.^8–13^ Similarly, orforglipron, another nonpeptide GLP-1RAs, resulted in a body weight reduction of 11.2%, with a placebo-corrected reduction of 9.1% in participants with obesity at week 72 at the highest dose of 36 mg in phase III trial ATTAIN−1.^11^ In phase III trial OASIS1, Oral semaglutide 50 mg showed a treatment difference of -12.7% of mean body weight change from baseline compared with placebo at week 68 in adults with overweight or obesity without type 2 diabetes.^6^ As a once-daily, orally administered, nonpeptide agent, VCT220 eliminates the need for injections, which may improve patient acceptance, convenience, and long-term adherence. In this phase II trial, VCT220 reduced weight in a dose-dependent manner and achieved a weight reduction of 9.73% in the highest 160 mg-FT group without an apparent plateau at week 16.

The safety profile was consistent with other GLP-1RAs, with GI-AEs such as nausea, vomiting and diarrhea being the most common TEAEs.^10,14–16^ GI-AEs were dose-dependent and predominantly occurred during the dose-escalation phase, particularly in the first four weeks. The rapid titration group (160 mg-FT) showed higher GI-AE rates than the slow titration group (160 mg-ST), suggesting that slower titration improves tolerability—a finding consistent with other GLP-1RA trials.^11,17–20^ Additionally, SBP and DBP reductions ranged from −5.2 to −9.7 mmHg and from −3.8 to −5.3 mmHg, respectively, surpassing placebo (−3.1 mmHg and −1.2 mmHg), which may suggest a potentially greater antihypertensive effect with VCT220 compared to other GLP-1 receptor agonists, a finding that warrants confirmation in further studies.^21^ The incidence of hypoglycemic events was infrequent, with no severe cases reported, consistent with the known safety profiles of this drug class. Discontinuation due to AEs was rare, occurring in only one participant from each of the VCT220 80 mg, 120 mg, and 160 mg groups and the placebo group. No significant safety concerns were identified in the renal, hepatic, gallbladder, or psychiatric assessments, comparable with semaglutide, dulaglutide, and orforglipron.^11,15,19,20^ Furthermore, the high trial completion rate (>94%) and low discontinuation rates strengthen the robustness of these results.

Oral nonpeptide, small-molecule GLP-1RAs may differ from oral peptide GLP-1RAs (e.g., oral semaglutide) in receptor engagement, desensitization, and tissue distribution.^22^ In contrast to oral semaglutide, which requires absorption enhancers and strict fasting administration, VCT220 does not depend on specialized delivery technologies and can be taken with meals.^6^ VCT220 also showed slow systemic clearance, prolonging plasma exposure and restricted tissue distribution, reducing off-target retention.^23^ While the extent of CNS penetration and long-term receptor kinetics is not fully characterized, our in-house preliminary animal experiments have indicated measurable but low brain concentrations of VCT220. These findings support the hypothesis of combined central and peripheral metabolic effects of GLP-1RAs, meriting confirmation in future studies. The good tolerability and favorable dose-dependent efficacy of VCT220 provide a strong rationale for testing higher doses in future trials, aiming to fully explore its therapeutic potential.

This study had several limitations. The 16-week duration, along with the time required to reach the target dose, especially for participants in the VCT220 160 mg slow titration group who did not reach the target dose until week 7, may not have fully captured the weight loss potential or long-term safety profile of VCT220. The U.S. FDA and EMA require anti-obesity drugs to demonstrate ≥5% placebo-subtracted weight loss sustained over at least one year, along with an acceptable cardiovascular and metabolic safety profile. While the observed weight loss over just 16 weeks is promising for a short-term proof of concept, information on durability, plateau, and long-term safety is limited, as well as the comparisons with other GLP-1RA trials should be interpreted cautiously. Longer-term trials for at least one year are essential to determine whether VCT220 meets these regulatory benchmarks. The favorable tolerability and low discontinuation rate in this trial provide preliminary support for proceeding to phase III evaluation. Improvements of numerous metabolic parameters were exploratory results, which also need to be further confirmed in phase III evaluation with a prespecified hierarchy for the analysis. Besides, this multi-center phase II trial explored several different titration algorithms with corresponding placebo, leading to potential bias raised from pooled placebo cohorts, site effects and the confounding introduced by different titration cadences. Moreover, the study participants were predominantly Han Chinese under Chinese criteria of overweight and obesity, limiting the generalizability of the findings to more diverse ethnic groups by using different obesity and overweight diagnosis criteria (BMI ≥ 30 kg/m^2^ or ≥ 27 kg/m^2^ with comorbidity). The low burden of baseline comorbidities seen in East Asian obesity populations may also limit extrapolation to Western populations with higher cardiometabolic risk. Furthermore, pharmacological responses to GLP-1RAs may vary by ethnicity due to differences in body composition, insulin sensitivity, and genetic factors. Additionally, as is typical for phase II trials, participants with significant comorbidities, such as advanced heart failure (New York Heart Association class 3 or 4) or severe renal dysfunction (eGFR <60 mL/min/1.73 m^2^), were excluded to ensure safety and minimize confounding factors. As cardiovascular outcomes are crucial for the long-term safety evaluation, future trials should incorporate longer follow-up, cardiovascular endpoints, and stratification by baseline cardiometabolic risk. While these exclusions are standard in early-phase trials, future studies should include multi-ethnic cohorts with broader age ranges and comorbidity profiles to assess the real-world effectiveness and safety of VCT220 across diverse populations. Reductions in ALT and AST across VCT220 dose groups were noteworthy for hepatic safety and potential benefits, while long-term studies incorporating liver imaging or histologic assessments are necessary for liver evaluation in metabolic dysfunction–associated steatotic liver disease (MASLD) participants. Despite these limitations, this study provided valuable early insights into the efficacy and safety of VCT220, serving as a framework for further studies on more representative and international cohorts.

In summary, this phase 2 study in individuals with overweight or obesity without type 2 diabetes demonstrated that 16 weeks of treatment with VCT220, a nonpeptide GLP-1 receptor agonist, at doses of 80–160 mg, as an adjunct to lifestyle interventions, resulted in dose-dependent and clinically significant body weight reductions compared to placebo. VCT220 exhibited a safety and tolerability profile consistent with other GLP-1RAs. These findings strongly support the further development of VCT220 as a novel pharmacotherapeutic agent for weight management.

## Materials and Methods

### All the ethics approval statements

This study was approved by an ethics committee (Ethics Committee of Peking University People’s Hospital, No. 2023PHA139-001). All participants provided written informed consent prior to participation in this study. The study adhered to the ICH-GCP guidelines, the Declaration of Helsinki (2008), the Good Clinical Practice for Drugs, and all other relevant laws and regulations. This trial was registered under the identifier CTR20233978 (www.chinadrugtrials.org.cn) and Clinicaltrial.gov (NCT06569355). The reporting of this trial conforms to CONSORT 2010 guidelines. The study was executed in accordance with the “Technical Guidelines for Clinical Trials of Weight Control Drugs” issued by the Center for Drug Evaluation (CDE) of the National Medical Products Administration (NMPA) of China (2021, https://english.nmpa.gov.cn/) (supplementary Text).

### Study design

This phase II trial was a multicenter, randomized, double-blind, placebo-controlled, dose-finding study conducted at 13 clinical sites across China (Fig. 1). The study consisted of a 16-week treatment period, including a dose-escalation phase lasting for four to six weeks, followed by a two-week treatment-free follow-up period. The study protocol and all amendments were reviewed and approved by institutional review boards (IRBs) at participating sites.

### Participants

Eligible participants were male and female adults aged 18–75 years who were overweight (BMI 24–28 kg/m2) with at least one predefined comorbid condition, or obese (BMI ≥ 28 kg/m2), with or without comorbidities. Eligible comorbidities included prediabetes (impaired fasting glucose and/or impaired glucose tolerance), hypertension, dyslipidemia, fatty liver, or overweight-associated obstructive sleep apnea syndrome, but without type 1 or type 2 diabetes. Based on self-reported data, participants underwent at least 12 weeks of diet and exercise interventions before screening and demonstrated stable weight within 12 weeks before screening, defined as a weight fluctuation of less than 5% ([Maximum weight - Minimum weight]/Maximum weight). Eligibility criteria are described in (supplementary Text).

### Randomization and masking

Participants were allocated to three dose cohorts (80 mg, 120 mg, or 160 mg). The 160 mg group was further divided into rapid-titration (VCT220 160 mg-FT) and slow-titration (VCT220 160 mg-ST) subgroups. Participants were then randomly assigned at a 3:1 ratio to receive oral administration of VCT220 once daily or a matching placebo. Block randomization was implemented within each cohort to ensure balance between treatment arms, given the exploratory nature and modest sample size of this phase II trial.

To maintain blinding, the sponsor or its designated unit ensured that both the investigational drug and placebo were identical in packaging, appearance, odor, and color. The Interactive Web Response System (IWRS) was used for randomization and data storage. Blinding at the study site was managed by the study statistician personnel who did not participate in the trial’s administration. These individuals labeled and numbered the investigational drugs and placebos according to the random allocation table. The blinding process was carefully documented and archived, with the full details of the process exported after unblinding.

### Procedures

The study included a 2-week screening period, a 16-week treatment phase, and a 2-week safety follow-up. Participants were randomly assigned to receive either oral VCT220 or a dose-matched placebo, with instructions to take the medication with meals, preferably at breakfast. All four placebo groups were pooled into a single placebo group for primary analyses.

VCT220 treatment was initiated at randomization, followed by weekly dose escalation until the target dose was reached. For participants in the 160 mg slow-titration group, the dose was increased every two weeks during the first four weeks (supplementary Fig. 1). All participants received dietary and physical activity counseling, which aimed for a daily 500 kcal deficit and adherence to a fixed physical activity regimen. This regimen included aerobic exercise five times weekly and resistance training twice weekly. Body weight was assessed at screening, randomization, weeks 2, 4, 6, 8, 12, 16, and follow-up. Further details of the efficacy and safety assessments conducted at each visit are outlined in the study protocol (supplementary Study protocol).

### Outcomes

The primary endpoint was the percentage change in body weight from baseline to week 16. Secondary efficacy endpoints included the proportion of participants who achieved body weight reductions of at least 5%, 10%, 15%, and 20% from baseline at weeks 8, 12, and 16; the percentage change in body weight from baseline at weeks 8 and 12; and changes from baseline to week 16 in absolute body weight (kg), BMI (kg/m^2^), waist circumference (cm), lipid parameters (total cholesterol, HDL, LDL, and triglycerides), uric acid (μmol/L), glycemic variables (HbA1c, fasting plasma glucose, fasting insulin, and fasting C-peptide), and blood pressure (SBP and DBP).

Safety and tolerability endpoints included the frequency and nature of TEAEs and serious adverse events (SAEs), along with changes in safety laboratory variables, electrocardiograms, and vital signs. Adverse events and vital signs were assessed at every study visit.

### Statistical analysis

All statistical analyses were conducted using the SAS software version 9.4 or higher. The primary statistical analysis was conducted on the full analysis set (FAS), which included all randomized participants who were administered at least one dose of the study medication. For participants who discontinued treatment for any reason before the primary endpoint visit, follow-up was continued, and relevant data were collected. For participants who were under medication that may affect treatment efficacy before the primary endpoint visit, follow-up was also continued, and these data were included in the analysis. The per-protocol set (PPS) was used for supportive analysis, including only those participants who completed the study as per the protocol, with no major protocol violations that could affect the efficacy results, and for whom the primary efficacy endpoint could be measured. The safety set (SS) consisted of all participants who were administered at least one dose of the study drug, which was used to analyze safety data.

For efficacy analyses, changes from baseline in weight were assessed using analysis of covariance (ANCOVA), with the treatment group as the independent variable and the baseline weight as a covariate. For group comparisons, the least squares mean (LSM) difference between the treatment groups and the placebo group, along with its 95% confidence interval (CI), was calculated to evaluate the treatment effect. The Newcombe hybrid score method was used for categorical variables to determine the difference in risk between groups and its 95% confidence interval. Missing data in the primary analysis using ANCOVA were imputed by multiple imputation (MI) methods under the missing at random (MAR) assumption.^24^ Sensitivity analysis model 1 using mixed-effects models for repeated measures (MMRM) without missing data imputation was performed to assess the robustness of the results. Sensitivity analysis model 2, using ANCOVA with site effects and titration regimen as model factors, was also explored.

AEs were coded for safety analysis using the Medical Dictionary for Regulatory Activities (MedDRA 27.0) and graded according to the Common Terminology Criteria for Adverse Events version 5.0 (CTCAE v5.0) into Grade 1 to Grade 5, and incidence rates were calculated for each treatment group. The analysis focused on TEAEs, with subgroup analysis for serious TEAEs and TEAEs leading to the discontinuation of treatment.

## Supplementary information

Supplementary materials

Study protocol

## Data Availability

The study protocol, including statistical analysis plan, and other supporting materials are available within this article and its supplementary files. The datasets generated and/or analyzed during the current study are available from the corresponding author for non-commercial, academic purposes. All data-sharing requests will be reviewed by the corresponding author and the sponsor, Chengdu Vincentage Pharma Co., within 2 weeks of submission. Approved requests will require a signed data access agreement with the sponsor prior to the release of any data.
