# Supplementary material for: Oral nonpeptide GLP-1 receptor agonist VCT220 for obesity treatment: a randomized, double-blind, phase II trial
Source: Signal Transduct Target Ther. 2026 Aug 14;11:325. doi: 10.1038/s41392-026-02859-2 (PMC13473623; doi:10.1038/s41392-026-02859-2)
Supplement: Supplementary file 1 [file 41392_2026_2859_MOESM1_ESM.docx]

Supplementary Materials for

Oral Nonpeptide GLP-1 Receptor Agonist VCT220 for Obesity Treatment: a Randomized, Double-blind, Phase II Trial

Linong Ji^1^*^#^, Leili Gao^1#^, Ben Li^2^, Mei Liu^2^, Xianghua Zhang^3^, Chao Meng^4^

Correspondence to: [jiln@bjmu.edu.cn](mailto:jiln@bjmu.edu.cn)

**This PDF file includes:**

Supplementary Text

Figure. S1

Tables S1 to S11

**Other Supplementary Materials for this manuscript include the following:**

Study protocol

**Supplementary Text**

**Study objectives, protocol summary, and ethics review approval**

**Study objectives**

1. Primary objective

To evaluate the weight reduction efficacy of different doses of VCT220 tablets in overweight or obese non-diabetic subjects.

2. Secondary objectives

To assess the safety profile of different doses of VCT220 tablets in overweight or obese non-diabetic subjects.

To evaluate the pharmacokinetic (PK) characteristics of different doses of VCT220 tablets.

3. Exploratory objective

To explore the correlation between VCT220 exposure, observed weight loss efficacy, and incidence of adverse events (AEs), providing insights into dose-response and safety relationships.

**Protocol summary**

1. Study design

This study was a multicenter, randomized, double-blind, placebo-controlled, Phase II clinical trial.

Screening phase (up to 2 weeks): Participants underwent initial assessments, including physical exams, laboratory tests, and baseline data collection.

Treatment phase (16 weeks): Subjects received VCT220 or placebo daily, with the dose titrated to predetermined levels.

Safety follow-up phase (2 weeks): Monitoring for delayed or persistent adverse events after the treatment phase.

Randomization: A total of 240 subjects were planned, randomly assigned in a 3:1 ratio to treatment and placebo groups.

The study included parallel groups for multiple dose levels (80 mg, 120 mg, and 160 mg), and 160mg group further divided into fast titraton (FT) and slow titration (ST) arms.

2. Dosing regimen

VCT220 or dose-matched placebo was administered orally once daily with meals, preferably at breakfast. Dose-titration regimen is detailed below:

| Group | Subjects (N) | Week 1 | Week 2 | Week 3 | Week 4 | Week 5 | Week 6 | Weeks 7–16 |
| --- | --- | --- | --- | --- | --- | --- | --- | --- |
| 80 mg dose group | 60 | 20 mg | 40 mg | 80 mg | 80 mg | 80 mg | 80 mg | 80 mg |
| 80 mg placebo group | 20 | 20 mg | 40 mg | 80 mg | 80 mg | 80 mg | 80 mg | 80 mg |
| 120 mg dose group | 60 | 20 mg | 40 mg | 60 mg | 80 mg | 120 mg | 120 mg | 120 mg |
| 120 mg placebo group | 20 | 20 mg | 40 mg | 60 mg | 80 mg | 120 mg | 120 mg | 120 mg |
| 160 mg rapid titration group | 30 | 20 mg | 40 mg | 80 mg | 120 mg | 160 mg | 160 mg | 160 mg |
| 160 mg placebo group (rapid) | 10 | 20 mg | 40 mg | 80 mg | 120 mg | 160 mg | 160 mg | 160 mg |
| 160 mg slow titration group | 30 | 20 mg | 20 mg | 40 mg | 40 mg | 80 mg | 120 mg | 160 mg |
| 160 mg placebo group (slow) | 10 | 20 mg | 20 mg | 40 mg | 40 mg | 80 mg | 120 mg | 160 mg |

3. Safety follow-up

All subjects were monitored for 2 weeks after the treatment phase to identify delayed adverse events, ensuring comprehensive safety evaluation.

4. Eligibility criteria

| Key Inclusion | 1. Male or female subjects aged 18-75 years (inclusive) when signing the informed consent form.. |
| --- | --- |
|  | 2. BMI: ≥28 kg/m² with or without comorbidities, or 24–28 kg/m² with at least one of the following comorbidities: prediabetes (impaired fasting glucose and/or impaired glucose tolerance), hypertension, dyslipidemia, fatty liver, or overweight-associated obstructive sleep apnea syndrome. |
|  | 3. Subjects who have undergone diet control or exercise control alone for at least 12 weeks before screening, resulting in a stable weight within 12 weeks before screening (weight change < 5%, weight change = [maximum weight - minimum weight]/maximum weight; based on subjects' self-report). |
|  | 4. Subjects who have no fertility plan and voluntarily take effective contraceptive measures without any plans to donate sperm or eggs during the study and within 90 days after the end of study. |
|  | 5. Subjects who are willing and able to maintain a stable diet and exercise during the study and fill in diary cards on time. |
|  | 6. Subjects who are able to understand the procedures and methods of this study, willing to complete this study in strict accordance with the clinical study protocol, and voluntarily sign the informed consent form. |
| Key Exclusion | 1. Pre-existing endocrinopathy or monogenic obesity, including but not limited to hypothalamic obesity, pituitary obesity, hypothyroid obesity, Cushing’s syndrome, insulinoma, acromegaly, or hypogonadism. |
|  | 2. Prior bariatric or weight-reduction surgery (except acupuncture for weight loss, liposuction, or abdominal lipectomy performed >1 year before screening), or planned surgical treatment for obesity during the study, such as gastric bypass, gastric banding, etc. |
|  | 3. Currently on a weight-loss program and not yet in the maintenance phase at screening (based on self-report). |
|  | 4. Previous diagnosis of type 1, type 2, or any other specific type of diabetes. |
|  | 5. Prior use of any glucagon-like peptide-1 receptor agonist (GLP-1 RA), GLP-1-related multi-target agonists (e.g., GLP-1/glucose-dependent insulinotropic polypeptide [GIP] dual agonist, GLP-1/glucagon receptor [GCGR] dual agonist), or combination products containing a GLP-1 agonist. |
|  | 6. Laboratory Abnormalities: HbA1c ≥6.5%, fasting glucose ≥7.0 mmol/L, or eGFR <60 mL/min/1.73m². |
|  | 7. Within 12 weeks before screening, use of any of the following drugs or treatments: |

**Ethics review approval**

**Figure. S1.** Dosage and endpoints. ST slow titration, FT fast titration

**Table S1.** Least square mean (LSM) and standard error (SE) of the percentage change in body weight from baseline at each planned visit - FAS

| Visit | VCT220 80mg  (N=65) | VCT220 120mg  (N=62) | VCT220 160mg-ST  (N=31) | VCT220 160mg-FT  (N=31) | VCT220 160mg-pooled  (N=62) | Placebo  (N=61) |
| --- | --- | --- | --- | --- | --- | --- |
| V1 (Baseline) | - | - | - | - | - | - |
| V2 | -0.875 (0.162) | -1.113 (0.165) | -1.241 (0.233) | -1.596 (0.234) | -1.418 (0.165) | -0.391 (0.166) |
| V3 | -2.242 (0.220) | -2.239 (0.225) | -2.180 (0.316) | -3.462 (0.317) | -2.821 (0.223) | -0.761 (0.229) |
| V4 | -2.977 (0.271) | -3.521 (0.279) | -3.859 (0.389) | -5.001 (0.396) | -4.421 (0.277) | -1.050 (0.282) |
| V5 | -3.458 (0.336) | -4.466 (0.347) | -5.355 (0.486) | -6.228 (0.487) | -5.791 (0.344) | -1.165 (0.346) |
| V6 | -4.640 (0.426) | -6.130 (0.444) | -7.602 (0.617) | -8.808 (0.629) | -8.195 (0.440) | -1.323 (0.440) |
| V7 | -5.751 (0.487) | -7.424 (0.502) | -9.404 (0.707) | -9.725 (0.730) | -9.496 (0.504) | -1.610 (0.500) |
| V8 (Follow up) | -5.266 (0.496) | -7.195 (0.522) | -8.488 (0.745) | -9.260 (0.733) | -8.881 (0.522) | -1.505 (0.513) |

Analysis was performed using an ANCOVA model, with baseline body weight as a covariate and treatment groups as explanatory variables. Missing body weight data at Week 16 (Visit 7) were imputed using the MI method under the MAR assumption, while no imputation was applied for body weight data at other visits. *FAS* full analysis set, *ANCOVA* analysis of covariance, *MI* multiple imputation, *MAR* missing at random

| **Table S2.** Sensitivity analysis 1 of the primary endpoint (MMRM): percentage change in body weight from baseline to week 16 - FAS | | | | | | |
| --- | --- | --- | --- | --- | --- | --- |
|  | VCT220 80mg (N=65) | VCT220 120mg (N=62) | VCT220 160mg -ST (N=31) | VCT220 160mg-FT (N=31) | VCT220 160mg-pooled (N=62) | Placebo  (N=61) |
| LSM (SE) of percentage change in body weight | -5.709 (0.357) | -7.537 (0.365) | -9.891 (0.438) | -9.644 (0.434) | -9.766 (0.366) | -1.623 (0.366) |
| 95% CI for LSM of percentage change | (-6.411, -5.007) | (-8.255, -6.820) | (-10.752, -9.031) | (-10.497, -8.792) | (-10.484, -9.048) | (-2.341, -0.904) |
| Estimated difference in LSM vs. Placebo | -4.086 | -5.915 | -8.269 | -8.022 | -8.143 |  |
| 95% CI for estimated difference vs. Placebo | (-5.091, -3.082) | (-6.930, -4.899) | (-9.389, -7.148) | (-9.136, -6.907) | (-9.159, -7.128) |  |
| P-value vs. Placebo | <0.001 | <0.001 | <0.001 | <0.001 | <0.001 |  |
| Analysis was performed using a MMRM model, with baseline body weight as a covariate, and treatment group, scheduled visit, and the treatment-by-visit interaction as fixed effects. *FAS* full analysis set, *LSM* least-squares mean, *SE* standard error, *CI*, confidence interval, *MMRM* mixed-effects model for repeated measures | | | | | | |

| **Table S3.** Sensitivity analysis 2 of the primary endpoint (ANCOVA with site effects and titration regimen as model factors): percentage change in body weight from baseline to week 16 - FAS | | | | | | |
| --- | --- | --- | --- | --- | --- | --- |
|  | VCT220 80mg (N=65) | VCT220 120mg (N=62) | VCT220 160mg -ST (N=31) | VCT220 160mg-FT (N=31) | VCT220 160mg-pooled (N=62) | Placebo  (N=61) |
| LSM (SE) of percentage change in body weight | -5.760 (0.495) | -7.412 (0.510) | -9.386 (0.714) | -9.746 (0.732) | -9.498 (0.512) | -1.600 (0.508) |
| 95% CI for LSM of percentage change | (-6.730, -4.790) | (-8.411, -6.412) | (-10.787, -7.986) | (-11.181, -8.311) | (-10.501, -8.495) | (-2.596, -0.605) |
| Estimated difference in LSM vs. Placebo | -4.160 | -5.812 | -7.786 | -8.146 | -7.897 |  |
| 95% CI for estimated difference vs. Placebo | (-5.528, -2.792) | (-7.198, -4.425) | (-9.482, -6.091) | (-9.874, -6.418) | (-9.286, -6.509) |  |
| P-value vs. Placebo | <0.001 | <0.001 | <0.001 | <0.001 | <0.001 |  |
| Analysis was performed using an ANCOVA model, with baseline body weight as a covariate, treatment group as the explanatory variable, and center and titration regimen included as random effects. Missing body weight data at Week 16 will be imputed using MI under the MAR assumption. *FAS* full analysis set, *LSM* least-squares mean, *SE* standard error, *CI* confidence interval, *ANCOVA* analysis of covariance, *MI* multiple imputation, *MAR* missing at random | | | | | | |

| **Table S4.** Least square mean (LSM) and standard error (SE) of change from baseline in SBP (mmHg) at each planned visit - FAS | | | | | | |
| --- | --- | --- | --- | --- | --- | --- |
| Visit | VCT220 80mg (N=65) | VCT220 120mg (N=62) | VCT220 160mg -ST (N=31) | VCT220 160mg-FT (N=31) | VCT220 160mg-pooled (N=62) | Placebo  (N=61) |
| V1 (Baseline) | - | - | - | - | - | - |
| V2 | -0.0 (1.08) | -2.4 (1.10) | -2.4 (1.55) | -2.2 (1.56) | -2.3 (1.10) | -0.8 (1.12) |
| V3 | -5.1 (1.13) | -3.9 (1.15) | -4.0 (1.62) | -5.4 (1.64) | -4.7 (1.15) | -1.3 (1.17) |
| V4 | -6.0 (1.22) | -6.0 (1.26) | -4.6 (1.78) | -8.2 (1.78) | -6.4 (1.26) | -2.0 (1.27) |
| V5 | -5.4 (1.15) | -6.2 (1.19) | -7.5 (1.67) | -7.1 (1.70) | -7.3 (1.19) | -2.9 (1.19) |
| V6 | -5.0 (1.20) | -8.0 (1.25) | -7.5 (1.76) | -10.9 (1.76) | -9.2 (1.25) | -3.4 (1.24) |
| V7 | -5.2 (1.19) | -7.2 (1.22) | -8.9 (1.76) | -10.4 (1.73) | -9.7 (1.23) | -3.1 (1.21) |
| V8 (Follow up) | -5.5 (1.06) | -7.2 (1.11) | -8.9 (1.59) | -7.7 (1.56) | -8.3 (1.11) | -4.3 (1.09) |
| Analysis was performed using an ANCOVA model, with baseline SBP as a covariate and treatment groups as explanatory variables. *SBP* systolic blood pressure, *FAS* full analysis set, *ANCOVA* analysis of covariance | | | | | | |

| **Table S5.** Least square mean (LSM) and standard error (SE) of change from baseline in DBP (mmHg) at each planned visit - FAS | | | | | | |
| --- | --- | --- | --- | --- | --- | --- |
| Visit | VCT220 80mg (N=65) | VCT220 120mg (N=62) | VCT220 160mg-ST (N=31) | VCT220 160mg-FT (N=31) | VCT220 160mg-pooled (N=62) | Placebo  (N=61) |
| V1 (Baseline) | - | - | - | - | - | - |
| V2 | -0.2 (0.65) | -1.0 (0.66) | 0.4 (0.93) | -1.9 (0.94) | -0.8 (0.66) | -0.6 (0.67) |
| V3 | -2.4 (0.77) | -1.3 (0.79) | -1.6 (1.11) | -3.3 (1.13) | -2.5 (0.79) | -1.3 (0.80) |
| V4 | -2.9 (0.78) | -3.2 (0.81) | -1.0 (1.14) | -4.7 (1.14) | -2.8 (0.81) | -1.3 (0.81) |
| V5 | -2.7 (0.74) | -3.9 (0.76) | -3.1 (1.07) | -4.4 (1.09) | -3.7 (0.76) | -1.0 (0.76) |
| V6 | -3.3 (0.84) | -5.1 (0.88) | -3.9 (1.24) | -5.0 (1.24) | -4.5 (0.88) | -2.4 (0.87) |
| V7 | -3.8 (0.79) | -5.3 (0.81) | -4.3 (1.16) | -5.5 (1.14) | -4.9 (0.82) | -1.2 (0.80) |
| V8(Follow up) | -4.1 (0.77) | -6.5 (0.81) | -6.3 (1.15) | -7.3 (1.13) | -6.8 (0.81) | -2.6 (0.79) |
| Analysis was performed using an ANCOVA model, with baseline DBP as a covariate and treatment groups as explanatory variables. *DBP* diastolic blood pressure, *FAS* full analysis set, *ANCOVA* analysis of covariance | | | | | | |

**Table S6.** Mean and standard deviation (SD) of change from baseline in ALT (U/L) from baseline at each planned visit - SS

| Visit | VCT220 80mg  (N=65) | VCT220 120mg  (N=62) | VCT220 160mg-ST  (N=31) | VCT220 160mg-FT  (N=31) | VCT220 160mg-pooled  (N=62) | Placebo  (N=61) |
| --- | --- | --- | --- | --- | --- | --- |
| V1 (Baseline) | - | - | - | - | - | - |
| V2 | 0.99 (20.741) | -3.33 (13.044) | -3.01 (9.011) | -9.47 (12.026) | -6.24 (11.031) | -0.37 (14.680) |
| V3 | -1.73 (18.082) | -5.98 (12.592) | -6.55 (11.894) | -11.07 (18.960) | -8.78 (15.801) | -1.05 (16.104) |
| V4 | -6.2 (15.247) | -8.06 (13.721) | -8.02 (11.235) | -14.54 (16.197) | -11.28 (14.206) | -2.65 (16.199) |
| V5 | -4.11 (21.065) | -9.16 (12.053) | -9.20 (12.790) | -12.80 (19.767) | -10.97 (16.545) | -4.09 (10.904) |
| V6 | -7.66 (18.179) | -11.91 (14.971) | -9.94 (11.479) | -12.24 (30.176) | -11.09 (22.658) | -4.10 (12.857) |
| V7 | -4.62 (16.420) | -11.93 (16.783) | -10.51 (17.726) | -11.55 (28.105) | -11.04 (23.382) | -2.97 (14.314) |
| V8 (Follow up) | -2.39 (19.804) | -9.00 (18.678) | -9.11 (15.324) | -8.38 (25.609) | -8.74 (21.006) | -1.14 (15.094) |
| **Descriptive statistics was used to summarize, by treatment group, data at each scheduled visit and change from baseline for ALT.** Baseline value is defined as the last available value prior to randomization; change from baseline is defined as the difference between the measured value obtained and the baseline value. *ALT* alanine aminotransferase, *SS* safety analysis set | | | | | | |

**Table S7.** Mean and standard deviation (SD) of change from baseline in AST (U/L) from baseline at each planned visit - SS

| Visit | VCT220 80mg  (N=65) | VCT220 120mg  (N=62) | VCT220 160mg-ST  (N=31) | VCT220 160mg-FT  (N=31) | VCT220 160mg-pooled  (N=62) | Placebo  (N=61) |
| --- | --- | --- | --- | --- | --- | --- |
| V1 (Baseline) | - | - | - | - | - | - |
| V2 | 5.19 (32.980) | -1.71 (6.274) | -0.92 (6.765) | -5.35 (11.364) | -3.14 (9.539) | -0.16 (7.150) |
| V3 | 0.32 (9.441) | -3.18 (5.528) | -1.57 (6.216) | -5.99 (13.493) | -3.75 (10.596) | -0.48 (6.939) |
| V4 | -3.03 (6.621) | -3.46 (5.709) | -2.89 (5.553) | -7.13 (13.040) | -5.01 (10.164) | -1.06 (7.516) |
| V5 | -1.82 (7.503) | -4.21 (7.668) | -3.36 (6.726) | -5.69 (10.842) | -4.50 (8.986) | -2.06 (5.957) |
| V6 | -2.35 (9.467) | -5.20 (9.491) | -3.03 (5.487) | -5.06 (13.901) | -4.05 (10.524) | -1.59 (6.349) |
| V7 | -2.60 (8.017) | -4.72 (10.128) | -3.64 (7.136) | -4.36 (12.320) | -4.01 (10.029) | -0.95 (6.905) |
| V8 (Follow up) | -1.12 (6.751) | -4.54 (11.327) | -3.15 (7.525) | -4.47 (10.623) | -3.82 (9.174) | 0.60 (7.890) |
| **Descriptive statistics was used to summarize, by treatment group, data at each scheduled visit and change from baseline for AST.** Baseline value is defined as the last available value prior to randomization; change from baseline is defined as the difference between the measured value obtained and the baseline value. *AST* aspartate aminotransferase, *SS* safety analysis set | | | | | | |

**Table S8.** Percent change in body weight from baseline at 16 weeks by baseline BMI strata based on Chinese criteria for obesity subgroup - FAS

| BMI ≥ 28 kg/m^2^ | VCT220 80mg  (N=61) | VCT220 120mg  (N=57) | VCT220 160mg-ST  (N=30) | VCT220 160mg-FT  (N=28) | VCT220 160mg-pooled  (N=58) | Placebo  (N=58) |
| --- | --- | --- | --- | --- | --- | --- |
| LSM (SE) of percentage change in body weight | -5.837 (0.517) | -7.400 (0.542) | -9.336 (0.737) | -9.561 (0.794) | -9.363 (0.535) | -1.611 (0.527) |
| 95% CI for LSM of percentage change | (-6.850, -4.824) | (-8.462, -6.338) | (-10.781, -7.891) | (-11.116, -8.005) | (-10.412, -8.313) | (-2.644, -0.579) |
| Estimated difference vs. Placebo (95% CI) | -4.225 (-5.674, -2.777) | -5.789 (-7.270, -4.307) | -7.725 (-9.501, -5.948) | -7.949 (-9.816, -6.083) | -7.751 (-9.223, -6.280) |  |
| Analysis was performed using an ANCOVA model, with baseline body weight as a covariate and treatment groups as explanatory variables. Missing body weight data at 16 weeks will be handled using MI based on the MAR assumption. *FAS* full analysis set, *BMI* body mass index, *LSM* least-squares mean, *SE* standard error, *CI* confidence interval, *ANCOVA* analysis of covariance, *MI* multiple imputation, *MAR* missing at random | | | | | | |

**Table S9.** Percent change in body weight from baseline at 16 weeks by baseline BMI strata based on Chinese criteria for overweight subgroup - FAS

| 24 kg/m^2^ ≤ BMI < 28 kg/m^2^ plus ≥ 1 comorbidity | VCT220 80mg  (N=4) | VCT220 120mg  (N=5) | VCT220 160mg-ST  (N=1) | VCT220 160mg-FT  (N=3) | VCT220 160mg-pooled  (N=4) | Placebo  (N=3) |
| --- | --- | --- | --- | --- | --- | --- |
| LSM (SE) of percentage change in body weight | -4.287 (1.139) | -7.957 (1.045) | -11.452 (2.267) | -10.719 (1.348) | -10.902 (1.159) | -1.829 (1.360) |
| 95% CI for LSM of percentage change | (-6.748, -1.827) | (-10.215, -5.698) | (-16.350, -6.553) | (-13.632, -7.806) | (-13.407, -8.398) | (-4.767, 1.109) |
| Estimated difference vs. Placebo (95% CI) | -2.458 (-6.340, 1.423) | -6.128 (-9.713, -2.542) | -9.623 (-15.334, -3.911) | -8.890 (-13.160, -4.620) | -9.073 (-13.041, -5.106) |  |
| Analysis was performed using an ANCOVA model, with baseline body weight as a covariate and treatment groups as explanatory variables. Missing body weight data at 16 weeks will be handled using MI based on the MAR assumption. *FAS* full analysis set, *BMI* body mass index, *LSM* least-squares mean, *SE* standard error, *CI* confidence interval, *ANCOVA* analysis of covariance, *MI* multiple imputation, *MAR* missing at random | | | | | | |

**Table S10.** Percent change in body weight from baseline at 16 weeks by baseline BMI strata based on U.S. criteria for obesity subgroup - FAS

| BMI ≥ 28 kg/m^2^ | VCT220 80mg  (N=39) | VCT220 120mg  (N=40) | VCT220 160mg-ST  (N=22) | VCT220 160mg-FT  (N=22) | VCT220 160mg-pooled  (N=44) | Placebo  (N=46) |
| --- | --- | --- | --- | --- | --- | --- |
| LSM (SE) of percentage change in body weight | -5.718 (0.664) | -7.595 (0.665) | -9.885 (0.900) | -9.694 (0.962) | -9.734 (0.645) | -1.651 (0.615) |
| 95% CI for LSM of percentage change | (-7.020, -4.417) | (-8.899, -6.291) | (-11.648, -8.122) | (-11.578, -7.809) | (-10.997, -8.471) | (-2.856, -0.447) |
| Estimated difference vs. Placebo (95% CI) | -4.067 (-5.840, -2.293) | -5.943 (-7.719, -4.167) | -8.233 (-10.368, -6.099) | -8.042 (-10.278, -5.807) | -8.083 (-9.830, -6.335) |  |
| Analysis was performed using an ANCOVA model, with baseline body weight as a covariate and treatment groups as explanatory variables. Missing body weight data at 16 weeks will be handled using MI based on the MAR assumption. *FAS* full analysis set, *BMI* body mass index, *LSM* least-squares mean, *SE* standard error, *CI* confidence interval, *ANCOVA* analysis of covariance, *MI* multiple imputation, *MAR* missing at random | | | | | | |

**Table S11.** Percent change in body weight from baseline at 16 weeks by baseline BMI strata based on U.S. criteria for overweight subgroup - FAS

| 24 kg/m^2^ ≤ BMI < 28 kg/m^2^ plus ≥ 1 comorbidity | VCT220 80mg  (N=24) | VCT220 120mg  (N=18) | VCT220 160mg-ST  (N=7) | VCT220 160mg-FT  (N=9) | VCT220 160mg-pooled  (N=16) | Placebo  (N=13) |
| --- | --- | --- | --- | --- | --- | --- |
| LSM (SE) of percentage change in body weight | -5.856 (1.265) | -7.390 (1.459) | -7.032 (7.256) | -9.958 (2.024) | -8.635 (1.521) | -1.546 (1.695) |
| 95% CI for LSM of percentage change | (-8.336, -3.376) | (-10.249, -4.531) | (-21.268, 7.203) | (-13.924, -5.991) | (-11.616, -5.654) | (-4.868, 1.777) |
| Estimated difference vs. Placebo (95% CI) | -4.310 (-8.494, -0.126) | -5.844 (-10.205, -1.484) | -5.487 (-19.983, 9.010) | -8.412 (-13.580, -3.244) | -7.089 (-11.555, -2.624) |  |
| Analysis was performed using an ANCOVA model, with baseline body weight as a covariate and treatment groups as explanatory variables. Missing body weight data at 16 weeks will be handled using MI based on the MAR assumption. *FAS* full analysis set, *BMI* body mass index, *LSM* least-squares mean, *SE* standard error, *CI* confidence interval, *ANCOVA* analysis of covariance, *MI* multiple imputation, *MAR* missing at random | | | | | | |
