# Supplementary material for: Oral nonpeptide GLP-1 receptor agonist VCT220 for obesity treatment: a randomized, double-blind, phase II trial
Source: Signal Transduct Target Ther. 2026 Aug 14;11:325. doi: 10.1038/s41392-026-02859-2 (PMC13473623; doi:10.1038/s41392-026-02859-2)
Supplement: Supplementary file 2 [file 41392_2026_2859_MOESM2_ESM.docx]

**A MULTI-CENTER, RANDOMIZED, DOUBLE-BLIND, PLACEBO-CONTROLLED PHASE II CLINICAL STUDY TO EVALUATE THE EFFICACY AND SAFETY OF VCT220 TABLETS IN OVERWEIGHT/OBESE SUBJECTS**

**Study Protocol**

| **Investigational Drug** |  | VCT220 tablets |
| --- | --- | --- |
| **Protocol No.** |  | VCT220-II-02 |
| **Sponsor** |  | Vincentage Pharma Co., Ltd. |
| **Principal Investigator** |  | Ji Linong |
| **Statistical Analysis Unit** |  | R&G PharmaStudies Co., Ltd. |
| **Version No.** |  | 1.0 |
| **Version Date** |  | Oct. 23, 2023 |

**Confidentiality Statement**

All the information contained in this study protocol is proprietary to the sponsor and, therefore, is provided for review only for the investigator, co-investigators, Ethics Committees, regulatory authorities, and other relevant medical institutions. Without the written approval from the sponsor, the information must not be disclosed to any third party not involved in the study, except when it is necessary to explain the study to a potential subject for signing the informed consent form (ICF).

**Protocol Signature Page**

I have read and am familiar with this protocol, confirming that it includes the necessary content for the implementation of the study and clarifying the responsibilities related to this research protocol. I agree to and will strictly adhere to the relevant responsibilities in accordance with Chinese laws and regulations, the Declaration of Helsinki, Good Clinical Practice, and this research protocol.

| Ji Linong |  | Date |
| --- | --- | --- |
| Peking University People's Hospital |  |  |

**Protocol Signature Page**

We have read and are familiar with this protocol, confirming that it includes the necessary content for the implementation of the study and clarifying the responsibilities related to this research protocol. I agree to and will strictly fulfill the relevant responsibilities in accordance with Chinese laws and regulations, the Declaration of Helsinki, Good Clinical Practice, and this research protocol.

| Kong Yunhua |  | Date |
| --- | --- | --- |
| Vincentage Pharma Co., Ltd. |  |  |

**Table of Contents**

[Confidentiality Statement	1](#_Toc2968)

[Protocol Signature Page	2](#_Toc5623)

[Protocol Signature Page	3](#_Toc16273)

[Abstract	13](#_Toc24591)

[Study Flow Chart	26](#_Toc101)

[PK Sampling Schedule	30](#_Toc19682)

[1 Introduction	32](#_Toc1563)

**[1.1 Theoretical Basis of the Study	32](#_Toc18640)**

**[1.2 Overview of Preclinical Studies	34](#_Toc5698)**

**[1.2.1 Pharmacodynamic Studies	34](#_Toc2863)**

**[1.2.2 Pharmacokinetic Studies	34](#_Toc31435)**

**[1.2.3 Toxicology Studies	35](#_Toc20027)**

**[1.3 Overview of Clinical Studies	36](#_Toc1237)**

**[1.4 Risk-Benefit Assessment	41](#_Toc8863)**

[2. Research Objectives	45](#_Toc3323)

[3. Study Design	45](#_Toc14042)

**[3.1 Overall Design	45](#_Toc9214)**

**[3.2 Basis for Research Design	47](#_Toc9002)**

**[3.3 Basis for Dose Selection	47](#_Toc5393)**

**[3.4 Rationale for Endpoint Selection	49](#_Toc26506)**

**[3.5 Randomization and Blinding	50](#_Toc21569)**

**[3.5.1 Randomization	50](#_Toc2982)**

**[3.5.2 Blinding Method	51](#_Toc12040)**

**[3.5.3 Emergency Unblinding	51](#_Toc24995)**

**[3.5.4 Unblinding Regulations	51](#_Toc18238)**

[4. Study Subjects	52](#_Toc25367)

**[4.1 Inclusion Criteria	52](#_Toc6845)**

**[4.2 Exclusion Criteria	52](#_Toc16043)**

**[4.3 Screening Failure	56](#_Toc27891)**

**[4.3.1 Review of Screening Period Examination Items	56](#_Toc25554)**

**[4.3.2 Re-Screening of Subjects	57](#_Toc19008)**

[5. Study Drug	57](#_Toc1344)

**[5.1 Study Drug Information	57](#_Toc23319)**

**[5.2 Study Drug Treatment	57](#_Toc4094)**

**[5.2.1 Dosing Regimen	57](#_Toc11568)**

**[5.2.2 Dose Adjustment Scheme	59](#_Toc17497)**

**[5.3 Drug Management	59](#_Toc2899)**

**[5.3.1 Drug Packaging and Labeling	59](#_Toc18165)**

**[5.3.2 Drug Transportation, Storage, and Distribution	59](#_Toc137)**

**[5.4 Concomitant Treatment	60](#_Toc29646)**

**[5.4.1 Allowed Combination Therapy	60](#_Toc18863)**

**[5.4.2 Prohibited Combination Therapy	61](#_Toc18925)**

**[5.5 Treatment compliance	61](#_Toc20670)**

[6. Stopping Treatment and Participant Withdrawal	62](#_Toc15899)

[7. Research Process	63](#_Toc26443)

**[7.1 Checks and Assessments During the Study Period	63](#_Toc29656)**

**[7.1.1 Informed Consent	63](#_Toc25096)**

**[7.1.2 Collection of Demographic Data	63](#_Toc5985)**

**[7.1.3 Inquiry/Documentation of Medical History and Personal History	63](#_Toc1359)**

**[7.1.4 Previous Treatment History and concomitant reatment	64](#_Toc10377)**

**[7.1.5 Vital Signs and Physical Examination	64](#_Toc29437)**

**[7.1.6 12 Lead Electrocardiogram (ECG)	65](#_Toc17837)**

**[7.1.7 Laboratory Tests	66](#_Toc3108)**

**[7.1.8 Blood Pregnancy Test and Contraceptive Requirements	67](#_Toc16151)**

**[7.1.9 Scales	67](#_Toc3428)**

**[7.1.10 Liver, Gallbladder, Pancreas, Spleen, and Bilateral Kidney Ultrasound	68](#_Toc15997)**

**[7.1.11 FPG, HbA1c, Fasting Insulin, and Fasting C-Peptide	68](#_Toc8933)**

**[7.1.12 Fasting Lipid Profile	68](#_Toc27526)**

**[7.1.13 Height, Weight, and Waist Circumference	68](#_Toc22856)**

**[7.1.14 Collection of Biological Samples	69](#_Toc32128)**

**[7.1.15 Adverse Events	70](#_Toc15120)**

**[7.1.16 Dietary and Exercise Guidance	70](#_Toc27556)**

**[7.2 Visit Schedule	70](#_Toc9803)**

**[7.2.1 Screening Period (D-14 to D-1)	70](#_Toc14494)**

**[7.2.2 Treatment Period	71](#_Toc1154)**

**[7.2.3 Safety Follow-up Period	75](#_Toc31538)**

**[7.2.4 Early Termination Visit	75](#_Toc7997)**

**[7.3 Subject Management	76](#_Toc5432)**

**[7.4 Drug Assay Analysis of Biological Samples	77](#_Toc23970)**

[8 Evaluation Indicators	77](#_Toc21316)

**[8.1 Efficacy Indicators	77](#_Toc23533)**

**[8.2 Safety Indicators	77](#_Toc17073)**

**[8.3 Pharmacokinetic Indicators	78](#_Toc5084)**

[9 Adverse Events and Serious Adverse Events	78](#_Toc14571)

**[9.1Adverse Events	78](#_Toc17727)**

**[9.1.1 Definition of Adverse Events	78](#_Toc7725)**

**[9.1.2 Collection and Recording of Adverse Events	79](#_Toc2129)**

**[9.1.3 Criteria for Determining the Severity of Adverse Events	80](#_Toc24336)**

**[9.1.4 Criteria for Assessing the Relationship Between Adverse Events and Investigational Drugs	81](#_Toc32639)**

**[9.1.5 Outcome of Adverse Events	82](#_Toc17304)**

**[9.1.6 Follow-up of Adverse Events	82](#_Toc3276)**

**[9.2 Serious Adverse Events	83](#_Toc8099)**

**[9.2.1 Definition of Serious Adverse Events	83](#_Toc20213)**

**[9.2.2 Reporting and Handling of Serious Adverse Events	85](#_Toc12267)**

**[9.3 Suspected Unexpected Serious Adverse Reactions (SUSAR)	86](#_Toc3320)**

**[9.3.1 Definition of SUSAR	86](#_Toc20470)**

**[9.3.2 Reporting of SUSAR	86](#_Toc10938)**

**[9.4 Pregnancy	86](#_Toc22698)**

[10 Data Management	87](#_Toc22481)

**[10.1 Database Establishment	87](#_Toc13411)**

**[10.2 Database Verification	87](#_Toc29130)**

**[10.3 Database Locking	87](#_Toc23729)**

[11 Statistical Analysis and Sample Size	88](#_Toc7513)

**[11.1 Sample Size Calculation	88](#_Toc22325)**

**[11.2 Statistical Analysis Data Sets	88](#_Toc30297)**

**[11.3 Statistical Analysis Methods	89](#_Toc12849)**

**[11.3.1 General Principles of Statistical Analysis	89](#_Toc29836)**

**[11.3.2 Demographic and Baseline Characteristics Analysis	89](#_Toc18118)**

**[11.3.3 Efficacy Analysis	89](#_Toc21355)**

**[11.3.4 Safety Analysis	90](#_Toc30198)**

**[11.3.5 Pharmacokinetic Analysis	91](#_Toc32149)**

**[11.3.6 Population Pharmacokinetic Analysis and Exposure-Response Analysis	91](#_Toc32484)**

[12 Research Management	91](#_Toc16543)

**[12.1 Ethics	91](#_Toc16363)**

**[12.1.1 Ethical Requirements	91](#_Toc29762)**

**[12.1.2 Informed Consent	92](#_Toc377)**

**[12.1.3 Confidentiality of Subjects	93](#_Toc22603)**

**[12.2 Responsibilities of All Parties	93](#_Toc8415)**

**[12.2.1 Responsibilities of investigator	93](#_Toc18102)**

**[12.2.2 Responsibilities of the Sponsor	95](#_Toc25195)**

**[12.3 Protocol Amendments	96](#_Toc974)**

**[12.4 Training	96](#_Toc11657)**

**[12.5 Standard Operating Procedures	96](#_Toc1118)**

**[12.6 Quality Control and Assurance	97](#_Toc19138)**

**[12.6.1 Monitoring	97](#_Toc546)**

**[12.6.2 Auditing and Inspection	99](#_Toc15050)**

**[12.7 Early Termination or Suspension of the Study	99](#_Toc14630)**

**[12.8 Record Keeping and Retention of Study Data	101](#_Toc14089)**

**[12.9 Confidentiality and Publication of Research Results	101](#_Toc15537)**

[13 References	101](#_Toc12083)

[14 Appendix	104](#_Toc2284)

[Appendix 1: Definition of Comorbidities	104](#_Toc29942)

[Appendix 2: Instructions for Measuring Height, Weight, and Waist Circumference	105](#_Toc24170)

[Appendix 3: Patient Health Questionnaire-9 (PHQ-9)	107](#_Toc3527)

[Appendix 4: Hypoglycemia Diagnosis and Treatment Process	109](#_Toc7988)

[Appendix 5: BMI Calculation Formula	110](#_Toc9293)

[Appendix 6: QTcF Calculation Formula	111](#_Toc24851)

[Appendix 7: Glomerular Filtration Rate Calculation Formula	112](#_Toc7682)

[Appendix 8: Dietary and Exercise Guidance	113](#_Toc18206)

**Abbreviations**

| **English Abbreviations** | **Chinese Name** |
| --- | --- |
| AE | Adverse Events |
| ALB | Albumin |
| ALP | Alkaline Phosphatase |
| ALT | Alanine Aminotransferase |
| ANCOVA | Analysis of Covariance |
| APTT | Activated Partial Thromboplastin Time |
| AST | Aspartate Aminotransferase |
| AUC | Drug-Time Area Under the Curve |
| AUC_0-inf_ | Area Under the Curve from 0 h to Infinity |
| AUC0-last | Area Under the Curve from 0 h to Last Quantifiable Concentrati |
| AUC_0-tau_ | Drug-Time Area Under the Curve within Dosing Interval |
| ADL | Activities of Daily Living |
| BASO | Eosinophils |
| BIL | Urobilinogen |
| BLD | Urine Occult Blood |
| BMI | Body Mass Index |
| BUN | Blood Urea Nitrogen |
| CK | Creatine Kinase |
| CK-MB | Creatine Kinase Isoenzyme |
| CL/F | Apparent Clearance Rate |
| C_max_ | Peak Concentration |
| C_min_ | Trough Concentration |
| Cr | Creatinine |
| CTCAE | Common Terminology Criteria for Adverse Events |
| DBIL | Direct Bilirubin |
| DPP-4 | Dipeptidyl Peptidase-4 |
| 12-ECG | 12-Lead Electrocardiogram |
| eCRF | Electronic Case Report Form |
| EDC | Electronic Data Capture System |
| eGFR | Glomerular Filtration Rate |
| EO | Eosinophils |
| FAS | Full Analysis Set |
| FPG | Fasting Plasma Glucose |
| FT_4_ | Free Thyroxine |
| FT_3_ | Free Triiodothyronine |
| FDA | U.S. Food and Drug Administration |
| GCP | Good Clinical Practice |
| GGT | Gamma-Glutamyl Transferase |
| GLO | Globulin |
| GLP-1 | Glucagon-Like Peptide-1 |
| GLP-1 RA | Glucagon-Like Peptide-1 Receptor Agonist |
| GLP-1R | Glucagon-Like Peptide-1 Receptor |
| GIP | Glucose-Dependent Insulinotropic Polypeptide |
| GCGR | Glucagon Receptor |
| GLU | Urine Glucose |
| HbA_1c_ | Glycated Hemoglobin |
| HBsAg | Hepatitis B Virus Surface Antigen |
| HBV-DNA | Hepatitis B Virus Deoxyribonucleic Acid |
| HCT | Hematocrit |
| HCV | Hepatitis C Virus |
| HDL-C | High-Density Lipoprotein Cholesterol |
| Hb | Hemoglobin |
| HIV | Human Immunodeficiency Virus |
| INR | International Normalized Ratio |
| IWRS | Central Randomization System |
| KET | Urine Ketones |
| LDH | Lactate Dehydrogenase |
| LDL-C | Low-Density Lipoprotein Cholesterol |
| LYMPH | Lymphocytes |
| MedDRA | International Medical Terminology Dictionary |
| MEN | Multiple Endocrine Neoplasia |
| MONO | Monocytes |
| MRT | Mean Residence Time |
| MTD | Maximum Tolerated Dose |
| NEUT | Neutrophils |
| NMPA | National Medical Products Administration |
| NOAEL | No Observable Adverse Effect Level |
| NONMEM | Nonlinear Mixed-Effects Model |
| NYHA | New York Heart Association |
| PHQ-9 | Depression Screening Scale |
| PK | Pharmacokinetics |
| PKCS | Pharmacokinetic Concentration Set |
| PKPS | Pharmacokinetic Parameter Set |
| PLT | Platelet Count |
| PopPK | Population Pharmacokinetics |
| PRO | Urine Protein |
| PT | Prothrombin Time |
| PT | Preferred Terms |
| QD | Once Daily |
| RBC | Red Blood Cell Count |
| SAE | Serious Adverse Events |
| SAP | Statistical Analysis Plan |
| SG | Urine Specific Gravity |
| SGLT-2 | Sodium-Glucose Cotransporter-2 |
| SOC | System Organ Class |
| SOP | Standard Operating Procedures |
| SS | Safety Analysis Set |
| SUSAR | Suspected Unexpected Serious Adverse Reactions |
| t_1/2_ | Terminal Half-Life |
| T2DM | Type 2 Diabetes Mellitus |
| TBIL | Total Bilirubin |
| TC | Total Cholesterol |
| TEAE | Adverse Events During Treatment Period |
| TG | Triglycerides |
| T_max_ | Time to Peak |
| TP | Total Protein |
| Tp-Ab | Treponema Antibodies |
| TSH | Thyroid-Stimulating Hormone |
| TZD | Thiazolidinediones |
| UA | Uric Acid |
| URO | Urobilinogen |
| ULN | Upper Limit of Normal Values |
| V_z_/F | Apparent Volume of Distribution |
| WBC | White Blood Cell Count |

Abstract

| **Study title** | A Multi-center, randomized, double-blind, placebo-controlled phase II clinical study to evaluate the efficacy and safety of VCT220 tablets in overweight/obese subjects |
| --- | --- |
| **Investigational drug** | VCT220 tablets |
| **Protocol No.** | VCT220-II-02 |
| **Sponsor** | Vincentage Pharma Co., Ltd. |
| **Study phase** | Phase II |
| **Leading site** | Peking University People's Hospital |
| **Principal investigator** | Ji Linong |
| **Study population** | Overweight/obese subjects without diabetes mellitus |
| **Study objectives** | **Primary objective:**   - To assess the effect of VCT220 tablets at different doses on weight loss in non-diabetic overweight/obese subjects.   **Secondary objectives:**   - To assess the safety of VCT220 tablets at different doses in non-diabetic overweight/obese subjects. - To assess the pharmacokinetic (PK) characteristics of VCT220 tablets at different doses in non-diabetic overweight/obese subjects.   **Exploratory objective:**   - To preliminarily explore the relationship between the exposure of VCT220 tablets and the efficacy and adverse events (AEs) in non-diabetic overweight/obese subjects. |
| **Study design** | **Overall design:** This is a multi-center, randomized, double-blind, placebo-controlled phase II clinical study to evaluate the efficacy, safety, and PK characteristics of VCT220 tablets at different doses in non-diabetic overweight/obese subjects. The study consists of 3 study periods, namely, a screening period (up to 2 weeks), a treatment period (16 weeks), and a safety follow-up period (2 weeks). A total of 240 subjects who meet the eligibility criteria for this study will be randomly assigned in a 3:1 ratio to the investigational drug group or the placebo group to receive treatment with VCT220 tablets or placebo. The grouping and number of subjects in each group for this study are detailed as follows: 80 mg dose group (N = 60), 80 mg dose-matched placebo group (N = 20), 120 mg dose group (N = 60), 120 mg dose-matched placebo group (N = 20), 160 mg fast titration group (N = 30), 160 mg dose-matched fast titration placebo group (N = 10), 160 mg slow titration group (N = 30), and 160 mg dose-matched slow titration placebo group (N = 10). Subjects will be enrolled into each dose group in parallel. A total of 180 subjects will be treated with VCT220 tablets and 60 subjects will be treated with placebo. The subjects will be given VCT220 tablets or placebo at corresponding doses after randomization, which will be orally administered with breakfast once daily for 16 consecutive weeks. All subjects will enter a 2-week safety follow-up period after they have completed the 16-week treatment period. PK blood sampling will be performed for all subjects throughout the study treatment period. All subjects should undergo sparse PK blood sampling at the time points planned in the protocol, and approximately 40 of these subjects will also undergo intensive PK blood sampling at scheduled time points in the protocol, including 10 subjects from the 80 mg dose group (or its placebo group), 10 subjects from the 120 mg dose group (or its placebo group), 10 subjects from the 160 mg slow titration group (or its placebo group), and 10 subjects from the 160 mg fast titration group (or its placebo group). During the study, the investigator should instruct the subjects on medication compliance and individualized diet and exercise, and the subjects are requested to comply with the basic diet and exercise requirements throughout the study. For each dose group (and its corresponding placebo group), titration will be performed during the treatment period as follows: \| **W1** \| **W2** \| **W3** \| **W4** \| **W5** \| **W6** \| **W7-W16**; 80 mg dose group \| 20 mg \| 40 mg \| 80 mg \| 80 mg \| 80 mg \| 80 mg \| 80 mg; 120 mg dose group \| 20 mg \| 40 mg \| 60 mg \| 80 mg \| 120 mg \| 120 mg \| 120 mg; 160 mg fast titration group \| 20 mg \| 40 mg \| 80 mg \| 120 mg \| 160 mg \| 160 mg \| 160 mg; 160 mg slow titration group \| 20 mg \| 20 mg \| 40 mg \| 40 mg \| 80 mg \| 120 mg \| 160 mg |
| **Selection of subjects** | **Inclusion criteria**   1) Male or female subjects aged 18–75 years (inclusive) when signing the informed consent form; 2) Body mass index (BMI) ≥ 28 kg/m2 with or without concomitant diseases; or BMI ≥ 24 kg/m2 and < 28 kg/m2 combined with at least one of the following concomitant diseases: pre-diabetes (impaired fasting glucose and/or reduced glucose tolerance), hypertension, dyslipidemia, fatty liver, and obstructive sleep apnea syndrome caused by overweight (see Appendix 1 for specific definitions); 3) Subjects who have undergone diet control or exercise control alone for at least 12 weeks before screening, resulting in a stable weight within 12 weeks before screening (weight change < 5%, weight change = [maximum weight − minimum weight]/maximum weight; based on subjects' self-report); 4) Subjects who have no fertility plan and voluntarily take effective contraceptive measures without any plans to donate sperm or eggs during the study and within 90 days after the end of study; 5) Subjects who are willing and able to maintain a stable diet and exercise during the study and fill in diary cards on time; 6) Subjects who are able to understand the procedures and methods of this study, willing to complete this study in strict accordance with the clinical study protocol, and voluntarily sign the informed consent form. |
|  | **Exclusion criteria**   1) Subjects with previously diagnosed obesity caused by endocrine diseases or single gene mutations, including but not limited to hypothalamic obesity, pituitary obesity, hypothyroid obesity, Cushing's syndrome, islet cell tumor, acromegaly, and hypogonadism; 2) Subjects who have received bariatric surgery or treatment (except for acupuncture for weight loss, liposuction, and abdominal fat removal more than 1 year from the screening period), or plan to receive bariatric surgery to treat obesity during the study, e.g., gastric bypass surgery and gastric banding; 3) Subjects who are on a weight loss program at screening and are not in the maintenance period (based on subjects' self-report); 4) Subjects who have previously been diagnosed with type 1 diabetes mellitus, type 2 diabetes mellitus, or other special types of diabetes; 5) Subjects who have used any GLP-1 receptor agonist (GLP-1RA), GLP-1 related multi-target agonist (e.g., GLP‑1/glucose-dependent insulinotropic polypeptide [GIP] dual agonist and GLP‑1/glucagon receptor [GCGR] dual agonist), or compound preparations containing GLP‑1 agonist; 6) Subjects who have received any of the following medications or therapies within 12 weeks before screening:  - Oral or injectable hypoglycemic medications such as dipeptidyl peptidase-4 (DPP-4) inhibitors, sodium glucose cotransporter 2 (SGLT-2) inhibitors, metformin, insulin secretagogues, thiazolidinediones (TZDs), and insulin; - Any approved or unapproved weight loss drugs (such as orlistat, lorcaserin, phentermine/topiramate, and naltrexone/bupropion) or Chinese herbal medicines, health care products, meal replacements, etc., affecting body weight; - Use of any drugs that might significantly change body weights, including systemic (i.e., intravenous, oral, or intra-articular administration) glucocorticoids for more than 1 week; tricyclic antidepressants; antipsychotic or antiepileptic drugs (e.g., imipramine, amitriptyline, mirtazapine, paroxetine, phenelzine, chlorpromazine hydrochloride, clozapine, olanzapine, valeric acid and its derivatives, lithium preparations, and thioridazine), etc.;  7) Subjects who meet any of the following criteria related to cardiac function:  - History of myocardial infarction, coronary angioplasty or bypass graft, heart valve disorders or cardiac valve prostheses, clinically significant unstable arrhythmia, unstable angina, transient ischemic attack, cerebrovascular accident, etc., within 6 months before screening; - Class III or IV congestive cardiac failure according to New York Heart Association (NYHA) classification; - Subjects with serious arrhythmia requiring treatment (such as long QT syndrome) and not suitable for the clinical study as assessed by the investigator; - Poorly controlled hypertension before screening (i.e., systolic blood pressure ≥ 160 mmHg or diastolic blood pressure ≥ 100 mmHg); - Presence of clinical abnormalities (e.g., QTcF > 450 msec, complete left bundle branch block, signs of acute or unexplained myocardial infarction, ST-segment changes suggestive of myocardial ischemia, second or third degree atrioventricular block, or serious bradyarrhythmia or tachyarrhythmia) by electrocardiography (ECG) at screening that might affect the safety of subjects or the interpretation of study results; - Resting heart rate > 100 beats/min; - Other cardiac function abnormalities with clinical significance judged by the investigator;  8) Currently with thyroid dysfunction that is not well controlled with a stable dose of medications, or with clinically significant abnormalities noted in thyroid function test results at screening; 9) History of medullary thyroid cancer, multiple endocrine neoplasia (MEN) syndrome type 2A or 2B or relevant family history (family history is defined as diseases in a first-degree relatives); 10) Subjects diagnosed with malignancies within 5 years before screening (except for cured cutaneous basal cell carcinoma or cervical carcinoma *in situ*); 11) History of acute or chronic pancreatitis, symptomatic gallbladder disease (such as multiple gallstones), pancreatic injury, and other high-risk factors that may lead to pancreatitis; 12) Subjects with clinically significant abnormal gastric emptying (such as gastric outlet obstruction), severe chronic gastrointestinal diseases (active ulcer within 6 months), long-term use of drugs with direct effects on gastrointestinal motility (including but not limited to mosapride, cisapride, etc.), or gastrointestinal surgery (except for polypectomy and appendectomy) within 6 months before screening, who are not suitable for the clinical study as assessed by the investigator; 13) History of organ transplantation, or previous or current serious autoimmune diseases; 14) Subjects who have a history of major surgery within 6 months before screening, or plan to undergo surgery that might affect study completion or compliance; 15) Subjects with a history of moderate to severe depression and anxiety, or a history of serious mental illness, or a depression screening scale (PHQ-9) score ≥ 15 at screening; 16) Subjects with previous suicidal tendency or suicidal behavior; 17) Subjects with any laboratory test parameter meeting any of the following criteria at screening:  - Glycosylated hemoglobin (HbA_1c_) ≥ 6.5%; - Fasting plasma glucose (FPG) ≥ 7.0 mmol/L; - Glomerular filtration rate (eGFR) < 60 mL/min/1.73 m^2^ as calculated using the CKD-EPI formula; - Alanine aminotransferase (ALT) and/or aspartate aminotransferase (AST) ≥ 3.0 × upper limit of normal (ULN); - Total bilirubin (TBIL) > 1.5 × ULN; - Blood amylase or blood lipase > 1.5 × ULN; - Fasting triglycerides (TG) > 5.7 mmol/L; - Fasting C-peptide < 0.8 ng/mL (or < 0.26 nmol/L); - Calcitonin ≥ 1.0 × ULN; - Hemoglobin (Hb) < 110 g/L (males) or < 100 g/L (females);  18) Subjects who are positive for hepatitis B virus surface antigen (HBsAg) (except for those with hepatitis B virus deoxyribonucleic acid [HBV-DNA] quantitative results lower than the lower limit of reference range and not using anti-hepatitis B virus drugs at screening), receiving anti-hepatitis B virus drugs, positive for hepatitis C virus (HCV) antibody, positive for human immunodeficiency virus (HIV) antibody, or positive for treponema pallidum antibody (Tp-Ab) at screening; 19) Subjects with known allergies to GLP-1 receptor agonists or relevant excipients; 20) Subjects who have previously participated in weight loss clinical trials; those who have participated in any clinical trial for drug or medical device (except for weight loss clinical trials) as subjects within 12 weeks before screening, or subjects who have participated in any clinical trials in which the last dose is within 5 half-lives of the investigational product before screening (whichever is longer) (such restrictions are not applicable to the subjects who fail the screening); 21) Subjects who have donated or lost ≥ 400 mL of blood or have received blood transfusion within 12 weeks before screening; 22) Subjects with a known or suspected history of alcohol or tobacco addiction, or drug abuse at screening; 23) Pregnant or lactating women; 24) Subjects with positive blood pregnancy test results at screening or before randomization (for female subjects of childbearing potential only); 25) Other conditions that make the subject unsuitable for the study as judged by the investigator. |
| **Investigational products** | - **Investigational drug:** VCT220   Name: VCT220 tablets  Strength: tablets, 10 mg/tablet, 50 mg/tablet   - **Control drug:** placebo   Name: VCT220 dummy tablets  Strength: tablet  The placebo does not differ from VCT220 tablets in appearance (shape, color, size, and packaging) and odor and does not contain active ingredients. |
| **Study**  **treatment** | Dosage and administration: The tablets will be administered orally once daily with meals and it is recommended to take the tablets with breakfast. The number of tablets and the dosage to be taken in each dose group during the treatment period are as follows: - 80 mg dose group (or its placebo group) Treatment cycle \| Total amount per dose \| Method of administration each time \| \| Total number of tablets each time; \| \| 10 mg/tablet \| 50 mg/tablet \|; W1 \| 20 mg \| 2 \| 0 \| 2; W2 \| 40 mg \| 4 \| 0 \| 4; W3-W16 \| 80 mg \| 3 \| 1 \| 4 - 120 mg dose group (or its placebo group) Treatment cycle \| Total amount per dose \| Method of administration each time \| \| Total number of tablets each time; \| \| 10 mg/tablet \| 50 mg/tablet \|; W1 \| 20 mg \| 2 \| 0 \| 2; W2 \| 40 mg \| 4 \| 0 \| 4; W3 \| 60 mg \| 1 \| 1 \| 2; W4 \| 80 mg \| 3 \| 1 \| 4; W5-W16 \| 120 mg \| 2 \| 2 \| 4 - 160 mg fast titration group (or its placebo group) Treatment cycle \| Total amount per dose \| Method of administration each time \| \| Total number of tablets each time; \| \| 10 mg/tablet \| 50 mg/tablet \|; W1 \| 20 mg \| 2 \| 0 \| 2; W2 \| 40 mg \| 4 \| 0 \| 4; W3 \| 80 mg \| 3 \| 1 \| 4; W4 \| 120 mg \| 2 \| 2 \| 4; W5-W16 \| 160 mg \| 1 \| 3 \| 4 - 160 mg slow titration group (or its placebo group) Treatment cycle \| Total amount per dose \| Method of administration each time \| \| Total number of tablets each time; \| \| 10 mg/tablet \| 50 mg/tablet \|; W1-W2 \| 20 mg \| 2 \| 0 \| 2; W3-W4 \| 40 mg \| 4 \| 0 \| 4; W5 \| 80 mg \| 3 \| 1 \| 4; W6 \| 120 mg \| 2 \| 2 \| 4; W7-W16 \| 160 mg \| 1 \| 3 \| 4 |
| **Concomitant therapy** | - **Prohibited concomitant treatments:** - Any approved or unapproved weight loss drugs (such as liraglutide, semaglutide, beinaglutide, orlistat, lorcaserin, phentermine/topiramate, and naltrexone/bupropion) or Chinese herbal medicines, health care products, meal replacements, etc., affecting body weight; - Any hypoglycemic medications (e.g., GLP-1 single- or multi-target agonists, GLP-1RA-containing compound preparations, DPP-4 inhibitors, SGLT‑2 inhibitors, insulin, and metformin); - Any treatment that might lead to weight instability, including weight loss drugs and weight gain drugs, such as systemic glucocorticoids lasting for more than 1 week; tricyclic antidepressants; antipsychotic or antiepileptic drugs (e.g., imipramine, amitriptyline, mirtazapine, paroxetine, phenelzine, chlorpromazine hydrochloride, clozapine, olanzapine, valeric acid and its derivatives, lithium preparations, and thioridazine), etc.; - Bariatric surgery/non-drug therapies: sleeve gastrectomy for weight loss, liposuction, etc.; TCM weight loss therapy (acupuncture, moxibustion, naprapathy, cupping, etc.). - **Permitted concomitant treatments** - During the study, the pre-existing antihypertensives and lipid-regulating drugs of the subjects can be continued, but the varieties and doses of the pre-existing drugs should be kept unchanged as far as possible. The medication regimen can be adjusted during the study only when adequate supportive treatments are required as considered by the investigator; - In case of AEs requiring treatment, subjects may be given drugs for symptomatic treatment (drugs/treatments that are not expected to interfere with the assessment of efficacy and safety of the investigational drug or have no interaction with the investigational drug) during the study; - Medications used to treat underlying diseases (other than those prohibited for concomitant therapy). |
| **Evaluation endpoints** | **Efficacy endpoints**   - **Primary efficacy endpoint:** - Percentage change from baseline in body weight after 16 weeks of treatment. - **Secondary efficacy endpoints:** - Proportion of subjects with weight decrease ≥ 5% from baseline after 16 weeks of treatment; - Absolute change from baseline in body weight after 16 weeks of treatment; - Absolute change from baseline in BMI after 16 weeks of treatment; - Absolute change from baseline in waist circumference after 16 weeks of treatment; - Change from baseline in blood lipid profiles (TC, TG, LDL-C, and HDL‑C) after 16 weeks of treatment; - Change from baseline in blood glucose levels (FPG, HbA_1c_, fasting insulin, fasting C-peptide) after 16 weeks of treatment;   **Safety endpoints**   - Adverse events (AEs), including treatment-emergent adverse events (TEAEs), serious adverse events (SAEs), etc.; - Vital signs (sitting blood pressure [systolic and diastolic blood pressure], pulse rate, and body temperature), physical examination, and 12-lead ECG; - Clinical laboratory tests (hematology, urinalysis, blood chemistry, blood lipase, blood amylase, thyroid function, coagulation function, and calcitonin);   **PK endpoints**   - PK analysis: maximum concentration (C_max_), minimum concentration (C_min_), time to maximum concentration (T_max_), area under the concentration-time curve from 0 h to the time of the last quantifiable concentration (AUC_0-last_), and area under the concentration-time curve over the dosing interval (AUC_0-tau_); if data permit, the area under the concentration-time curve from 0 h to infinity (AUC_0-inf_), apparent clearance (CL/F), apparent volume of distribution (V_z_/F), terminal elimination half-life (t_1/2_), etc., will also be calculated. - Population pharmacokinetic (PopPK) analysis: To explore the PK characteristics of VCT220 tablets in non-diabetic overweight/obese subjects based on the PopPK analysis method. |
| **Statistical analysis**  **and sample size** | **Sample size calculation**  No formal statistical hypothesis test is planned in this study. A total of 240 subjects will be randomized in a 3:1 ratio to the investigational drug group or the placebo group. The grouping and number of subjects in each group for this study are detailed as follows: 80 mg dose group (N = 60), 80 mg dose-matched placebo group (N = 20), 120 mg dose group (N = 60), 120 mg dose-matched placebo group (N = 20), 160 mg fast titration group (N = 30), 160 mg dose-matched fast titration placebo group (N = 10), 160 mg slow titration group (N = 30), and 160 mg dose-matched slow titration placebo group (N = 10). A total of 180 subjects will be treated with VCT220 tablets and 60 subjects will be treated with placebo during the study.  **Statistical analysis sets**  Full analysis set (FAS): It is defined as all subjects who are randomized and have received at least one dose of the investigational product. The FAS population will serve as the primary population for efficacy analysis in this study.  Safety set (SS): It is defined as all subjects who have received at least one dose of the investigational product. The safety analysis population is the primary population for safety endpoint analysis that will be performed based on actual dose groups.  Pharmacokinetic concentration set (PKCS): It is defined as all randomized subjects who have received at least one dose of the investigational product and have at least one evaluable concentration datum after drug administration.  Pharmacokinetic parameter set (PKPS): It is defined as all randomized subjects who have received at least one dose of the investigational product and have at least one evaluable PK parameter after drug administration.  **Statistical analysis methods**   - **Efficacy analysis**   The primary efficacy endpoints in this study will be analyzed based on the FAS using the analysis of covariance (ANCOVA) as the primary analysis method. The model is used to investigate the residual variances of different dose groups with treatment as a factor and baseline body weight as a covariate. The primary analysis results are point estimates and 95% CIs of inter-group differences (treatment group vs. control group) in changes from baseline of all dose groups.  The secondary endpoints will be analyzed based on the FAS. For continuous variables, an ANCOVA model will be constructed with the dose group as the effect and baseline body weight as the covariate, and the least squares estimated difference between the treatment group and the placebo group and its 95% CI will be calculated. For categorical variables, the Newcombe hybrid score for CI calculation will be used to calculate the ratio differences between groups and their 95% CIs.  The efficacy analysis in this study will be summarized by the 80 mg dose group, the 120 mg dose group, and the 160 dose groups (including both the 160 mg fast titration and slow titration groups).   - **Safety analysis**   All AEs will be grouped and summarized according to the system organ class (SOC) and preferred term (PT) stipulated in the latest version of Medical Dictionary for Regulatory Activities (MedDRA) or updated Chinese version based on the SS. If a subject experiences more than one AE, the subject will be counted as 1 during the calculation of the incidence; if a subject consecutively experiences the same event multiple times, the event will be counted as 1 case according to the worst severity.  The number of subjects, the number of events, and percentage of TEAEs will be summarized by SOC and PT, and TEAEs will be summarized by the causality with the investigational product and Common Terminology Criteria for Adverse Events (CTCAE) grade. The investigational product-related TEAEs, SAEs, TEAEs leading to treatment discontinuation, and TEAEs leading to death will be separately summarized, and the details of all AEs will be tabulated (e.g., AE name, start time, end time, outcome, severity, causality with the investigational product, therapeutic measures taken for AEs, and actions taken for the investigational product due to AEs).  Based on the SS, a cross-table will be formulated using the physical examinations, 12 lead-ECG, and laboratory tests indicators as well as corresponding results at baseline, and the number of subjects and percentage will be recorded, respectively. The data of vital signs (blood pressure, pulse, and body temperature) will be statistically described, including the values at each time point and their changes from baseline.  The safety analysis of this study will be summarized by 80 mg dose group, 120 mg dose group, 160 mg fast titration group, and 160 mg slow titration group.   - **PK analysis**   The concentration data will be descriptively summarized by PK sampling time points and doses based on the PKCS. Individual subject blood concentration-time curves and corresponding summary curves will be plotted by dose using linear and semi-logarithmic scales. Scheduled PK sampling time points will be used to calculate summary statistics and plot summary curves (means) as per sampling time; actual PK sampling time points will be used to plot individual subject curves by time.  For each dose group, PK parameters will be summarized descriptively: C_max_, C_min_, T_max_, AUC_0‑last_, and AUC_0-tau_; if data permit, AUC_0-inf_, t_1/2_, CL/F, V_z_/F, etc., will also be calculated.  The linear relationship between primary exposure parameters (C_max_ and AUC_0-tau_) and dose will be investigated using a power function model and the dose normalization method, as data permitted.   - **PopPK analysis and analysis of dose-response relationship**   All blood concentrations and other data obtained in this study will be combined with other available clinical study data of VCT220 tablets to establish a PopPK model (using nonlinear mixed effects model [NONMEM]) to characterize the PK profiles of VCT220. Further exposure-response analysis will be performed, including exploratory analysis of exposure-efficacy and exposure‑safety correlations. The results of these analyses will be reported separately. Specific methods and contents of the PopPK model and exposure-response model analyses will be detailed in the pharmacometrics analysis plan. |

Study Flow Chart

**Time (week)**

**Placebo**

**Placebo**

**Placebo**

**Placebo**

**Treatment period (16 weeks)**

**Screening**

**(≤ 2 weeks)**

**Safety**

**follow-up period**

**(2 weeks)**

**VCT220 80 mg group**

**VCT220 120 mg group**

**VCT220 160 mg**

**fast titration group**

**VCT220 160 mg**

**slow titration group**

| **Item/study cycle** | **Screening** | **Treatment period** | | | | | | | **Safety follow-up period** | **Early withdrawal** |
| --- | --- | --- | --- | --- | --- | --- | --- | --- | --- | --- |
| **Visit**^&^ | **V0** | **V1** | **V2** | **V3** | **V4** | **V5** | **V6** | **V7** | **V8** |  |
| **Visit time point (week)** | **W−2 to W−1** | **W1**# | **W3** | **W5** | **W7** | **W9** | **W13** | **W17** | **W19** |  |
| **Time (day)** | **D−14 to D−1** | **D1** | **D15** | **D29** | **D43** | **D57** | **D85** | **D113** | **D126** |  |
| **Time window (day)** | **-** | **-** | **± 3** | **± 3** | **± 3** | **± 3** | **± 3** | **+3** | **± 7** |  |
| **Study procedures** |  |  |  |  |  |  |  |  |  |  |
| Informed consent | X |  |  |  |  |  |  |  |  |  |
| Collection of demographic data^1^ | X |  |  |  |  |  |  |  |  |  |
| Inquiring and recording medical history^2^ | X |  |  |  |  |  |  |  |  |  |
| Prior treatment history^3^ | X |  |  |  |  |  |  |  |  |  |
| Confirming inclusion/exclusion criteria | X | X |  |  |  |  |  |  |  |  |
| Randomization^4^ |  | X |  |  |  |  |  |  |  |  |
| **Safety clinical operations/assessments** |  |  |  |  |  |  |  |  |  |  |
| Vital signs^5^ | X | X | X | X | X | X | X | X | X | X |
| Complete physical examination^6^ | X | X |  |  |  |  |  | X | X | X |
| Symptom-directed physical examination^7^ |  |  | X | X | X | X | X |  |  |  |
| 12-lead ECG^8^ | X | X | X | X | X | X | X | X | X | X |
| Hematology^9^ | X | X | X | X | X | X | X | X | X | X |
| Blood chemistry^10^ | X | X | X | X | X | X | X | X | X | X |
| Urinalysis^11^ | X | X | X | X | X | X | X | X | X | X |
| Blood amylase and blood lipase | X | X | X | X | X | X | X | X | X | X |
| Blood pregnancy test^12^ | X | X |  | X |  |  |  | X | X | X |
| Thyroid function^13^ | X |  |  | X |  |  |  | X | X | X |
| Coagulation function^14^ | X |  |  | X |  |  |  | X | X | X |
| Calcitonin | X |  |  | X |  |  |  | X | X | X |
| Four items of infection^15^ | X |  |  |  |  |  |  |  |  |  |
| PHQ-9 | X |  |  |  |  |  |  |  |  |  |
| B-mode ultrasound in liver, gallbladder, pancreas, spleen, and bilateral kidneys^16^ | X |  |  |  |  |  |  | X | X | X |
| **Efficacy clinical operations/assessments** |  |  |  |  |  |  |  |  |  |  |
| Body height measurement^17^ | X |  |  |  |  |  |  |  |  |  |
| Fasting body weight measurement^18^ | X | X | X | X | X | X | X | X | X | X |
| Fasting waist circumference^19^ | X | X | X | X | X | X | X | X | X | X |
| FPG* | X | X* | X* | X* | X* | X* | X* | X* | X | X* |
| HbA_1c_* | X | X* |  | X* |  |  |  | X* |  | X* |
| Fasting insulin* |  | X* |  | X* |  |  |  | X* |  | X* |
| Fasting C-peptide* | X | X* |  | X* |  |  |  | X* |  | X* |
| Fasting blood lipids^20^* | X | X* |  | X* |  |  |  | X* |  | X* |
| **Biological sample collection** |  |  |  |  |  |  |  |  |  |  |
| Sparse PK blood sampling^21^* |  | X | X | X | X | X | X |  |  | X |
| Intensive PK blood sampling^22^* |  | X | X | X | X | X | X |  |  | X |
| **Medication/diary card** |  |  |  |  |  |  |  |  |  |  |
| Drug dispensing |  | X | X | X | X | X | X |  |  |  |
| Drug return |  |  | X | X | X | X | X | X |  | X |
| Diary card dispensing^23^ |  | X | X | X | X | X | X |  |  |  |
| Diary card return^23^ |  |  | X | X | X | X | X | X |  | X |
| **Others** |  |  |  |  |  |  |  |  |  |  |
| Adverse event |  | X | X | X | X | X | X | X | X | X |
| Concomitant therapy | X | X | X | X | X | X | X | X | X | X |
| Diet and exercise instruction^24^ | X | X | X | X | X | X | X | X | X | X |

Notes:

& Subjects should undergo all routinely scheduled visits under a fasting state (fasted for at least 8 h).

***** FPG, HbA_1c_, fasting insulin, fasting C-peptide, fasting blood lipids, sparse PK blood samples, and intensive PK blood samples at visits during the treatment period and at the early withdrawal visit will be uniformly tested by the central laboratory. Other items not marked with *, including FPG, HbA_1c_, fasting C-peptide, and fasting blood lipids during the screening period, all safety tests (hematology, urinalysis, blood chemistry, blood lipase, blood amylase, blood pregnancy test, thyroid function, coagulation function, calcitonin, and four items of infection) conducted during the study period and FPG during the safety follow-up period, will be tested by a local laboratory of each study site. In case of any test item that cannot be examined by the local laboratory, samples can be sent to the central laboratory for testing.

\# If the 12-lead ECG, hematology, urinalysis, blood chemistry, blood lipase, and blood amylase of a subject in the screening period are examined within 7 days from D1, the above examinations may not be repeated on D1, and the results obtained during the screening period will be used as baseline values. For abnormal results during the screening period, corresponding tests will be repeated and the repeat test results will be used as the baseline of this study; blood pregnancy test is still required on D1. The results of FPG, HbA_1c_, fasting insulin, fasting C-peptide, and fasting blood lipids measured on D1 will serve as baseline values. The first dose will be administered after all examinations on D1 are completed.

1. Demographic data include the date of birth, gender, and ethnicity.
2. Collection of medical history data: Including obesity/overweight medical history (including combined pre-diabetes, hypertension, dyslipidemia, fatty liver, and obstructive sleep apnea syndrome caused by overweight), other important medical histories, personal history, etc.
3. Prior treatment history: Including treatments for target indications and treatments for other diseases.
4. Randomization: It should be completed before the first dose.
5. Vital signs: Including blood pressure (mmHg), body temperature (°C), and pulse (beats/min). Before vital signs measurement, the subject should rest for at least 5 min and the vital signs will be measured when the subject is at rest. When there is a conflict between the scheduled time for measuring vital signs and the time of blood sampling, vital signs should be measured first.
6. A complete physical examination will cover the skin/mucosa, lymph nodes, head and neck (including thyroid glands), chest (heart, lung), abdomen (liver, gallbladder, spleen, and kidneys), musculoskeletal system, nervous system, etc.
7. The investigator will perform a symptom-directed physical examination based on clinical observations and symptoms during the treatment period.
8. 12-lead ECG: The heart rate, PR interval, RR interval, QRS, QT interval, QTcF, and ECG diagnostic results will be recorded. Subjects should rest for at least 5 min prior to ECG measurement. If the QTcF exceeds 450 msec or QRS exceeds 120 msec during the screening period, ECG examination should be repeated twice (on the day of screening, with at least 1 min apart from each other), and the mean of the 3 QTcF or QRS values will be used to determine the subject's eligibility.
9. Hematology: Including white blood cell count (WBC), red blood cell count (RBC), hemoglobin (Hb), platelet count (PLT), hematocrit (HCT), neutrophil count (NEUT#), neutrophil percentage (NEUT%), lymphocyte count (LYMPH#), lymphocyte percentage (LYMPH%), eosinophil count (EO#), eosinophil percentage (EO%), basophil count (BASO#), basophil percentage (BASO%), monocyte count (MONO#), and monocyte percentage (MONO%).
10. Blood chemistry: It should be tested under a fasting state before drug administration, including total protein (TP), albumin (ALB), globulin (GLO), total bilirubin (TBIL), direct bilirubin (DBIL), alanine aminotransferase (ALT), aspartate aminotransferase (AST), urea/urea nitrogen (BUN), creatinine (Cr), uric acid (UA), lactate dehydrogenase (LDH), creatine kinase (CK), creatine kinase isoenzyme (CK-MB), alkaline phosphatase (ALP), gamma-glutamyltranspeptidase (GGT), potassium (K), sodium (Na), chloride (Cl), magnesium (Mg), calcium (Ca), and phosphorus (P).
11. Urinalysis: Including urine pH, urine protein (PRO), urine ketone body (KET), urine specific gravity (SG), urine occult blood (BLD), urine glucose (GLU), urobilinogen (URO), urine bilirubin (BIL), urine red blood cells, and urine white blood cells.
12. Blood pregnancy test: Only applicable to female subjects of childbearing potential. Female subjects who are postmenopausal for more than 1 year or permanently sterilized (by tubal ligation, hysterectomy, bilateral salpingectomy, etc.) are considered non-childbearing. During the study, a blood pregnancy test should be performed immediately to rule out or validate a pregnancy no matter when a female subject reports no menstrual period or irregular menstrual cycles. The investigational product should be discontinued instantly if the blood pregnancy test is positive.
13. Thyroid function test: Including thyroid stimulating hormone (TSH), free tri-iodothyronine (FT_3_), and free thyroxine (FT_4_).
14. Coagulation function test: Including international normalized ratio (INR), activated partial thromboplastin time (APTT), and prothrombin time (PT).
15. Four items of infection: Including hepatitis B virus surface antigen (HBsAg), hepatitis C virus (HCV) antibody, human immunodeficiency virus (HIV) antibody, and treponema pallidum antibody (Tp-Ab) tests; if HBsAg is positive, hepatitis B virus deoxyribonucleic acid (HBV-DNA) quantitative detection should be performed.
16. B-mode ultrasound in liver, gallbladder, pancreas, spleen, and bilateral kidneys: The examination sites include liver, gallbladder, pancreas, spleen, and bilateral kidneys.
17. Body height: It will be expressed in cm (with 1 decimal place). See Appendix 2 for detailed measurement criteria.
18. Fasting body weight: It will be expressed in kg (with 1 decimal place). All measurements will be repeated twice. See Appendix 2 for detailed measurement criteria.
19. Fasting waist circumference: Waist circumference is defined as the circumference measured at the midpoint between the anterior superior iliac spine and the lower edge of costal arch on bilateral midaxillary lines. It will be expressed in cm (with 1 decimal place). All measurements will be repeated twice. See Appendix 2 for detailed measurement criteria.
20. Fasting blood lipids: Including total cholesterol (TC), triglycerides (TG), high-density lipoprotein cholesterol (HDL-C), and low-density lipoprotein cholesterol (LDL‑C).
21. Sparse PK blood sampling: It will be performed in all subjects, and the sampling time points are detailed in the "PK Sampling Schedule".
22. Intensive PK blood sampling: It is planned to be performed in approximately 40 subjects, and the sampling time points are detailed in the "PK Sampling Schedule".
23. Diary card: The diary card will be dispensed after the first dose at each follow-up visit, and the subjects will be instructed to complete the diary card, such as AEs, concomitant medications, and dosing records. At the next follow-up visit, the subjects should bring the diary card dispensed in the previous period back to the site, and the investigator will enter corresponding data according to the records on the diary card, evaluate drug administration of the subjects, and give corresponding instructions and suggestions to the subjects.
24. Diet and exercise instructions: The investigator should provide the subjects with individualized diet advice and exercise instructions according to the requirements of the study site. In case of unreasonable living habits (such as overeating), it is necessary to strengthen the instructions on the subjects.

PK Sampling Schedule

Subjects undergoing sparse PK blood sampling

| **Visit** | **Time (day)** | **PK sampling time point** |
| --- | --- | --- |
| V1 | D1 | 1–3 h after administration |
|  |  | 5–8 h after administration |
| V2 | D15 ± 3 | Before administration |
| V3 | D29 ± 3 | Before administration |
| V4 | D43 ± 3 | Before administration |
| V5 | D57 ± 3 | Before administration |
|  |  | 1–3 h after administration |
|  |  | 5–8 h after administration |
| V6 | D85 ± 3 | Before administration |
| Early withdrawal | Unscheduled (as soon as possible) | - |

Note:

1. For V2–V6, if the subject has taken the investigational product in advance before he/she arrives at the study site, PK samples can still be collected, and the dosing and sampling time points should be recorded as accurately as possible.

Subjects undergoing intensive PK blood sampling

| **Visit** | **Time (day)** | **PK sampling time point** |
| --- | --- | --- |
| V1 | D1 | 1–3 h after administration |
|  |  | 5–8 h after administration |
| V2 | D15 ± 3 | Before administration |
| V3 | D29 ± 3 | Before administration |
| V4 | D43 ± 3 | Before administration |
| V5 | D57 ± 3 | Within 1 h before administration |
|  |  | 0.5 h ± 3 min after administration |
|  |  | 1 h ± 5 min after administration |
|  |  | 2 h ± 10 min after administration |
|  |  | 4 h ± 10 min after administration |
|  |  | 6 h ± 30 min after administration |
|  |  | 8 h ± 30 min after administration |
|  |  | 10 h ± 1 h after administration |
|  |  | 12 h ± 1 h after administration |
|  | NA | 24 h ± 1 h after administration |
| V6 | D85 ± 3 | Before administration |
| Early withdrawal | Unscheduled (as soon as possible) | - |

Notes:

1. For V2–V6, if the subject has taken the investigational product in advance before he/she arrives at the study site, PK samples can still be collected, and the dosing and sampling time points should be recorded as accurately as possible.
2. The subjects will be hospitalized at V5 and can be discharged after the completion of PK blood sampling at 24 h after administration.
3. If intensive sampling cannot be performed within the visit window of V5, it can be delayed to V6 (D85 ± 3).

# 1 Introduction

# 1.1 Theoretical Basis of the Study

Overweight and obesity are chronic metabolic diseases characterized by excessive accumulation and/or abnormal distribution of body fat. Their pathogenesis includes genetic, metabolic, environmental, and behavioral factors. In recent years, the prevalence of overweight and obesity in China has sharply increased, making it the country with the highest number of obese individuals globally ^[1]^. According to statistics, the rates of overweight and obesity among Chinese adults are 34.3% and 16.4%, respectively ^[2][3]^. Numerous studies have shown that overweight and obesity are significant risk factors for common chronic diseases such as diabetes, cardiovascular diseases, non-alcoholic fatty liver disease, sleep apnea syndrome, and cancer ^[4]^. When obese patients lose 5% to 15% or more of their weight, the long-term prognosis of obesity-related diseases such as hypertension, dyslipidemia, and non-alcoholic fatty liver disease can be significantly improved, and blood glucose control in obese patients with type 2 diabetes mellitus (T2DM) can also be markedly enhanced, while the incidence of cardiovascular complications decreases ^[5][6]^.

Changes in lifestyle factors such as diet, nutrition, and exercise, as well as shifts in socioeconomic production models, are the main reasons for the annual increase in the prevalence of overweight and obesity ^[7]^. Both domestic and international guidelines ^[8][9]^ recommend dietary, exercise, and behavioral interventions as the first-line treatment options for weight loss. Other treatment methods include traditional Chinese medicine, pharmacotherapy, and metabolic weight loss surgery ^[7]^. Metabolic weight loss surgery, as an invasive treatment option, is not suitable for most obese patients. Beyond lifestyle modifications and surgical interventions, there is an urgent clinical need to better control the weight of overweight and obese patients through pharmacotherapy. Currently, there are seven weight loss medications approved by the U.S. Food and Drug Administration (FDA) that are still in use, including benzphetamine, phendimetrazine, phentermine, phentermine-topiramate, orlistat, liraglutide (SAXENDA), and semaglutide (WEGOVY). As of October 2023, the only drugs approved in China for the treatment of obesity are ^[10]^ orlistat, benaglutide (Phisomai®), and liraglutide (Liraglutide®). However, the weight loss effect of orlistat is limited ^[11]^. Benaglutide, liraglutide, and semaglutide are glucagon-like peptide-1 receptor agonists, which have become popular targets for clinical application and research of weight loss medications in recent years. Clinical trials and applications have shown significant efficacy in reducing patient weight and good safety profiles. Benaglutide and liraglutide require daily subcutaneous injections, making them less convenient to use; therefore, there remains a substantial unmet clinical need for pharmacotherapy for obesity in China.

Glucagon-like peptide-1 (GLP-1) binds to and activates the GLP-1 receptor (GLP-1R), which is part of the class B family of G protein-coupled receptors, to exert its regulatory functions. GLP-1 receptor agonists (GLP-1RAs) are synthetically produced drugs designed to mimic the physiological functions of GLP-1. After binding to the GLP-1R on pancreatic cells, they stimulate insulin synthesis and secretion in a glucose-dependent manner and reduce glucagon release. Additionally, GLP-1RAs can promote weight loss through central and peripheral mechanisms, with the main mechanisms including:

（1）Appetite Suppression: It inhibits appetite and reduces food intake by significantly increasing the levels of satiety signals in the arcuate nucleus of the hypothalamus and suppressing the increase of hunger signals in the arcuate nucleus, thereby enhancing feelings of fullness and reducing caloric intake.

（2）Increase Energy Expenditure: It promotes the conversion of visceral white fat to brown fat and enhances thermogenesis in brown fat.

（3）Gastrointestinal Effects: It acts on the gastrointestinal tract to delay gastric emptying and gastrointestinal motility, and reduces gastric acid secretion stimulated by gastrin ^[12][13]^.

VCT220 is a new oral small molecule GLP-1 receptor agonist (GLP-1RA) independently developed by Vincentage Pharma Co., Ltd.classified as a Class 1 chemical drug. VCT220 is designed, screened, modified, and improved based on the three-dimensional structure of the GLP-1R receptor protein, resulting in a small molecule GLP-1RA with good oral bioavailability.In non-clinical models, VCT220 has been shown to stimulate glucose-dependent insulin release, lower blood glucose levels, and suppress food intake, demonstrating efficacy similar to that of injectable peptide GLP-1RAs approved for the treatment of type 2 diabetes mellitus (T2DM), with no significant safety risks. Clinically, it can be used for blood glucose control in adult T2DM patients and weight management in obese patients.

## 1.2 Overview of Preclinical Studies

The preclinical studies of VCT220 tablets have been completed, demonstrating that VCT220 tablets possess good safety, efficacy, and oral pharmacokinetic properties. Detailed descriptions can be found in the VCT220 Investigator's Brochure ^[14]^.

### 1.2.1 Pharmacodynamic Studies

In vitro studies have confirmed that VCT220 can competitively bind to GLP-1 receptors (GLP-1R), exhibiting strong agonistic activity on both human and monkey GLP-1Rs. It dose-dependently promotes the production of cAMP and recruits β-Arrestin-1 and β-Arrestin-2, leading to the internalization of GLP-1R, which facilitates the expression of its pharmacological activity.

In vivo studies have confirmed that in glucose tolerance tests conducted on Cynomolgus monkey and hGLP-1R mice, both oral and intravenous administration of VCT220 can dose-dependently promote insulin secretion and control the increase in blood glucose levels. At oral dosing levels, there is a significant reduction in food intake in Cynomolgus monkey, along with a notable weight loss effect.

### 1.2.2 Pharmacokinetic Studies

The pharmacokinetic (PK) studies of VCT220 evaluated its absorption, distribution, metabolism, and excretion characteristics. Based on the identification of hepatic metabolites, rats and Cynomolgus monkey were confirmed as relevant animal species for the studies. The pharmacokinetic in vivo studies were conducted using SD rats and Cynomolgus monkey. After continuous oral administration of 50 mg/kg VCT220 to SD rats for 7 days, and 20 mg/kg VCT220 to Cynomolgus monkey for 7 days, the systemic exposure levels before and after the first and last doses remained essentially unchanged, with no accumulation observed.

The intestinal absorption permeability assessment indicates that VCT220 has moderate permeability and may be a weak substrate for efflux transporters. Its absorption behavior may be influenced by the efflux of P-glycoprotein transporters.

VCT220 exhibits a very high plasma protein binding rate, which is not concentration dependent. It is primarily distributed in the small intestine, stomach, and liver, with lower distribution in the heart and brain. The order of tissue distribution concentration of the drug, from highest to lowest, is as follows: small intestine > stomach > liver > kidney > heart > lung > ovary (female) > spleen > subcutaneous fat > muscle > testis (male) > brain.

In vitro metabolism results indicate that the terminal elimination half-life (t1/2) of VCT220 in human, monkey, dog, rat, and mouse liver microsomes are 122, 45.0, 231.05, 39.6, and 107 minutes, respectively. The intrinsic clearance (CLint) values are 14.3, 45.0, 0.150, 62.7, and 51.2 mL/min/kg, respectively. VCT220 is metabolized slowly in dogs and at a moderate rate in humans, monkeys, rats, and mice. In vivo metabolism results show that VCT220 undergoes extensive metabolism in rats, with metabolic pathways similar in vivo and in vitro. A total of 50 metabolites were detected, in addition to the parent drug (VCT220). The metabolic pathways include N-dealkylation, O-dealkylation, mono-oxidation, di-oxidation, tri-oxidation, dehydrogenation, cysteine conjugation, and glucuronidation, among which O-dealkylation (M3), mono-oxidation (M9), and glucuronidation (M16) are the major metabolic pathways.

According to the excretion study conducted with SD rats following oral administration, after administering 50 mg/kg of VCT220, the parent drug is primarily excreted in the feces. The average recovery rates of VCT220 in urine, feces, and bile are 0.04%, 17.60%, and 4.41%, respectively.

### 1.2.3 Toxicology Studies

In a single-dose toxicity study, VCT220 was administered via gavage to SD rats at doses of 500, 1000, and 2000 mg/kg (based on free acid), with the maximum tolerated dose (MTD) being 2000 mg/kg. VCT220 was also administered via gavage to Cynomolgus monkey at doses of 150, 300, and 600 mg/kg (based on free acid), with the MTD being 600 mg/kg.

In a 16-week repeated dose toxicity study, the no observable adverse effect level (NOAEL) of VCT220 in SD rats (at doses of 75, 150, and 300 mg/kg) was found to be 400 mg/kg. In Cynomolgus monkey (at doses of 40, 80, and 120 mg/kg), the NOAEL was determined to be 120 mg/kg. After 16 weeks of repeated dosing, the pharmacokinetic study results in both SD rats and Cynomolgus monkey indicated that there were no significant gender differences in systemic exposure and no potential drug accumulation of VCT220.

In the genotoxicity study, VCT220 showed no mutagenic effects on Salmonella typhimurium strains TA97a, TA98, TA100, TA102, and TA1535. Under both metabolic and non-metabolic activation conditions, after a 4-hour exposure for all dose groups of VCT220, and a 24-hour exposure under non-metabolic activation conditions, there were no significant increases in the rate of chromosomal aberrations compared to the solvent control group (P > 0.05). The results were negative, indicating no teratogenicity. CD-1 mice were administered VCT220 at doses of 300, 800, and 2000 mg/kg via gavage for 2 consecutive days, and no genotoxic effects on bone marrow cells were observed.

In the reproductive toxicity study conducted on SD rats (100, 300, and 500 mg/kg) for fertility and early embryonic development toxicity (1st segment of reproductive toxicity), the NOAEL was found to be 500 mg/kg. In the embryonic-fetal development toxicity study (2nd segment of reproductive toxicity) on SD rats (100, 300, and 600 mg/kg), the NOAEL for the parent female rats was 300 mg/kg, while the NOAEL for embryonic-fetal development was 100 mg/kg. In the embryonic-fetal development toxicity study (2nd segment of reproductive toxicity) on New Zealand rabbits (50, 150, and 300 mg/kg), the NOAEL for the maternal subjects was 150 mg/kg, and the NOAEL for embryonic-fetal development was 300 mg/kg.

## 1.3 Overview of Clinical Studies

VCT220 tablets have undergone three clinical studies, including one Phase Ia study (CTR20222374) and two Phase Ib studies (CTR20231482 and CTR20230826), among which:

Phase Ia Study

- The Phase Ia study (CTR20222374) is a clinical research conducted on healthy adult subjects in China. The primary objective is to evaluate the safety and tolerability of a single oral dose of VCT220 tablets in healthy adult participants. The secondary objective is to assess the pharmacokinetic (PK) characteristics of the plasma following a single oral administration.
- Single-Dose Escalation Study：In the single-dose escalation study, the safety evaluation analysis results showed that VCT220 demonstrated good overall tolerability and safety in the dose range of 10-300 mg. Adverse events (AE) were primarily gastrointestinal and neurological reactions, all classified as Grade 1 and Quick Recoveryy. The incidence of AEs and adverse reactions showed a gradual increase trend with the increase in dosage. The specific list of AEs is as follows：

Table 1: Adverse Events of Single Oral Dose of VCT220 Tablets in Phase Ia Study in Healthy Chinese Subjects

| Adverse Reactions | Dosage（mg） | | | | | | | | |
| --- | --- | --- | --- | --- | --- | --- | --- | --- | --- |
|  | (N=12) | 10  (N=8) | 30  (N=8) | 60  (N=8) | 120  (N=8) | 120 Food effects | | 200  (N=8) | 300  (N=8) |
|  |  |  |  |  |  | Fasting  (N=12) | after meals(N=12) |  |  |
| Nausea | 0 | 0 | 0 | 5(62.5) | 3(37.5) | 4(33.3) | 3(25.0) | 8(100) | 5(62.5) |
| Vomiting | 0 | 0 | 0 | 0 | 1(12.5) | 2(16.7) | 0 | 7(87.5) | 4(50.0) |
| Decreased Appetite | 0 | 0 | 0 | 0 | 0 | 3(25.0) | 6(50.0) | 4(50.0) | 8(100) |
| Abdominal Pain | 0 | 0 | 0 | 0 | 0 | 0 | 0 | 0 | 1(12.5) |
| Diarrhea | 0 | 0 | 0 | 0 | 0 | 1(8.30) | 0 | 0 | 0 |
| Dizziness | 0 | 0 | 0 | 0 | 2(25.0) | 3(25.0) | 2(16.7) | 7(87.5) | 7(87.5) |
| Headache | 0 | 0 | 0 | 0 | 1(12.5) | 4(33.3) | 3(25.0) | 0 | 4(50.0) |
| Fatigue | 0 | 0 | 0 | 0 | 0 | 1(8.30) | 0 | 1(12.5) | 2(25.0) |
| Tachycardia | 0 | 0 | 0 | 0 | 0 | 0 | 0 | 0 | 1(12.5) |
| Palpitations | 0 | 0 | 0 | 0 | 0 | 0 | 0 | 0 | 2(25.0) |
| （ALT）  Alanine aminotransfer-ase elevation (ALT) | 0 | 0 | 0 | 0 | 0 | 0 | 1(8.30) | 0 | 0 |

After a single oral administration of 10-300 mg VCT220 tablets to the subjects, there were no significant changes in t1/2 among the groups. However, with increasing doses, the time to reach peak concentration (Tmax) was delayed (median values ranged from 0.99 to 6.99 hours), while the mean residence time (MRT) of the drug was prolonged (average values ranged from 5.35 to 12.91 hours), showing a clear dose-related response. The mean elimination half-life (t1/2) of the drug ranged from 4 to 5 hours. Within the 10-300 mg dose range, Tmax increased with the administration dose, and the AUC showed a largely linear relationship. Additionally, there were no gender differences among the groups.

Studies on the effects of food indicate that at a dose of 120 mg, food has no significant effect on the AUC0-t and AUC0-∞ of orally administered VCT220. However, taking the medication after a meal resulted in a flatter drug concentration-time curve, with a mean Cmax reduction of 25.09%. Given the limited impact of food, future studies may not need to account for dietary influences.

Exploratory material balance studies indicate that after a single oral administration of VCT220 tablets, the cumulative excretion rate of the parent compound in urine is less than 1% of the administered dose, and the cumulative excretion rate of the parent compound in feces is less than 2%. This suggests that VCT220 is primarily eliminated through metabolic processes in the body. After taking a 120 mg dose of VCT220, the renal clearance of VCT220 is measured at 0.28±0.05 mL/h.

Preliminary results report the pharmacokinetic outcomes of healthy Chinese subjects after a single oral dose of varying amounts of VCT220 tablets as follows:

**Table 2: Pharmacokinetic Parameters (Mean) of VCT220 Tablets After Single Oral Administration in Healthy Chinese Subjects**

| **Dose Group** | **t_1/2_**  **(hr)** | **T_max_**  **(hr)** | **C_max_**  **(ng/ml)** | **AUC_0-t_**  **(hr*ng/ml)** | **AUC_0-∞_**  **(hr*ng/ml)** |
| --- | --- | --- | --- | --- | --- |
| **Single-dose escalation study** | | | | | |
| 10mg | 3.69 | 0.99 | 222.33 | 1436.01 | 1449.43 |
| 30mg | 4.51 | 1.25 | 791.00 | 6461.10 | 6494.78 |
| 60mg | 4.60 | 3.38 | 1163.50 | 11287.37 | 11311.84 |
| 120mg | 4.53 | 4.48 | 2000.00 | 24365.30 | 24469.55 |
| 200mg | 3.87 | 3.50 | 3346.25 | 38430.85 | 38506.28 |
| 300mg | 6.75 | 6.99 | 3856.25 | 61546.06 | 63185.43 |
| **Food Effect Study** | | | | | |
| 120mg FAST | 4.27 | 5.98 | 2325.83 | 24057.68 | 24157.40 |
| 120mg FED | 4.54 | 5.98 | 1742.08 | 25276.00 | 25408.24 |
| Note: Tmax is expressed as the median (minimum, maximum).  The data is sourced from the "Statistical Analysis Report," Table 13-33, parameters of VCT220 after a single oral administration of 10 mg to 300 mg in healthy subjects. | | | | | |

**Phase Ib Study**

The Phase Ib study (CTR20231482) is a randomized, double-blind, placebo-controlled study conducted in overweight/obese adult subjects in China. The primary objective is to evaluate the safety, tolerability, and PK characteristics of multiple oral administrations of VCT220 in overweight/obese subjects who are controlling their weight solely through diet and exercise. The study has been completed.

In the Phase Ib study involving overweight/obese subjects, three groups received oral administrations of VCT220 at doses of 100 mg, 150 mg, and 200 mg (once daily, QD) for 28 consecutive days. The overall tolerability was good, with all adverse events (SAE) being mild to moderate, and no serious adverse events (SAE) or deaths reported. No subjects withdrew from the study. Similar to other drugs in the same category, the most common adverse reactions were gastrointestinal-related (such as nausea and vomiting), which were mostly mild and well tolerated by the subjects. Other adverse reactions included some abnormal laboratory findings, decreased appetite, and clinically insignificant electrocardiogram abnormalities, all of which were mild in severity. In summary, this study indicates that VCT220 within the 100 mg to 200 mg range demonstrates good safety and Tolerance

In the Phase Ib study, overweight/obese subjects in each group exhibited a significant reduction in weight after 28 days of VCT220 administration. Relative to baseline data, the percentage weight reduction on Day 28 for the 100 mg, 150 mg, and 200 mg groups was as follows:5.2%，4.9%，5.0%In contrast, the placebo group showed a weight reduction of only 1.0%.

Preliminary PK results indicate that within the dose range of 100-200 mg, the PK parameters of the last administration of VCT220 exhibit linear pharmacokinetic characteristics with respect to dosage. After 28 days of continuous dosing, there was no significant accumulation of the drug. The PK results are shown in the table below:

**Table 3: PK Parameters Table for Overweight/Obese Subjects on Day 1**

|  | Group 1 - 100 mg | Group 2-150mg | Group 3-200mg |
| --- | --- | --- | --- |
|  | (N=8) | (N=8) | (N=6) |
| C_max_（ng/mL） | 919.38±134.40 | 862.13±75.98 | 839.00±218.95 |
| AUC_0-t_（h*ng/mL） | 8775.91±1987.84 | 8321.76±1689.15 | 7513.83±2838.05 |
| AUC_0-∞_（h*ng/mL） | 9113.26±2230.63 | 8730.07±1944.16 | 7749.59±3006.68 |
| T_max_（h） | 4.13±1.25 | 5.59±1.03 | 5.17±0.41 |
| t_1/2z_（h） | 4.40±0.71 | 4.41±0.65 | 3.86±0.74 |
| CL_z_/F（mL/h） | 11448.70±2275.81 | 17923.38±3852.78 | 29377.60±11139.34 |
| V_z_/F（mL） | 71038.09±10054.36 | 111161.7±13771.41 | 156778.4±46305.46 |
| MRT_0-t_（h） | 7.76±1.07 | 8.59±0.91 | 8.05±1.04 |
| MRT_0-∞_（h） | 8.52±1.28 | 9.52±1.31 | 8.62±1.37 |
| Note: The units for the PK parameters should be modified according to the actual situation. | | | |

**Table 4: PK Parameters Table for Overweight/Obese Subjects on Day 29**

|  | Group 1-100mg | Group 2-150mg | Group 3-200mg |
| --- | --- | --- | --- |
|  | (N=8) | (N=8) | (N=6) |
| C_max,ss_（ng/mL） | 1558.50±483.12 | 1894.49±1175.79 | 2791.67±1061.35 |
| AUC_0-τ,ss_（h*ng/mL） | 17864.72±5455.19 | 26749.33±15819.86 | 35684.47±15780.00 |
| C_min,ss_（ng/mL） | 182.20±110.40 | 341.29±230.72 | 507.33±407.42 |
| AUC_av,ss_（ng/mL） | 744.36±227.30 | 1114.56±659.16 | 1486.85±657.50 |
| DF（％） | 187.01±55.53 | 178.71±112.49 | 165.54±70.95 |
| S_wing_ | 9.57±5.47 | 4.75±1.44 | 8.90±10.78 |
| T_max,ss_（h） | 6.88±2.69 | 4.72±0.90 | 6.17±4.07 |
| V_z_/F（mL） | 51362.54±20353.87 | 231965.3±459596.5 | 45129.39±21377.94 |
| CL_ss_/F（mL/h） | 6018.19±1640.58 | 52033.21±123352.7 | 6446.39±2435.24 |
| t_1/2z_（h） | 5.44±1.38 | 6.42±3.15 | 4.72±0.75 |
| λ_z_（1/h） | 0.13±0.03 | 0.13±0.07 | 0.15±0.02 |
| AUC_％Extrap_（％） | 7.35±3.76 | 12.64±8.03 | 5.66±2.96 |
| MRT_0-∞_（h） | 11.34±1.87 | 13.08±4.62 | 10.51±2.55 |
| R_a(Cmax)_（％） | 0.84±024 | 0.74±0.46 | 0.85±0.35 |
| R_a(AUC)_（％） | 1.01±0.18 | 1.04±0.51 | 1.19±0.21 |

## 1.4 Risk-Benefit Assessment

**Known Potential Risks**

Based on the results of completed non-clinical studies and Phase Ia and Ib clinical trials, the theoretical or potential safety risks of VCT220 tablets are summarized as follows:

**Table 5: Summary of Theoretical or Potential Safety Risks of VCT220 Tablets**

| **Potential Risks** | **KnInformation or Pown ossibilities** | **Risk Minimization Measures** |
| --- | --- | --- |
| Coagulation Function | Coagulation Function  In the toxicity study of VCT220 conducted through repeated gavage administration to SD rats for 4 weeks, followed by a 4-week recovery period, the results showed that in the 750 mg/kg dose group, the Prothrombin Time (PT) and Activated Partial Thromboplastin Time (APTT) on Day 29 exhibited a reduction related to the test substance. By the end of the 4-week recovery period, these parameters returned to levels comparable to the solvent control group. | The toxicity dose levels observed in the study were significantly higher than the intended clinical doses, and the effects were noted to occur in isolated instances within individual animals, indicating a lower risk profile. During clinical research, it is important to closely monitor any abnormalities in human subjects. If any adverse effects are observed, the study should be promptly terminated to ensure participant safety. |
| Hepatotoxicity | The toxicity study of VCT220 involved repeated gavage administration to SD rats for 4 weeks, followed by a 4-week recovery period. In the 750 mg/kg dosage group, the biochemical indicators on Day 29 (D29) showed increases in ALT (Alanine Aminotransferase), alkaline phosphatase (ALP), and total bilirubin (TBil), all of which were associated with the test substance. | The toxicity dose levels are significantly higher than the intended clinical dosage, and the occurrences were observed in individual animals, indicating a lower risk. In clinical studies, it is crucial to focus on monitoring; if any abnormalities are detected in humans, the study should be promptly terminated. |
| Malnutrition | The toxicity study of VCT220 involved repeated gavage administration to SD rats for 4 weeks, followed by a 4-week recovery period, focusing on body weight and food intake:  1. Compared to the same-sex solvent control group, male animals in the 750 mg/kg dosage group showed a decrease in body weight, weight gain, and food intake during the administration period, with the highest reductions of -12.2%, -37.4%, and -20.5%, respectively. A recovery trend was observed during the 4-week recovery period.  2. Similarly, compared to the same-sex solvent control group, female animals in the 750 mg/kg dosage group also exhibited a decrease in body weight, weight gain, and food intake during the trial period, with the highest reductions of -7.6%, -73.3%, and -31.8%, respectively.  3. Triglycerides (TG) showed a decrease associated with the test substance, and a significant reduction in total protein (TP) and albumin (ALB) was also observed in female animals on Day 29 (D29). | The decrease in TG (triglycerides), TP (total protein), and ALB (albumin) may also be related to the product's appetite-suppressing effects, leading to reduced intake of fats and proteins. It is important to monitor for the occurrence of malnutrition due to excessive appetite suppression in clinical studies. |
| Gastrointestinal adverse reactions | In the Phase Ia study of VCT220 tablets, the 60 mg single-dose group experienced nausea; the 120 mg to 300 mg single-dose groups reported nausea, vomiting, and some participants experienced a decrease in appetite.  In the Phase Ib study of VCT220 tablets with repeated administration over 28 days, participants in the 100 mg to 200 mg dose groups all reported gastrointestinal adverse reactions, including nausea, vomiting, decreased appetite, abdominal distension, and diarrhea. | During the clinical study visits, participants were monitored through weight, vital signs, and laboratory assessments to prevent any potential sequelae of severe gastrointestinal reactions (e.g., dehydration).  The clinical study was conducted using a titration approach to reach the target dose. The process emphasized monitoring the tolerance of participants, and timely medical interventions were provided for gastrointestinal reactions reported by the subjects. |
| Cardiovascular and cerebrovascular adverse reactions | In the phase I study of VCT220 tablets, adverse reactions were observed in the 120-200 mg single-dose groups, including headache and dizziness. In the 300 mg group, adverse reactions included headache, dizziness, palpitations, and increased heart rate/pulse.  In the phase Ib study of VCT220 tablets with repeated dosing over 28 days, subjects in the 100 mg - 200 mg dose groups experienced dizziness, headache, and electrocardiogram abnormalities without any clinical significance, all of which were of mild intensity. | Initiating treatment with a low dose and gradually titrating to the clinical target dose, the trial closely monitored the safety of the subjects throughout the study period. The clinical research process focused on the subjects' tolerance, providing timely medical intervention for any related adverse events reported by the subjects. |
| Laboratory Tests | In the VCT220 tablet Phase I study, one subject experienced an increase in ALT after administering 120 mg with food (incidence rate: 1/12).  In the VCT220 tablet Phase Ib study, some subjects experienced an increase in AST and ALT during the treatment period. | The occurrence of elevated ALT was observed in only one case among the 48 subjects who received 120 mg or higher, indicating no clear dose-related correlation. Continued monitoring in future studies is required.  During the 4-week treatment period of the Phase Ib study, no interventions or discontinuations were made for the subjects. All cases returned to normal within 1-2 weeks, with no clear dose-related correlation observed. Continuous monitoring will be conducted in subsequent clinical studies. |
| Medullary Thyroid Carcinoma (MTC) | Potential risks based on the product information for injectable GLP-1 receptor agonists (namely liraglutide, dulaglutide, and exenatide) include dose-dependent and treatment duration-dependent medullary thyroid carcinoma observed at clinically relevant exposure levels in non-clinical studies in rats and mice. | Potential subjects with a personal or family history of medullary thyroid carcinoma or MEN2 are not allowed to participate in clinical development programs.  Non-clinical carcinogenicity studies for this product will be initiated at specific development stages in accordance with relevant guidelines. |
| Pancreatitis | Potential risks are based on the product labels of injectable GLP-1 receptor agonists (i.e., liraglutide, exenatide, and dulaglutide). | According to the exclusion criteria, potential participants with a history of or currently suffering from pancreatitis (chronic pancreatitis or acute pancreatitis) are not eligible for enrollment in the study. Serum amylase and lipase will be monitored during the clinical trial. |
| Acute kidney injury (AKI) | Potential risks based on the product instructions for the injectable GLP-1 receptor agonist semaglutide for the treatment of type 2 diabetes. | Exclude potential subjects with a known history of renal impairment from clinical studies. |
| Allergic Reactions | Potential risks based on the product label of the injectable GLP-1 receptor agonist semaglutide for the treatment of type 2 diabetes. | If a serious allergic reaction related to the medication is suspected, terminate the trial and seek medical advice immediately. |

Two GLP-1 receptor agonists indicated for overweight and obesity, liraglutide (SAXENDA) and semaglutide (WEGOVY), have been approved in the United States, both demonstrating good safety and tolerability. In addition to the most common gastrointestinal adverse effects, liraglutide treatment carries risks of hypoglycemia (1.6%, with blood sugar below 54 mg/dL), allergic reactions (0.7%), and injection site reactions (13.9%). Similarly, semaglutide has also reported risks of hypoglycemia (6%, with blood sugar below 54 mg/dL), allergic reactions (3%), and injection site reactions (1.4%). The oral small molecule GLP-1 receptor target drug Orforglipron (LY3502970), currently in development, has shown in a double-blind, randomized, placebo-controlled phase II clinical trial involving overweight/obese subjects that its overall safety profile is consistent with the established characteristics of GLP-1 RA drugs. Gastrointestinal reactions are common adverse events (AEs), with incidence rates similar to those of other GLP-1 RA drugs, including nausea, constipation, vomiting, and diarrhea, usually occurring in the initial stages of administration. The severity ranges from mild to moderate, and as the dosage increases, subjects gradually develop tolerance, leading to a decrease in incidence.

In summary, based on the non-clinical and clinical research data of VCT220 as well as the reported safety data of other GLP-1 receptor agonists, the adverse events (AEs) to be monitored in this clinical trial include, but are not limited to, gastrointestinal adverse events, allergic reactions, and hypoglycemic events. The investigators will closely monitor AEs during the trial, and any adverse events will be promptly assessed and addressed.

**Known Potential Benefits**

Based on existing non-clinical data, VCT220 tablets exhibit potential efficacy in reducing weight and improving blood glucose levels.

The two GLP-1 RAs currently available in the United States, liraglutide and semaglutide, have demonstrated their efficacy and safety in adults and adolescents aged 12 and older with obesity. This suggests that VCT220, also a GLP-1 RA, may offer potential benefits in the treatment of overweight and obese patients that outweigh the potential risks. The oral small molecule GLP-1 receptor target drug Orforglipron (LY3502970) has shown in Phase II clinical studies involving obese or overweight participants that, in the 26-week primary endpoint evaluation, all doses of Orforglipron resulted in statistically significant dose-dependent weight loss, ranging from 8.6% to 12.6%, compared to only 2.0% in the placebo group. The weight of participants in the Orforglipron group continued to decrease at 36 weeks of treatment, with all doses showing weight reductions between 9.4% and 14.7%, while the placebo group saw only a 2.3% reduction. Among the different dosing groups, as many as 92% of participants experienced at least a 5% weight loss, and up to 75% achieved at least a 10% weight loss.

In summary, the GLP-1 RA drug VCT220 tablets have significant potential in weight management. Based on this, the sponsor intends to conduct a Phase II clinical trial of VCT220 tablets in non-diabetic overweight/obese participants who have poor weight control despite dietary and exercise interventions, in accordance with relevant national laws and regulations, to evaluate the efficacy and safety of VCT220 tablets.

**Assessment of Potential Risks and Benefits**

Based on a comprehensive evaluation, the benefits of this product outweigh the risks, thus supporting the planned clinical research.

# 2. Research Objectives

**Main Objectives**

- Evaluate the weight loss effects of different doses of VCT220 tablets in overweight/obese subjects without diabetes.

Secondary Objectives

- Evaluate the safety of different doses of VCT220 tablets in non-diabetic overweight/obese subjects.
- Evaluate the PK characteristics of different doses of VCT220 tablets in non-diabetic overweight/obese subjects.

Exploratory Objective

- Preliminarily explore the correlation between exposure, efficacy, and adverse events (AE) of VCT220 tablets in non-diabetic overweight/obese subjects.

# 3. Study Design

## 3.1 Overall Design

This study is a multicenter, randomized, double-blind, placebo-controlled Phase II clinical trial aimed at evaluating the efficacy, safety, and pharmacokinetic (PK) characteristics of different doses of VCT220 tablets in overweight/obese subjects without diabetes.

The study consists of three phases: the screening phase (up to 2 weeks), the treatment phase (16 weeks), and the safety follow-up phase (2 weeks).A total of 240 participants who met the inclusion criteria for this study were randomly assigned to the trial drug group and the placebo group in a 3:1 ratio, receiving either VCT220 tablets or placebo treatment. The specific grouping and number of participants in the study are as follows:80 mg dose group (N=60),80 mg dose matched placebo group (N=20),120 mg dose group (N=60),120 mg dose matched placebo group (N=20),160 mg dose fast titration group (N=30),160 mg dose fast titration matched placebo group (N=10),160 mg dose slow titration group (N=30),160 mg dose slow titration matched placebo group (N=10).

All dose groups were enrolled in parallel.The participants are enrolled in parallel across each dosage group.A total of 180 subjects received treatment with VCT220 tablets.A total of 60 subjects received treatment with a placebo.Randomly assigned subjects received the corresponding doses of VCT220 tablets or placebo treatment, taken once daily with breakfast for a continuous period of 16 weeks. After completing the 16 weeks of treatment, all subjects entered a 2-week safety follow-up period.

Throughout the entire study treatment period, PK blood samples will be collected from all subjects. All subjects are required to undergo sparse PK blood sample collection at the scheduled time points outlined in the protocol. Approximately 40 subjects will also undergo intensive PK blood sample collection at the same scheduled time points, including 10 subjects from the 80 mg dose group (or its placebo group), 10 subjects from the 120 mg dose group (or its placebo group), 10 subjects from the 160 mg dose slow titration group (or its placebo group), and 10 subjects from the 160 mg dose fast titration group (or its placebo group).

During the study period, investigator will provide education to subjects regarding medicationcompliance and individualized dietary and exercise guidelines. Subjects are expected to comply with the basic dietary and exercise requirements throughout the entire study.

The titration methods during the treatment period for each dose group (and its corresponding placebo group) are as follows:

|  | **W1** | **W2** | **W3** | **W4** | **W5** | **W6** | **W7-W16** |
| --- | --- | --- | --- | --- | --- | --- | --- |
| 80 mg Dose Group | 20mg | 40mg | 80mg | 80mg | 80mg | 80mg | 80mg |
| 120mg Dose Group | 20mg | 40mg | 60mg | 80mg | 120mg | 120mg | 120mg |
| 160mg Dose Fast Titration Group | 20mg | 40mg | 80mg | 120mg | 160mg | 160mg | 160mg |
| 160mg Dose Slow Titration Group | 20mg | 20mg | 40mg | 40mg | 80mg | 120mg | 160mg |

## 3.2 Basis for Research Design

According to the "Technical Guideline for Clinical Trials of Weight Control Drugs" ^[18]^, a randomized, double-blind, placebo-controlled study design was adopted to more objectively evaluate the efficacy of drug interventions. Participants who are obese and overweight were assessed using the BMI threshold values diagnosed for Chinese individuals. Based on the mechanism of action and intensity of the drug, the treatment duration of this exploratory study was set to 16 weeks to ensure it is long enough. A titration dosing regimen was implemented to determine the effective dose range that produces weight loss effects and the optimal titration scheme.

## 3.3 Basis for Dose Selection

The dose selection for VCT220 tablets in this study is primarily based on data obtained from a completed 28-day Phase Ib clinical study (Protocol Number: VCT220-I-06). This study evaluated the preliminary safety, tolerability, and pharmacokinetic/pharmacodynamic PK/PD) results of VCT220 tablets compared to a placebo in overweight/obese subjects, as well as exposure thresholds relative to toxicological results.

In the Phase Ib study involving overweight/obese participants, three groups received VCT220 tablets at doses of 100 mg, 150 mg, and 200 mg (QD) for 28 consecutive days. The overall tolerability was good, with adverse events (AE) reported as mild to moderate in severity. No serious adverse events (SAE) or deaths occurred, and no participants withdrew from the study. As with similar medications, the most common adverse reactions observed were gastrointestinal-related, such as nausea and vomiting. These were primarily mild, and participants were able to tolerate them well. Other adverse reactions included some laboratory test abnormalities, decreased appetite, and ECG changes that had no practical clinical significance, all of which were also mild in severity. In summary, this study indicates that VCT220 is well-tolerated and exhibits good safety within the 100 mg to 200 mg dose range. For more detailed information, please refer to the clinical study report and investigator's brochure.

In the Phase Ib study, overweight/obese participants experienced significant weight loss after 28 days of treatment with VCT220. The percentage changes in weight compared to baseline data were as follows:100 mg group: 5.2% 、150 mg group: 4.9%、 200 mg group: 5.1%、Placebo group: 1.0%

Based on these results, it is preliminarily concluded that the effective dose range for weight loss with VCT220 is between 100 mg and 200 mg QD. Given that even at the lower dose of 100 mg, participants showed substantial weight reduction, it is anticipated that longer treatment durations would yield even more pronounced changes. However, the increased duration of treatment necessitates higher safety requirements for the medication.

Therefore, this study proposes to evaluate three new dose levels—80 mg, 120 mg, and 160 mg, all administered once daily (QD)—to investigate the weight loss efficacy, safety, and pharmacokinetic characteristics over a 16-week treatment period.

Additionally, the recommended study doses outlined above are well-supported by the results of toxicological studies. In a 16-week toxicity study in rats (including a 4-week recovery period), the No Observed Adverse Effect Level (NOAEL) was found to be 300 mg/kg/day. The exposure margins for the clinical doses of 100 mg to 200 mg corresponded to 2.7 to 13.5 times (AUC0-t) and 9.4 to 16.8 times (Cmax) the observed human exposure levels. In a 16-week toxicity study in monkeys (also including a 4-week recovery period), the NOAEL was 120 mg/kg/day, with exposure margins for the clinical doses of 100 mg to 200 mg being 2.1 to 4.3 times (AUC0-t) and 3.1 to 5.5 times (Cmax) the observed human exposure levels.

Considering that the dosing period in Phase II studies is longer, and to ensure that participants can safely and steadily adhere to the medication, the starting dose has been adjusted to a lower level of 20 mg, which is below the 50 mg starting dose used in the Phase Ib study. This adjustment is in line with the experiences from ongoing studies of small molecular drugs, such as Pfizer's Danuglipron and Eli Lilly's Orforglipron, which suggest that “a lower starting dose and a slower dose escalation can reduce the frequency and severity of gastrointestinal adverse reactions.”

In this study, the dose will be adjusted weekly, with titration to the target dose over four weeks, followed by maintenance at the target dose for an additional twelve weeks. Based on the pharmacokinetic concentration data from the Phase Ib study, a linear regression was performed on the standardized blood drug concentrations (i.e., divided by the last measured concentration before dosing) for each dosing group, indicating that VCT220 reached steady-state by Day 26 of dosing. The 200 mg group, which has the shortest duration for reaching steady state, achieved this on Day 5 of steady-state dosing. Therefore, it is believed that steady-state can be reached by weekly dose adjustment. Additionally, a subgroup will be added within the 160 mg dose group to explore a slow titration approach: starting at 20 mg, with dose adjustments every two weeks for the first four weeks, followed by weekly adjustments after week 4 until reaching 160 mg for 6 weeks, and then maintaining at this dose for ten weeks. This examination aims to determine whether a slow titration approach can further enhance the safety and tolerability of the treatment.

## 3.4 Rationale for Endpoint Selection

According to the "Technical Guidelines for Clinical Trials of Weight Control Drugs" ^[18]^, it is recommended that the primary efficacy endpoints of weight control drugs be evaluated by analyzing the relative percentage change in weight from baseline and the response rate. The relative percentage change is defined as the difference between the percentage decrease in weight from baseline in the treatment drug group and the percentage decrease in weight from baseline in the placebo group. The response rate is defined as the difference between the proportion of subjects in the treatment drug group who experienced at least a 5% reduction in weight from baseline and the proportion of subjects in the placebo group who experienced at least a 5% reduction in weight from baseline.

Referring to the Phase II clinical trial results of similar GLP-1 RA drugs (such as the oral small molecule Orforglipron) in obese/overweight subjects, after 12-16 weeks of treatment, the percentage decrease in weight relative to baseline was significantly higher than that of the placebo group. Therefore, the primary efficacy endpoint of this study is set as the percentage change in weight from baseline, with a total dosing period of 16 weeks, taking into account the drug titration cycle.

In summary, the primary efficacy endpoint of this study is set as the percentage change in weight from baseline after 16 weeks of treatment.

## 3.5 Randomization and Blinding

### 3.5.1 Randomization

This study applies a block randomization method. An independent statistician, who is not involved in this trial, will generate a randomization code table using SAS version 9.4 or higher statistical analysis software. The random data will be reproducible, and the initial seed parameters for setting the random numbers will be preserved. The randomization code table will be loaded into the centralized randomization system (IWRS), which will randomly assign each subject to either the treatment group or the placebo group.

Participants will be assigned a "screening number" based on the order in which they sign the informed consent form. The screening number consists of five Arabic digits: the first two digits represent the center number, while the last three digits represent the screening sequence number. For example, a screening number of 01001 indicates the first participant at center 01 who signed the informed consent form. Each participant will be identified using a unique screening number.

Once a participant has passed the screening process, they will be randomly assigned a random number according to the order of enrollment, with multiple centers competing for enrollment. The random number consists of four Arabic digits: the first digit represents the dose group number, and the remaining three digits represent the enrollment sequence number.

Participants will be randomly assigned in a 3:1 ratio to the study drug group or the placebo group, receiving either VCT220 tablets or a placebo treatment. The specific groups and sample sizes for the study are as follows: 80 mg Dose Group (N=60)、80 mg Dose-Matched Placebo Group (N=20)、120 mg Dose Group (N=60)、120 mg Dose-Matched Placebo Group (N=20)、160 mg Dose Fast titration Group (N=30)、160 mg Dose Fast titration Matched Placebo Group (N=10)、 160 mg Dose Slow Titration Group (N=30)、160 mg Dose Slow Titration Matched Placebo Group (N=10) This design allows for the evaluation of the efficacy and safety of VCT220 across different dosing regimens compared to placebo.

The IWRS will not publicly disclose group allocation information. For participants who have been randomly assigned to a group but withdraw or exit the clinical trial for any reason, their random number will be retained regardless of whether they have received the study drug. Additionally, participants who exit the study will not be permitted to re-enroll in this trial.

### 3.5.2 Blinding Method

The study adopts a double-blind design, with the study drug provided by the sponsor or its designated unit, ensuring that the trial drug and the control drug are consistent in terms of packaging appearance, smell, and color. The principal investigator uses IWRS for randomizing the participants. The randomization table will be imported into IWRS, and the unblinding information will be exported after unblinding. The on-site blinding of the drugs will involve a random statistician and personnel from the sponsor unrelated to this trial, who will label and number the investigational products (including the study drug and placebo) based on the generated random allocation table. The blinding process will be documented and preserved in a blinding record file.

### 3.5.3 Emergency Unblinding

In the event of an emergency situation during the study (such as a Serious Adverse Event), it is essential to know the specific treatment that the subject has received. Authorized investigator may conduct emergency unblinding of the relevant subject through the Interactive Web Response System (IWRS) after obtaining consent. Notify the lead organization and clinical monitor within 24 hours after the emergency unblinding. All case data should be kept complete. Once an emergency unblinding occurs, the subject must discontinue treatment but will still need to receive follow-up care.

### 3.5.4 Unblinding Regulations

Any documents related to the research drug group information will be stored separately in a restricted access location until database lock. The project statistician will request the unblinding of subjects, which will be completed upon approval from the sponsor.

This study will conduct overall unblinding after the database is locked, revealing the group corresponding to the randomization numbers for statistical analysis of all data after grouping. The unblinding documents will be jointly signed by the principal investigator, sponsor, and statistical personnel.

# 4. Study Subjects

## 4.1 Inclusion Criteria

1) Aged 18 to 75 years (inclusive of 18 and 75 years) at the time of signing the informed consent form, with no restrictions on gender;

2) Body Mass Index (BMI) ≥ 28 kg/m² with or without comorbidities; or BMI ≥ 24 kg/m² and < 28 kg/m² with at least one of the following comorbidities: prediabetes (impaired fasting glucose and/or impaired glucose tolerance), hypertension, dyslipidemia, fatty liver, or obstructive sleep apnea due to overweight (specific definitions can be found in Appendix 1).

3) Participants must have undergone a controlled diet and exercise regimen for at least 12 weeks prior to screening, with stable weight during the 12 weeks prior to screening (weight change < 5%, where weight change = [maximum weight - minimum weight] / maximum weight; based on self-reported data);

4) Participants do not fertility plan during the study period and for 90 days after the study, are willing to implement effective contraceptive measures, and have no plans to donate sperm or eggs;

5) Willing and able to maintain a stable diet and exercise lifestyle during the study period, and timely record in the diary card;

6) Able to understand the procedures and methods of the study, willing to adhere strictly to the clinical study protocol to complete the research, and voluntarily sign the informed consent form.

## 4.2 Exclusion Criteria

1）Previously diagnosed with endocrine diseases or obesity caused by monogenic mutations, including but not limited to hypothalamic obesity, pituitary obesity, hypothyroid obesity, Cushing's syndrome, pancreatic neuroendocrine tumors, acromegaly, or gonadal dysfunction;
2）Previously underwent weight loss surgery or treatment (excluding acupuncture, liposuction, and abdominal liposuction if more than 1 year prior to screening), or plans to undergo surgical treatment for obesity during the study, such as gastric bypass or gastric banding;
3）Currently on a weight loss plan at the time of screening and not in a maintenance phase (based on self-reported data);
4）Previously diagnosed with type 1 diabetes, type 2 diabetes, or other specific types of diabetes;
5）Previously used any GLP-1 receptor agonists (GLP-1RA), multi-target agonists related to GLP-1 (such as GLP-1/glucose-dependent insulinotropic polypeptide [GIP] dual agonists, GLP-1/glucagon receptor [GCGR] dual agonists, etc.), or combination formulations containing GLP-1 agonists;
6）In the 12 weeks prior to screening, used any of the following medications or treatments:

- Dipeptidyl peptidase-4 (DPP-4) inhibitors, sodium-glucose cotransporter-2 (SGLT-2) inhibitors, metformin, insulin secretagogues, thiazolidinediones (TZD), or oral or injected hypoglycemic medications;
- Any approved or unapproved weight loss medications (e.g., Orlistat, Lorcaserin, Phentermine/topiramate, Naltrexone/bupropion, etc.) or herbal remedies, dietary supplements, meal replacements, etc., that affect weight;
- Used medications that might cause significant weight changes, including systemic glucocorticoids for more than 1 week; tricyclic antidepressants; antipsychotic or anticonvulsant medications (e.g., Mirtazapine, Amitriptyline, Mirabegron, Paroxetine, Phenelzine, Chlorpromazine, Clozapine, Olanzapine, Valproic acid and its derivatives, lithium preparations, etc.);

7）Meets any of the following cardiac function-related criteria:

- Meets any of the following cardiac function-related criteria:
- History of myocardial infarction, coronary angioplasty or bypass surgery, valvular heart disease or valvular repair, clinically significant unstable arrhythmias, unstable angina, transient ischemic attacks, or cerebrovascular accidents within the 6 months prior to screening;
- Congestive heart failure classified as NYHA (New York Heart Association) Class III or IV;
- Severe arrhythmias requiring treatment (e.g., Long QT syndrome) that, in the investigator's assessment, are not suitable for participation in this clinical study;
- Hypertension that was not effectively controlled prior to screening (i.e., systolic blood pressure ≥ 160 mmHg or diastolic blood pressure ≥ 100 mmHg);
- Clinical relevant abnormalities on the electrocardiogram (ECG) at the time of screening that could affect participant safety or interpretation of study results (e.g., QTcF > 450 msec, complete left bundle branch block, acute or chronic myocardial infarction signs of unknown origin, ST segment changes indicative of myocardial ischemia, second or third-degree atrioventricular block, severe bradyarrhythmias or tachyarrhythmias, etc.);
- Resting heart rate > 100 beats per minute;
- Other cardiac function abnormalities deemed significant and clinically relevant by the investigator.

8)Currently experiencing thyroid dysfunction that cannot be controlled with stable medication dosages, or with clinically significant abnormalities in thyroid function tests at the time of screening;

9）A history of medullary thyroid carcinoma, multiple endocrine neoplasia (MEN) type 2A or 2B syndrome, or relevant family history (defined as having first-degree relatives affected) ;

10）Diagnosed with malignant tumors within the last 5 years prior to screening (except for cured basal cell carcinoma of the skin or cervical carcinoma in situ);

11）A history of acute or chronic pancreatitis, symptomatic gallbladder disease (such as multiple gallstones), a history of pancreatic injury, or other high-risk factors that may lead to pancreatitis;

12）Clinically significant gastric emptying abnormalities (such as gastric outlet obstruction), severe chronic gastrointestinal diseases (such as active ulcers that occurred within the last 6 months), long-term use of medications that directly affect gastrointestinal motility (including but not limited to mosapride and cisapride), or having undergone gastrointestinal surgery (except for polypectomy and appendectomy) in the past 6 months as evaluated by the investigator who deems them unsuitable for participation in this clinical study;

13）A history of organ transplantation or currently having severe autoimmune diseases;

14）A history of major surgeries within the last 6 months, or plans to undergo surgeries that may affect study completion or compliance;

15）A history of moderate to severe depression or anxiety, or a history of severe mental illness, or a score of ≥ 15 on the depression screening scale (PHQ-9) during screening.

16）Previous suicidal tendencies or suicidal behavior

17）Any laboratory test indicator meets one of the following criteria during screening:

- Glycated hemoglobin (HbA1c) ≥ 6.5%;
- Fasting plasma glucose (FPG) ≥ 7.0 mmol/L;
- Estimated glomerular filtration rate (eGFR) calculated using the CKD-EPI formula < 60 mL/min/1.73m
- Alanine aminotransferase (ALT) and/or aspartate aminotransferase (AST) > 3.0× upper limit of normal (ULN);
- Total bilirubin (TBIL) > 1.5× ULN;
- Serum amylase or lipase > 1.5× ULN;
- Fasting triglycerides (TG) > 5.7 mmol/L;
- Fasting C-peptide < 0.8 ng/mL (or < 0.26 nmol/L);
- Calcitonin ≥ 1.0× ULN;
- Hemoglobin (Hb) < 110 g/L (male) or < 100 g/L (female);

18）During screening, those who test positive for hepatitis B virus surface antigen (HBsAg) (excluded if the quantitative test result for hepatitis B virus deoxyribonucleic acid [HBV-DNA] is below the lower limit of detection and no antiviral medication for hepatitis B has been used) or are currently undergoing antiviral treatment for hepatitis B, or those who test positive for hepatitis C virus (HCV) antibodies, or human immunodeficiency virus (HIV) antibodies, or syphilis borelia antibodies (Tp-Ab);
19）Known allergies to GLP-1 receptor agonist medications or related excipients;
20）Those who have previously participated in weight loss clinical trials; those who have participated as subjects in any drug or medical device clinical trial (excluding weight loss clinical trials) within 12 weeks prior to screening, or whose last drug administration in a clinical trial was less than five half-lives of that study drug before screening (the longer of the two times shall prevail) (no such restriction for screening failures);
21）Those who donated blood or experienced blood loss of ≥ 400 mL within 12 weeks prior to screening, or received a blood transfusion;
22）Known or suspected history of alcohol, tobacco addiction, or substance abuse at the time of screening;
23）Women who are pregnant or breastfeeding;
24）Positive blood pregnancy test results at the time of screening or before randomization (only applicable to women of childbearing potential);
25）Other conditions deemed unsuitable for participation in this study by the investigator.

## 4.3 Screening Failure

### 4.3.1 Review of Screening Period Examination Items

Subjects must sign an informed consent form before undergoing any study-related procedures, after which they will enter the screening period. During this time, the eligibility of the subjects will be reviewed, and various examinations will be completed, including vital signs and other assessments. If the following vital signs/examinations during the screening period (systolic blood pressure, diastolic blood pressure, fasting plasma glucose [FPG], fasting triglycerides [TG], ALT, AST, 12-lead ECG) meet the exclusion criteria, the investigator may determine that these indicators can be re-evaluated within a maximum of 2 weeks during the screening period for that subject. The investigator must document the items reviewed and the reasons for the re-evaluation. Each subject can only have each vital sign/examination re-evaluated once (except for cases where the QTcF on the screening ECG exceeds 450 msec or the QRS exceeds 120 msec, as detailed in "7.1.6 12-lead Electrocardiogram"). The eligibility review of the subjects will be based on the results of the re-evaluation.

### 4.3.2 Re-Screening of Subjects

Subjects who have failed screening must be registered as screening failures in the screening registration system. However, subjects who have failed screening may be re-screened at the discretion of the investigator. Based on the investigator's judgment and with careful consideration of the subject's safety, subjects who do not meet the inclusion criteria or who meet the exclusion criteria may be allowed to undergo re-screening at least 2 weeks after the initial confirmation of screening failure (the reason for re-screening must be documented in the medical record).Re-screened subjects will be considered as new subjects and must re-sign the informed consent form, receive a new screening number, and complete the screening visit process. Each subject is allowed to be re-screened only once.

# 5. Study Drug

## 5.1 Study Drug Information

**Investigational Drug**

| Name | VCT220 Tablets |
| --- | --- |
| Specification | Tablets, 10 mg/tablet, 50 mg/tablet |
| Drug Supplier | Vincentage Pharma Co., Ltd. |
| Drug Storage Conditions | Storage at Room Temperature |

**Reference Drug**

| Name | VCT220 Placebo |
| --- | --- |
| Specification | Tablets |
| Drug Supplier | Vincentage Pharma Co., Ltd. |
| Drug Storage Conditions | Storage at Room Temperature |

The appearance of the placebo (shape, color, size, packaging), as well as its odor, is identical to that of the VCT220 tablet and does not contain any active ingredients.

## 5.2 Study Drug Treatment

### 5.2.1 Dosing Regimen

In this study, both VCT220 tablets and the placebo (VCT220 placebo tablets) are administered orally, once daily, with meals, preferably taken with breakfast. The quantity and dosage of the medication for each dosage group during the treatment period are as follows:

- **Participants in the 80 mg dosage group or their placebo group:**

| Treatment Weeks | Total Dose per Administration | Method of Administration | | Total Number of Tablets per Administration |
| --- | --- | --- | --- | --- |
|  |  | 10 mg per tablet | 50 mg per tablet |  |
| W1 | 20mg | 2 | 0 | 2 |
| W2 | 40mg | 4 | 0 | 4 |
| W3-W16 | 80mg | 3 | 1 | 4 |

- **Participants in the120 mg dosage group or their placebo group:**

| Treatment Weeks | Total Dose per Administration | Method of Administration | | Total Number of Tablets per Administration |
| --- | --- | --- | --- | --- |
|  |  | 10 mg per tablet | 50 mg per tablet |  |
| W1 | 20mg | 2 | 0 | 2 |
| W2 | 40mg | 4 | 0 | 4 |
| W3 | 60mg | 1 | 1 | 2 |
| W4 | 80mg | 3 | 1 | 4 |
| W5-W16 | 120mg | 2 | 2 | 4 |

- **Subjects in the 160 mg dose fast titration group or its placebo group:**

| Treatment Weeks | Total Dose per Administration | Method of Administration | | Total Number of Tablets per Administration |
| --- | --- | --- | --- | --- |
|  |  | 10 mg per tablet | 50 mg per tablet |  |
| W1 | 20mg | 2 | 0 | 2 |
| W2 | 40mg | 4 | 0 | 4 |
| W3 | 80mg | 3 | 1 | 4 |
| W4 | 120mg | 2 | 2 | 4 |
| W5-W16 | 160mg | 1 | 3 | 4 |

- **Subjects in the 160 mg dose slow titration group or its placebo group:**

| Treatment Weeks | Total Dose per Administration | Method of Administration | | Total Number of Tablets per Administration |
| --- | --- | --- | --- | --- |
|  |  | 10 mg per tablet | 50 mg per tablet |  |
| W1-W2 | 20mg | 2 | 0 | 2 |
| W3-W4 | 40mg | 4 | 0 | 4 |
| W5 | 80mg | 3 | 1 | 4 |
| W6 | 120mg | 2 | 2 | 4 |
| W7-W16 | 160mg | 1 | 3 | 4 |

### 5.2.2 Dose Adjustment Scheme

If a subject experiences intolerable gastrointestinal adverse reactions during the dose titration phase, and the investigator determines that the current treatment regimen cannot be continued, treatment should be terminated. Dose adjustments for the subjects' medication are not permitted in the study.

## 5.3 Drug Management

### 5.3.1 Drug Packaging and Labeling

The sponsor is responsible for the packaging and labeling of the investigational drug.

The label content includes: drug name, drug number, specifications and packaging, dosage and administration, production batch number, expiration date, storage requirements, drug provider, precautions, etc., and should indicate "For clinical research use only." Except for the drug number, the packaging should not have any sensory distinguishability. The packaging and labeling of the investigational drug must comply with Good Manufacturing Practice (GMP) standards.

### 5.3.2 Drug Transportation, Storage, and Distribution

The sponsor establishes procedures for the supply and management of the investigational drug, including the receipt, storage, distribution, use, and disposal of the investigational drug. The investigational drug will be uniformly distributed by the sponsor, with a designated person directly delivering the drug to the research center. The research center should establish a complete drug receipt procedure, with a designated person responsible for receiving the investigational drug and signing the drug receipt form.

The research center should establish a strict drug management system according to the management procedures (designated personnel, dedicated storage, locked storage) responsible for the custody and distribution of the investigational drug, and establish a registration system (recording details including date, quantity, batch/serial number, expiration date, allocation code, signature, etc.). The research center should ensure that the storage environmental conditions (such as temperature) for the investigational drug comply with regulations and maintain records. If the storage conditions exceed the specified range, the research center must immediately contact the sponsor. Investigational drugs that have been improperly stored should not be distributed to any subjects until the sponsor has re-evaluated and approved their use. The research center's drug management personnel will distribute the investigational drugs to the research nurses based on the subjects' grouping information, and the research nurses will then distribute the investigational drugs to the subjects. Each issuance and return of the drug should be promptly recorded on a dedicated record form.

Regular inventory checks of the investigational drugs should be conducted. In the event of any loss, spillage, or misuse of the investigational drugs, detailed records must be made. After the study concludes, all investigational drugs transported to the research center (including all unused, partially used investigational drugs, and all returned empty drug boxes, along with a complete and signed drug return record) must be returned to the sponsor. The sponsor is responsible for the collection and destruction of these drugs, and they must not enter the market.

## 5.4 Concomitant Treatment

### 5.4.1 Allowed Combination Therapy

1）During the study period, participants may continue to take their original antihypertensive and lipid-lowering medications, but efforts should be made to keep the original drug types and dosages unchanged. Adjustments to the medication regimen are only allowed during the study period if the investigator deems it necessary to provide adequate supportive treatment.
2）If a participant experiences an adverse event (AE) during the study that requires treatment, symptomatic medications may be administered (provided that these medications are not expected to interfere with the assessment of the efficacy and safety of the investigational drug, or are drugs/treatments that have no interaction with the investigational drug).
3）Medications for treating underlying diseases (excluding medications that are prohibited for combination therapy).

### 5.4.2 Prohibited Combination Therapy

1）Any approved or unapproved weight loss medications (e.g., liraglutide, semaglutide, benaglutide, orlistat, chlorphentermine, phentermine/topiramate, naltrexone/bupropion, etc.), as well as herbal remedies, dietary supplements, meal replacements, or other products that may affect body weight.
2）Any antidiabetic medications (e.g., GLP-1 single-target or multi-target agonists, combination formulations containing GLP-1RA, DPP-4 inhibitors, SGLT-2 inhibitors, insulin, metformin, etc.).
3）Any treatments that may lead to weight instability, including both weight loss and weight gain medications, such as systemic corticosteroids used for more than one week; tricyclic antidepressants; antipsychotic or anticonvulsant medications (e.g., mirtazapine, amitriptyline, nortriptyline, paroxetine, phenelzine, chlorpromazine, clozapine, olanzapine, valproic acid and its derivatives, lithium preparations, methyldopa, etc.).
4）Weight loss surgeries/non-pharmacological treatments: surgical procedures for gastric reduction, liposuction, etc.; traditional Chinese medicine weight loss treatments (acupuncture, moxibustion, massage, cupping, etc.).

# 5.5 Treatment compliance

For participants who are followed up during the study period, "expected dosage" refers to the total amount of a specific medication that the participant is scheduled to take during a certain phase of the study. For participants who withdraw from the study early, "expected dosage" refers to the amount of medication that should have been taken from the start of that study phase until the last day the study medication was administered in that phase.

For each participant, the following formula will be used to calculate medicationcompliance:

Medication compliance

$$=\frac{Actual Medication Intake of Participants}{Total Expected Medication Dosage for Participants Throughout the Treatment Period}\times100\%$$

During the study period, the medication usage of participants will be accurately recorded. investigator must check the participants' medication usage, and any unused medication must be returned and verified.

Investigator should thoroughly document the date, time, actual dosage, and administration schedule of each medication received by participants in the electronic case report form (eCRF) to assess the participants' medication usage. The reasons for any interruption or reduction in medication must also be recorded in the eCRF.

# 6. Stopping Treatment and Participant Withdrawal

1) Poor compliance by the participant affects the assessment of efficacy/safety, and communication with the researcher has been ineffective.
2) The participant experiences an AE or SAE, and the researcher determines that treatment should be terminated.
3) The researcher believes that continuing participation in the clinical study may be harmful to the participant's health.
4) The participant experiences intolerable gastrointestinal adverse reactions, and the researcher judges that treatment should be stopped.
5) Pregnancy.
6) Serious protocol violations that may affect the study results.
7) The participant or their legal representative withdraws informed consent.
8) The participant lost to follow-up.
9) The participant passes away.
10) Other reasons.

Participants may choose to withdraw from the clinical study at any time. If the participant or their legal representative withdraws informed consent, the participant must exit the study and will no longer receive study treatment or continue follow-up at the scheduled visit time points outlined in the protocol.

For each participant who stops receiving the study medication, the researcher must document all required details in the electronic case report form (eCRF), including the date of treatment cessation and the reason. For all participants who discontinue the medication due to adverse events (AEs) or laboratory abnormalities, follow-up must be conducted until the symptoms (or laboratory values) return to the state prior to the start of the clinical study or to a level without medical issues. The results of this follow-up should be recorded in the eCRF (and the follow-up results will be documented in the original files of the study site after database lock).

Participants who discontinue the study medication early should, if possible, continue to follow the visit schedule and attend follow-up at the study site. If necessary, to keep the participant in the study for observation, telephone contact may be used as a substitute for the study site visit after the study medication has been discontinued. The decision to provide other treatments for participants who have stopped the study medication should be made at the discretion of the researcher.

# 7. Research Process

## 7.1 Checks and Assessments During the Study Period

### 7.1.1 Informed Consent

Investigator explains all research procedures to the participants before screening and obtain a voluntarily signed informed consent form from the participants (or their guardians). If any significant changes occur that affect the conduct of the research, participants will be required to sign an updated informed consent form.

For participants who are re-screened, a new informed consent form must be signed.

### 7.1.2 Collection of Demographic Data

During the screening period, demographic data of participants will be collected, including date of birth, gender, and ethnic information.

### 7.1.3 Inquiry/Documentation of Medical History and Personal History

Target Disease: Collection of Medical History Related to Obesity/Overweight Collect the medical history of participants regarding obesity/overweight, including associated conditions such as prediabetes, hypertension, dyslipidemia, fatty liver disease, and obstructive sleep apnea syndrome caused by overweight. The diagnosis dates and treatment history of these comorbidities should be recorded.

Other Medical History：During the screening period, document any significant medical history prior to signing the informed consent form, including the start and end dates of diseases. Inquire about any history of malignant tumors, acute or chronic pancreatitis, severe gastrointestinal diseases, severe cardiovascular diseases, heart dysfunction, and mental health history.

During the screening period, it is also necessary to obtain information on the participant's allergy history, drug dependence history, alcohol consumption status, smoking history, and clinical trial history. The researcher will verify the inclusion/exclusion criteria for participants based on their medical and personal history.

### 7.1.4 Previous Treatment History and concomitant reatment

Treatment of Target Disease：During the screening period, document the participant's complete history of GLP-1 receptor agonist (GLP-1RA) treatments. Record any oral and injectable antidiabetic medications used in the 12 weeks prior to signing the informed consent form (excluding GLP-1RA medications). Additionally, note any obesity or weight-loss surgeries that the participant underwent in the year prior to screening, as well as any other medications or non-medication treatments that may affect weight.

History of Treatment for Other Diseases：Document any other medication treatments (including prescription drugs, over-the-counter medications, herbal remedies, etc.) or non-medication treatments (including surgical history) that the participant received in the 12 weeks prior to screening.

Collection of concomitant Treatments：Collect information on any medications or non-medication treatments received by the participant from the time of signing the informed consent form until the end of the safety follow-up period. The collection ofconcomitant treatments should include: Start and end dates of the treatment、Dosage、Treatment frequency、Route of administration or treatment method、 Indications for treatment This information should be recorded in the eCRF (electronic Case Report Form) on theconcomitant treatment page.

The researcher will verify the participant's inclusion/exclusion criteria based on their treatment history.

### 7.1.5 Vital Signs and Physical Examination

**Vital Signs**

The vital signs examination includes blood pressure (mmHg), temperature (℃), and pulse (beats/min). The measurement methods for systolic and diastolic blood pressure must adhere to the standard clinical practice guidelines of the research center. Participants should rest for at least 5 minutes before measuring blood pressure. Caffeine intake, smoking, and exercise should be avoided for at least 30 minutes prior to the measurement.

For subsequent measurements, it is recommended to use the same arm as in Visit 1. The investigator will verify the participant's inclusion/exclusion criteria based on the examination results.

**Physical Examination**

The physical examination includes a comprehensive physical examination and a symptom-oriented physical examination

The comprehensive physical examination covers the following areas: skin/mucous membranes, lymph nodes, head and neck (including the thyroid), chest (heart and lungs), abdomen (liver, gallbladder, spleen, kidneys), musculoskeletal system, and nervous system. This examination is conducted during the screening period, at the first and last visits of the treatment period, during safety follow-up visits, and at early termination visits. For other visits during the treatment period, the investigator may perform a symptom-oriented physical examination based on the clinical symptoms presented by the participant.

### 7.1.6 12 Lead Electrocardiogram (ECG)

The 12-lead electrocardiogram (12-ECG) requires the subject to rest for at least 5 minutes before the examination. Record the heart rate, PR interval, RR interval, QRS duration, QT interval, QTcF (Fredericka formula: QTcF=QT/RR^0.33^), and the 12-ECG diagnostic results.

If the QTcF exceeds 450 msec or the QRS duration exceeds 120 msec during the screening period, the ECG should be repeated twice (on the same day of screening, with at least a 1-minute interval between each measurement), and the average of the three QTcF or QRS values should be used to determine the subject's eligibility. If the 12-lead ECG examination during the screening period is within 7 days of D1, the above tests do not need to be repeated, and the screening results will be considered as the baseline.

### 7.1.7 Laboratory Tests

During the study period, the following laboratory tests will be conducted by the local laboratories at each center. If any tests cannot be performed by the local laboratory, they may be sent to the central laboratory for analysis.

Blood biochemistry tests must be conducted in a fasting state prior to administration of the medication.

If the subject's complete blood count, urinalysis, blood biochemistry, blood lipase, and blood amylase tests during the screening period are conducted within 7 days prior to D1, these tests do not need to be repeated on D1. The results from the screening period will be considered as the baseline. However, any abnormal results from the screening period must be retested and will then be used as the baseline for this study.

| **Project** | **Examination Indicators** |
| --- | --- |
| Complete Blood Count (CBC) | White Blood Cell Count (WBC) ,Red Blood Cell Count (RBC) ,Hemoglobin (Hb) ,Platelet Count (PLT), Hematocrit (HCT) ,Neutrophil Count (NEUT#), Neutrophil Percentage (NEUT%) ,Lymphocyte Count (LYMPH#) ,Lymphocyte Percentage (LYMPH%) Eosinophil Count (EO#) Eosinophil Percentage (EO%) Basophil Count (BASO#), Basophil Percentage (BASO%),Monocyte Count (MONO#) ,Monocyte Percentage (MONO%). |
| Blood Biochemistry | Total Protein (TP), Albumin (ALB) Globulin (GLO), Total Bilirubin (TBIL) ,Direct Bilirubin (DBIL), Alanine Aminotransferase (ALT) ,Aspartate Aminotransferase (AST) ,Urea / Blood Urea Nitrogen (BUN), Creatinine (Cr), Uric Acid (UA), Lactate Dehydrogenase (LDH), Creatine Kinase (CK) ,Creatine Kinase Isoenzyme (CK-MB), Alkaline Phosphatase (ALP), Gamma-Glutamyl Transferase (GGT) ,Potassium (K) Sodium (Na) Chloride (Cl) ,Magnesium (Mg), Calcium (Ca) ,Phosphorus (P). |
| Urinalysis | Urine pH, Urine Protein (PRO) ,Urine Ketones (KET), Urine Specific Gravity (SG) ,Urine Occult Blood (BLD) ,Urine Glucose (GLU), Urine Urobilinogen (URO) ,Urine Bilirubin (BIL) ,Urine Red Blood Cells Urine ,White Blood Cells. |
| Lipase, Amylase | Lipase, Amylase. |
| Thyroid Function Tests | Thyroid-Stimulating Hormone (TSH), Free Thyroxine (FT4), Free Triiodothyronine (FT3). |
| Coagulation Function Tests | International Normalized Ratio (INR) Activated Partial Thromboplastin Time (APTT) Prothrombin Time (PT) |
| Calcitonin | Calcitonin |
| Infection Panel | Hepatitis B Virus Surface Antigen (HBsAg) , Hepatitis C Virus (HCV), Antibody Human Immunodeficiency Virus (HIV) Antibody ,Treponema Pallidum Antibody (Tp-Ab) Test If HBsAg is positive, a quantitative test for Hepatitis B Virus Deoxyribonucleic Acid (HBV-DNA) is required. |

### 7.1.8 Blood Pregnancy Test and Contraceptive Requirements

Female have childbearing potential must undergo the corresponding blood pregnancy tests according to the study protocol. All blood pregnancy tests during the study will be conducted by the local laboratories of each center. If, at any time during the study, a female participant reports the absence of menstruation or irregular menstrual cycles, a blood pregnancy test should be performed immediately to rule out or confirm pregnancy. Once a positive blood pregnancy test is obtained, the study medication must be discontinued immediately.

In this study, female participants who have been menopausal for one year or more, or who have undergone permanent sterilization (such as tubal ligation, hysterectomy, or bilateral salpingectomy), are considered to be of non-childbearing potential, and blood pregnancy tests are not required during the study.

Acceptable contraceptive methods in this study include: bilateral tubal ligation, male sterilization, correct use of hormonal contraceptives that suppress ovulation, hormone-releasing intrauterine devices (IUDs), copper intrauterine devices (IUDs), and condoms.

### 7.1.9 Scales

During the screening period, the Patient Health Questionnaire-9 (PHQ-9) will be used to assess for depression. The scale template can be found in Appendix 3.

### 7.1.10 Liver, Gallbladder, Pancreas, Spleen, and Bilateral Kidney Ultrasound

The examination areas for the ultrasound of the liver, gallbladder, pancreas, spleen, and bilateral kidneys include the liver, gallbladder, pancreas, spleen, and bilateral kidneys.

### 7.1.11 FPG, HbA1c, Fasting Insulin, and Fasting C-Peptide

The blood samples for FPG, HbA1c, fasting insulin, and fasting C-peptide should all be collected after fasting prior to medication administration.

The FPG, HbA1c, and fasting C-peptide during the screening period, as well as the FPG during the safety follow-up period, will be conducted by the local laboratories at each center. The FPG, HbA1c, fasting insulin, and fasting C-peptide during the treatment period and for early withdrawal visits will be uniformly tested by the central laboratory.

### 7.1.12 Fasting Lipid Profile

The lipid profile includes Total Cholesterol (TC), Triglycerides (TG), High-Density Lipoprotein Cholesterol (HDL-C), and Low-Density Lipoprotein Cholesterol (LDL-C). Blood samples must be collected after fasting prior to medication administration. The fasting lipid profile during the screening period will be conducted by the local laboratories at each center. The fasting lipid profile during the treatment period and for early withdrawal visits will be uniformly tested by the central laboratory.

### 7.1.13 Height, Weight, and Waist Circumference

Height is measured only during the screening period, recorded in centimeters (cm), and should be precise to one decimal place.

Weight is measured at each visit, recorded in kilograms (kg), and precise to one decimal place. All weight measurements are repeated twice.

Waist circumference is defined as the midpoint of the line connecting the anterior superior iliac spine and the lower edge of the costal arch at the midaxillary line on both sides. Waist circumference is measured at each visit, recorded in centimeters (cm), and precise to one decimal place. All waist circumference measurements are repeated twice.

The specific procedures for measuring height, weight, and waist circumference can be found in Appendix 2.

### 7.1.14 Collection of Biological Samples

During the entire study treatment period, PK blood samples will be collected from all participants. All participants are required to undergo sparse PK blood sample collection at the time points specified in the study protocol. Approximately 40 participants will undergo intensive PK blood sampling, including 10 participants from the 80 mg dose group (or its placebo group), 10 participants from the 120 mg dose group (or its placebo group), 10 participants from the 160 mg dose slow titration group (or its placebo group), and 10 participants from the 160 mg dose fast titration group (or its placebo group). The timing and volume of PK blood sample collection are detailed in the PK sampling schedule. All PK blood samples will be uniformly tested by a central laboratory.

Participants who require intensive PK blood sampling must be hospitalized during the corresponding visit (i.e., Visit 5). After completing the PK blood sampling 24 hours post-administration, participants may be discharged. During Visits 2 to 6, if participants have taken the study drug prior to arriving at the research center, PK samples can still be collected. It is essential to accurately record the administration and sampling times as precisely as possible.

For participants who require sparse PK blood sampling, during Visits 2 to 6, if they have taken the study drug prior to arriving at the research center, PK blood samples can still be collected. It is important to accurately record the actual administration time, dosage, and sampling time as precisely as possible.

In the event of SAE, if conditions permit, the investigator may choose to collect an unplanned PK sample for exploratory efficacy analysis. Additionally, in cases of early withdrawal, the investigator may also opt to collect an unplanned PK sample if conditions allow. To facilitate operations at the center, the planned PK blood sampling can be combined with the scheduled laboratory blood collection times during the same visit.

If a 12-lead ECG examination, vital signs assessment, and PK blood sample collection are scheduled for the same visit, efforts should be made to complete the 12-lead ECG and vital signs checks before the PK blood sample collection. This approach aims to maximize the accuracy of the blood sample collection.

After each venous blood sample collection, the exact time of sampling (accurate to the minute), as well as the actual administration time and dosage, must be accurately recorded in the eCRF for each sample. If any discrepancies arise due to technical difficulties that deviate from the above protocol, the details of the deviation must also be documented in the eCRF.

### 7.1.15 Adverse Events

The entire research process requires close monitoring of the participants' adverse events (AEs) until the investigator confirms that the AEs have resolved or are no longer clinically significant. The time frame for collecting AEs is detailed in the section on "Adverse Events and Serious Adverse Events."

### 7.1.16 Dietary and Exercise Guidance

Reference Appendix 8: Dietary and Exercise Guidance During the study period, the investigator will continuously provide health education and dietary and exercise guidance to the participants. The investigator should carefully inquire about and understand the participants' lifestyle conditions. If any unreasonable lifestyle habits are encountered (such as binge eating during holidays), appropriate guidance should be provided in a timely manner.

## 7.2 Visit Schedule

### 7.2.1 Screening Period (D-14 to D-1)

Before participating in the screening, subjects Voluntary sign an informed consent form. Only those who meet all inclusion criteria and do not meet any exclusion criteria are eligible to participate in the study.

- Sign the informed consent form
- Collect demographic data
- Record medical history
- Record previous treatment history
- Vital signs
- Complete physical examination
- 12-lead ECG examination
- Laboratory tests: complete blood count, blood biochemistry, urinalysis, serum amylase and lipase, thyroid function, coagulation function, calcitonin, and infection panel
- Blood pregnancy test (only for women of childbearing potential)
- PHQ-9 scale
- Ultrasound of liver, gallbladder, pancreas, spleen, and both kidneys
- Measure height, fasting weight, and waist circumference
- Fasting plasma glucose (FPG), HbA1c, fasting C-peptide
- Fasting lipid profile
- Confirm inclusion/exclusion criteria
- Record concomitant treatments
- Provide dietary and exercise guidance

### 7.2.2 Treatment Period

**D1（V1）**

If the 12-lead ECG, complete blood count, urinalysis, blood biochemistry, serum lipase, and serum amylase tests conducted during the screening period are within 7 days prior to D1, these tests do not need to be repeated, and the screening results can be used as baseline data. Any abnormal results from the screening period must be retested and confirmed to be included as the baseline for this study. The first dose of the treatment will be administered only after all assessments on D1 are completed.

- Vital signs
- Complete physical examination
- 12-lead ECG examination
- Laboratory tests: complete blood count, blood biochemistry, urinalysis, serum amylase, and serum lipase
- Blood pregnancy test (only for women of childbearing potential)
- Measure fasting weight and waist circumference
- Fasting plasma glucose (FPG), HbA1c, fasting insulin, fasting C-peptide
- Fasting lipid profile
- Pharmacokinetic (PK) sample collection
- Distribute medication and diary cards
- Record adverse events (AE)
- Record concomitant treatments
- Provide dietary and exercise guidance
- Reassess inclusion/exclusion criteria
- Randomization

**D15 ± 3 Days (V2)**

- Vital signs
- Symptom-oriented physical examination
- 12-lead ECG examination
- Laboratory tests: complete blood count, blood biochemistry, urinalysis, serum amylase, and serum lipase
- Measure fasting weight and waist circumference
- Fasting plasma glucose (FPG)
- Pharmacokinetic (PK) sample collection
- Collect medication and diary cards
- Distribute medication and diary cards
- Record adverse events (AE)
- Record concomitant treatments
- Provide dietary and exercise guidance

**D29 ± 3 Days (V3)**

- Vital signs
- Symptom-oriented physical examination
- 12-lead ECG examination
- Laboratory tests: complete blood count, blood biochemistry, urinalysis, serum amylase, serum lipase, thyroid function, coagulation function, calcitonin
- Blood pregnancy test (only for women of childbearing potential)
- Measure fasting weight and waist circumference
- Fasting plasma glucose (FPG), HbA1c, fasting insulin, fasting C-peptide
- Fasting lipid profile
- Pharmacokinetic (PK) sample collection
- Collect medication and diary cards
- Distribute medication and diary cards
- Record adverse events (AE)
- Record concomitant treatments
- Provide dietary and exercise guidance

**D43 ± 3 Days (V4)**

- Vital signs
- Symptom-oriented physical examination
- 12-lead ECG examination
- Laboratory tests: complete blood count, blood biochemistry, urinalysis, serum amylase, and serum lipase
- Measure fasting weight and waist circumference
- Fasting plasma glucose (FPG)
- Pharmacokinetic (PK) sample collection
- Collect medication and diary cards
- Distribute medication and diary cards
- Record adverse events (AE)
- Record concomitant treatments
- Provide dietary and exercise guidance

**D57 ± 3 Days (V5)**

Subjects requiring intensive PK blood sampling must be hospitalized on the day of the visit and may be discharged after all PK blood samples have been collected.

- Vital signs
- Symptom-oriented physical examination
- 12-lead ECG examination
- Laboratory tests: complete blood count, blood biochemistry, urinalysis, serum amylase, and serum lipase
- Measure fasting weight and waist circumference
- Fasting plasma glucose (FPG)
- Pharmacokinetic (PK) sample collection
- Collect medication and diary cards
- Distribute medication and diary cards
- Record adverse events (AE)
- Record concomitant treatments
- Provide dietary and exercise guidance

**D85 ± 3 Days (V6)**

- Vital signs
- Symptom-oriented physical examination
- 12-lead ECG examination
- Laboratory tests: complete blood count, blood biochemistry, urinalysis, serum amylase, and serum lipase
- Measure fasting weight and waist circumference
- Fasting plasma glucose (FPG)
- Pharmacokinetic (PK) sample collection
- Collect medication and diary cards
- Distribute medication and diary cards
- Record adverse events (AE)
- Record concomitant treatments
- Provide dietary and exercise guidance

**D113 + 3 Days (V7)**

- Vital signs
- Complete physical examination
- 12-lead ECG examination
- Laboratory tests: complete blood count, blood biochemistry, urinalysis, serum amylase, serum lipase, thyroid function, coagulation function, calcitonin
- Blood pregnancy test (only for female subjects of childbearing potential)
- Ultrasound of the liver, gallbladder, pancreas, spleen, and both kidneys
- Measure fasting weight and waist circumference
- Fasting plasma glucose (FPG), HbA1c, fasting insulin, fasting C-peptide
- Fasting lipid profile
- Collect medication and diary cards
- Record adverse events (AE)
- Record concomitant treatments
- Provide dietary and exercise guidance

### 7.2.3 Safety Follow-up Period

**D126 ± 7 Days (V8)**

- Vital signs
- Complete physical examination
- 12-lead ECG examination
- Laboratory tests: complete blood count, blood biochemistry, urinalysis, serum amylase, serum lipase, thyroid function, coagulation function, calcitonin
- Blood pregnancy test (only for female subjects of childbearing potential)
- Ultrasound of the liver, gallbladder, pancreas, spleen, and both kidneys
- Measure fasting weight and waist circumference
- Fasting plasma glucose (FPG)
- Record adverse events (AE)
- Record concomitant treatments
- Provide dietary and exercise guidance

### 7.2.4 Early Termination Visit

The early termination visit is only applicable to subjects who withdraw early during the treatment period and should be conducted within one week of learning about the subject's early withdrawal from the study. If a subject withdraws early during the safety follow-up period, the visit procedures should be the same as those for the safety follow-up period. For subjects who withdraw their informed consent, the early termination visit should be completed as much as possible. The following items need to be completed during the early termination visit:

- Vital signs
- Complete physical examination
- 12-lead ECG
- Laboratory tests: complete blood count, blood biochemistry, urinalysis, serum amylase and lipase, thyroid function, coagulation function, calcitonin
- Blood pregnancy test (only for women of childbearing potential)
- Measurement of fasting weight and waist circumference
- Ultrasound of the liver, gallbladder, pancreas, spleen, and both kidneys
- FPG, HbA1c, fasting insulin, fasting C-peptide
- Fasting lipid profile
- PK sample collection
- Collection of returned medication and diary cards
- Record of adverse events (AE)
- Record of concomitant treatments
- Dietary and exercise guidance

## 7.3 Subject Management

Subjects should visit the research center for their scheduled visits as notified in advance by the investigator. If any of the following requirements are not met, the visit should be rescheduled:

- Subjects must be in a fasting state (at least 8 hours fasting) during all routine clinical visits.
- Subjects should avoid high-fat and high-calorie diets the day before the research visit and avoid extensive exercise.
- Subjects must abstain from smoking, alcohol, and caffeine for 8 hours prior to the research visit.

Before each visit to the research center, the investigator must remind subjects to be fasting and not to use the study medication. On the day of the visit, after completing all fasting assessments required by the visit procedures at the research center, subjects may use the study medication as per the protocol requirements.

For subjects requiring intensive pharmacokinetic (PK) blood sampling, they should be admitted to the ward in a fasting state on the morning of the visit (or the night before). They may be discharged only after all PK blood samples have been collected.

The investigator should instruct subjects to avoid vigorous exercise, binge eating, staying up late, and other irregular lifestyles throughout the study period to prevent any potential impact on the evaluation of efficacy and safety indicators.

## 7.4 Drug Assay Analysis of Biological Samples

The PK sample testing will be conducted using validated procedures and methods outlined in the laboratory manual, as detailed in the relevant Standard Operating Procedures.

# 8 Evaluation Indicators

## 8.1 Efficacy Indicators

**Primary Endpoint Indicators**

- Percentage change in weight from baseline after 16 weeks of treatment.

**Secondary Endpoint Indicators**

- Proportion of participants with a weight loss of ≥5% from baseline after 16 weeks of treatment;
- Absolute change in weight from baseline after 16 weeks of treatment;
- Absolute change in BMI from baseline after 16 weeks of treatment;
- Absolute change in waist circumference from baseline after 16 weeks of treatment;
- Change in lipid profile (TG, TC, LDL-C, HDL-C) from baseline after 16 weeks of treatment;
- Change in blood glucose levels (FPG, HbA1c, fasting insulin, fasting C-peptide) from baseline after 16 weeks of treatment.

## 8.2 Safety Indicators

- Adverse Events (AE), including Treatment Emergent Adverse Events (TEAE) and Serious Adverse Events (SAE);
- Vital signs (sitting blood pressure [systolic and diastolic], pulse, temperature), physical examination, 12-lead ECG;
- Clinical laboratory tests (complete blood count, urinalysis, blood biochemistry, lipase and amylase, thyroid function, coagulation function, calcitonin).

## 8.3 Pharmacokinetic Indicators

- Pharmacokinetic Analysis: Peak concentration (Cmax), trough concentration (Cmin), time to peak concentration (Tmax), area under the concentration-time curve from 0 hours to the last measurable concentration (AUC0-last), area under the concentration-time curve within the dosing interval (AUC0-tau); if data permits, also include area under the concentration-time curve from 0 hours to infinity (AUC0-inf), apparent clearance (CL/F), apparent volume of distribution (Vz/F), and terminal elimination half-life (t1/2).
- Population Pharmacokinetic (PopPK) Analysis: Based on PopPK analysis methods, explore the pharmacokinetic characteristics of VCT220 tablets in overweight/obese subjects without diabetes.

# 9 Adverse Events and Serious Adverse Events

## 9.1Adverse Events

### 9.1.1 Definition of Adverse Events

AE: All adverse medical events that occur in subjects after receiving the study drug, which can manifest as symptoms, signs, diseases, or laboratory test abnormalities, but are not necessarily causally related to the study drug.

Abnormal laboratory test results that meet at least one of the following criteria may be considered for reporting the abnormal objective values as an AE:

- The test result is accompanied by symptoms;
- The test result requires further diagnostic testing or medical/surgical intervention;
- The test result leads to an adjustment in the study drug administration (beyond the dose adjustments specified in the protocol);
- The investigator or sponsor believes that the test result constitutes an AE.

When a subject signs the informed consent form, any concomitant diseases that are present during the screening period and do not worsen in severity or frequency during the study are defined as baseline medical conditions and are not considered AE or SAE. However, if there is an unexpected worsening of such concomitant diseases, the occurrence of complications, or an increase in frequency, then the worsening or complications should be recorded as an AE accordingly. The investigator should ensure that the terminology used to document the event accurately reflects the changes in the condition (e.g., "worsening of...").

TEAE is defined as any adverse event that occurs after the first administration of the study drug, or any adverse event that was present before the first administration but has increased in severity from the first administration to within 2 weeks after the last administration of the study drug.

In this study, weight loss resulting from the effects of the drug and lifestyle modifications does not need to be recorded as an AE. However, if a subject experiences significant weight loss (e.g., a decrease of more than 5%) due to other comorbid conditions, this should be recorded as an AE related to the underlying disease.

#### 9.1.1.1 Death

All deaths occurring during the study period or within the follow-up period after the last administration of the study drug, as specified in the study protocol, must be reported according to the following requirements:

- Deaths Due to Disease Progression: These should be reported as Serious Adverse Events (SAEs).
- Deaths of Unknown Cause: These should also be reported as SAEs, with the reporting term "Death of Unknown Cause." Further clarification regarding the cause of death should be provided during follow-up. Autopsy: An autopsy may assist in determining the cause of death. If an autopsy is performed, the autopsy report should be submitted to the sponsor's pharmacovigilance team or their authorized representative as soon as possible.

### 9.1.2 Collection and Recording of Adverse Events

All AEs/SAEs occurring from the time the subject first receives the study drug until the end of the safety follow-up period must be recorded, and the following information should be collected:

- AE Name
- Start and End Dates of AE
- Common Terminology Criteria for Adverse Events (CTCAE) Grade
- Whether it is an SAE
- Investigator's Assessment of Causality Between AE and Study Drug
- Actions Taken Regarding the Study Drug
- Treatment Measures Taken for AE
- Outcome
- Whether it Led to Withdrawal from the Study

In addition, the following information will be collected for each SAE:

- Date of SAE Occurrence
- Date Investigator Became Aware of SAE
- Reason for SAE Determination
- Date of Discharge (if applicable)
- Possible Cause of Death (if applicable)
- Date of Death (if applicable)
- Autopsy Results (if applicable)
- Causality Assessment with Study Drug
- Description of SAE

**Note：**The sponsor reserves the right to collect additional information regarding ongoing AEs/SAEs from any subject at the conclusion of the study, if necessary. Once a subject withdraws from the study, the investigator is no longer obligated to actively collect and report any new AEs or SAEs that occur after the safety follow-up period. However, if the investigator becomes aware of any SAE (including death) that occurs after the subject has withdrawn from the study, and there is reasonable cause to believe that the event may be related to the study drug, the investigator should report it to the sponsor's pharmacovigilance team or their authorized representative.

### 9.1.3 Criteria for Determining the Severity of Adverse Events

Use the Common Terminology Criteria for Adverse Events (CTCAE) version 5.0 from the National Cancer Institute to record the severity of adverse events (AEs), which are classified into grades 1 to 5. AEs not listed in the CTCAE version 5.0 table can be classified into grades 1 to 5 according to the standards in the table below.

| Grade | CTCAE Description |
| --- | --- |
| 1 | Mild: Asymptomatic or mild symptoms; clinical or diagnostic observation only; no intervention required. |
| 2 | Moderate: Requires minor, local, or non-invasive intervention; limitations in activities of daily living (ADL) comparable to age* |
| 3 | Severe or medically significant but not immediately life-threatening: Requires hospitalization or prolongation of hospitalization; disability, limitations in self-care ADL** |
| 4 | Life-threatening: Requires urgent intervention. |
| 5 | Death related to the adverse event. |
| CTCAE = Common Terminology Criteria for Adverse Events  *: Instrumental ADL refers to activities such as cooking, shopping, using the phone, managing finances, etc.  **: Self-care ADL refers to activities such as bathing, dressing and undressing, eating independently, personal hygiene, taking medication, and getting out of bed, without being bedridden.  Note the distinction between severe adverse events and serious adverse events. "Severe" is used to describe the intensity and does not necessarily indicate a Serious Adverse Event (SAE). For example, a headache may be classified as severe in terms of intensity but would not be considered an SAE unless it meets the SAE criteria. | |

### 9.1.4 Criteria for Assessing the Relationship Between Adverse Events and Investigational Drugs

The investigator will assess the causal relationship between the investigational drug and the adverse event as "definitely related," "probably related," "possibly related," "definitely not related," or "unable to determine."

The investigator analyzes the correlation between the adverse event and the investigational drug based on the established criteria, categorizing them into five classes: "related," "very likely related," "possibly related," "possibly not related," and "not related." Among these, "related," "very likely related," and "possibly related" are classified as "related," while "possibly not related" and "not related" are classified as "unrelated." AEs classified as "related" are counted as adverse drug reactions (ADRs). The investigator must document the ADRs using medical terminology.

**Criteria for Assessing the Relationship Between Adverse Events and Investigational Drugs ^[19]^:**

| Assessment Results  Basis for Assessment | Related | Likely Related | Possibly Related | | | | Possibly Unrelated | | Unrelated |
| --- | --- | --- | --- | --- | --- | --- | --- | --- | --- |
| Is there a reasonable temporal relationship | + | + | + | | | | + | | - |
| Does it align with known drug mechanisms, characteristics, or known adverse reactions? | + | + | + | | - | | + | - | - |
| De-challenge Results | + | + | + | - | + | - | - | | - |
| Re-challenge Results | + | - | - | | | | - | | - |
| Can other reasonable explanations account for it | - | - | + | - | -/+ | - | ++ | + | + |

Note:

+ indicates affirmation or a positive result;

- indicates negation, a negative result, or a situation where results are not yet obtained;

++ indicates that other "more" reasonable explanations can be provided.

### 9.1.5 Outcome of Adverse Events

Record the outcomes of Adverse Events in the eCRF as follows:

- Recovered
- Improved/Resolved
- No Improvement/Unresolved/Persistent
- Recovered with Sequelae
- Death
- Unknown

### 9.1.6 Follow-up of Adverse Events

All Adverse Events, including laboratory abnormalities, must be followed up until one of the following conditions is met:

1) The event resolves;
2) The event stabilizes;
3) The event returns to baseline levels;
4) The event can be attributed to a drug other than the study drug or is unrelated to the study procedures;
5) When no further information can be obtained (e.g., the subject refuses to provide more information, or there is evidence that maximum efforts have been made but the subject remains lost to follow-up).

## 9.2 Serious Adverse Events

### 9.2.1 Definition of Serious Adverse Events

SAE: An adverse medical event that occurs after a subject receives the investigational drug and meets any one or more of the following criteria should be considered an SAE:

1）Death;
2）Life-threatening condition (the occurrence of the adverse event poses an immediate risk of death to the subject, and does not refer to a hypothetical situation where the adverse event may develop into a serious condition in the future, such as drug-induced hepatitis without liver failure);
3）Permanent or severe disability or functional loss;
4）Requires hospitalization or prolongation of current hospitalization; if the subject had a pre-existing condition or disease prior to enrollment in the study, and hospitalization and/or surgery was planned before the study began or during the study without an unexpected worsening during the study, it does not qualify as an SAE, but must be clearly documented in the medical records;
5）Congenital anomalies or birth defects;
6）Other important medical events: These may not immediately threaten life, lead to death, or require hospitalization, but if medical intervention is needed to prevent any of the above situations from occurring, they are typically considered serious. For example, significant treatment in the emergency room or allergic bronchospasm occurring at home, cachexia or seizures that do not require hospitalization, and drug dependence or addiction.

**Note:**

- Hospitalization or prolongation of hospitalization also includes intra-hospital transfers to emergency or intensive care units (e.g., transfer from pediatrics to internal medicine, transfer from internal medicine to coronary care unit, transfer from neurology to tuberculosis ward, etc.).
- Diagnostic and therapeutic non-invasive and invasive procedures, such as surgeries, should not be reported as AE. However, if the condition being treated by the procedure meets the definition of an AE, it should be reported as such. For example, acute appendicitis occurring during the adverse event reporting period should be reported as an AE, while the appendectomy performed to treat that condition should be recorded as a treatment measure for that adverse event.

**The Following Situations Are Not Considered SAE:**

- Hospitalization or prolongation of hospitalization due to non-AE reasons/convenience (such as for medical insurance reimbursement purposes) or purely for the purpose of clinical research does not meet the criteria for a medical event and therefore is not considered an SAE.
- The following types of institutional admissions are not included in the criteria for SAE hospitalization:
- Rehabilitation facilities;
- Hospice care facilities;
- Short-term care facilities (e.g., caregiver support);
- Specialized nursing facilities;
- Nursing homes;
- Routine emergency department observation;
- Same-day surgeries (outpatient/day surgeries/day procedures).
- Hospitalizations or Prolonged Hospital Stays Due to the Following Reasons Are Not Considered SAEs:
- Hospitalization that is unrelated to the onset of a new AE or exacerbation of a pre-existing condition (e.g., conducting tests on persistent laboratory abnormalities present before treatment);
- Hospitalization for non-medical reasons (e.g., the subject being homeless);
- Administrative hospitalizations (e.g., annual physical examinations);
- Hospitalizations specified in the study protocol (e.g., for procedures required by the study protocol);
- Voluntary hospitalizations that do not lead to clinical adverse events (e.g., for elective cosmetic surgery);
- Treatments or surgeries that are pre-planned for the entire study or for individual subjects;
- Hospitalization specifically for the purpose of using the investigational drug.

### 9.2.2 Reporting and Handling of Serious Adverse Events

For all SAEs occurring during the clinical study, whether they are initial reports or follow-up reports, the investigator must immediately complete the Serious Adverse Event Report Form, sign and date it, and report it to the sponsor or authorized representative without delay (no later than 24 hours after becoming aware of the SAE). The investigator should ensure that all necessary information is submitted within the specified timeframe.

For all SAEs, the investigator is obligated to obtain and provide information to the sponsor within the reporting timeframe mentioned above. Additionally, the sponsor may request the investigator to quickly obtain more follow-up information. This information may be more detailed than what is reflected on the Serious Adverse Event Report Form. Overall, a sufficiently detailed description of the adverse event should be included to allow for a comprehensive medical assessment of the case and an independent evaluation of the likelihood of causality. Furthermore, the investigator must provide relevant information regarding other possible causes of the adverse event, such as concomitant medications and comorbidities.

In the event of a subject's death, a summary of the autopsy results (if available) must be submitted to the sponsor or their authorized representative as soon as possible.

For contact information regarding SAE reporting, please refer to the investigator documents provided to each center. The research center must properly retain the original SAE report form.

For more detailed information, please refer to the pharmacovigilance management plan.

## 9.3 Suspected Unexpected Serious Adverse Reactions (SUSAR)

### 9.3.1 Definition of SUSAR

Suspected Unexpected Serious Adverse Reaction (SUSAR) refers to a serious adverse reaction that is suspected to be related to the investigational product and whose nature and severity exceed the information provided in the investigator's brochure, the product labeling of marketed drugs, or the summary of product characteristics.

In this context, "suspected" refers to the correlation with the investigational product, as detailed in the definition of correlation, which outlines the criteria for assessing the relationship between adverse events and the investigational product.The definition of "serious" can be found in the section on "definitions of serious adverse events."

"Unexpected" adverse reactions refer to those whose nature, severity, consequences, or frequency differ from the expected risks described in the current relevant documentation of the investigational product (such as the investigator's brochure). The investigator's brochure serves as the primary document providing safety reference information to determine whether an adverse reaction is expected or unexpected.

### 9.3.2 Reporting of SUSAR

The sponsor is required to assess the expectedness and relatedness of Serious Adverse Events (SAE) received during the clinical trial of the drug. Any SUSAR determined to be related to the investigational product must be reported to the drug regulatory authority and the health administration authority within the specified time frame (7/15 days) in accordance with relevant regulations.The sponsor should also promptly report SUSARs to all investigators and clinical trial sites participating in the study, as well as to the ethics committee.

Investigators are required to report the SUSARs provided by the sponsor to the ethics committee.

## 9.4 Pregnancy

If a female participant or the spouse of a male participant becomes pregnant during the treatment period or within 3 months after the last administration of the investigational product, the investigator must be notified immediately. If the pregnancy occurs during the treatment period, the female participant should discontinue the investigational product immediately. Upon discovering the pregnancy, the investigator should complete the Pregnancy Report Form and report it to the sponsor (or authorized representative) within 24 hours, and also record it in the electronic Case Report Form (eCRF) for follow-up on the outcome. The pregnancy event should be followed up until 30 days after the end of the pregnancy.

Any AE or SAE occurring during the pregnancy, such as spontaneous abortion or induced abortion, birth defects or congenital anomalies in the newborn, stillbirth anomalies and abnormalities, and complications in the mother and newborn, should be reported in accordance with the "Recording and Reporting of Adverse Events" guidelines.

# 10 Data Management

## 10.1 Database Establishment

In this study, data from the clinical research is collected using the eCRF (electronic Case Report Form) remote data entry method. The data manager designs the database and tests it with either simulated data or real eCRF data to ensure the accuracy of the database.

## 10.2 Database Verification

During the data management process, the methods for data verification include data logic checks, manual reviews, medical reviews, and statistical pre-analysis checks. Any data queries will be documented in electronic form within the Electronic Data Capture (EDC) system for the research center to address. If the response meets the requirements, the query will be closed. If the data query remains unresolved or if new queries arise after updating the database based on the previous query's response, the researcher or clinical coordinator will need to confirm with the researcher and provide a new response. This process will be repeated until all data in the database is confirmed to be accurate.

## 10.3 Database Locking

The data manager prepares a data review report based on the study protocol, data review standards, and the database. A data review meeting is convened by the project manager, attended by the sponsor, principal investigator, statistician, and data manager, to review the data. Representatives from all parties present will sign the data review resolution. Once all parties approve the locking of the data, the data manager will organize and execute the data locking process. The locked data will then be submitted to the statistician for statistical analysis.

Data can be locked once the following conditions are met:

1) All data has been collected and entered into the database.

2) All coding has been verified and confirmed.

3) All data queries have been resolved (including those raised during data review).

4) Data review has been completed.

5) Verification of original documents has been completed.

6) The review of Serious Adverse Events (SAEs) has been completed.

7) Signatures from all investigator have been obtained.

8) Analyzable cases have been defined and saved in the final analysis database.

# 11 Statistical Analysis and Sample Size

## 11.1 Sample Size Calculation

This study does not conduct formal statistical hypothesis testing. A total of 240 subjects were randomly assigned to the experimental drug group or the placebo group in a 3:1 ratio. The specific grouping and number of subjects in the study are as follows: 80 mg dose group (N=60) 80 mg dose-matched placebo group (N=20) 120 mg dose group (N=60) 120 mg dose-matched placebo group (N=20) 160 mg dose fast titration group (N=30) 160 mg dose fast titration matched placebo group (N=10) 160 mg dose slow titration group (N=30) 160 mg dose slow titration matched placebo group (N=10) In total, 180 subjects received treatment with VCT220 tablets, while 60 subjects received placebo treatment.

## 11.2 Statistical Analysis Data Sets

Full Analysis Set (FAS)：Defined as all randomized subjects who have received the study drug at least once. The FAS population will serve as the primary analysis population for the efficacy analysis of this study.

Safety Analysis Set (SS)：Defined as all subjects who have received the study drug at least once. The safety analysis population is the primary analysis population for safety endpoints and will be analyzed based on the actual treatment received.

Pharmacokinetic Concentration Set (PKCS)：Defined as all randomized subjects who have received the study drug at least once and have at least one evaluable concentration data point after dosing.

Pharmacokinetic Parameter Set (PKPS)：Defined as all randomized subjects who have received the study drug at least once and have at least one evaluable pharmacokinetic parameter after dosing.

## 11.3 Statistical Analysis Methods

### 11.3.1 General Principles of Statistical Analysis

This study will use SAS version 9.4 or higher as the statistical analysis software, and the entire analysis process will be programmatic. Quantitative variables will be summarized using descriptive statistics, including counts, means, standard deviations, medians, minimums, and maximums. Qualitative or ordinal variables will be summarized using descriptive statistics, including the number of subjects, percentages, and/or event counts.

Typically, any changes compared to baseline, baseline values used in listings/summaries/graphics, or baseline values used as covariates in statistical analyses refer to the last available value prior to randomization. If necessary, specific parameters or assessments will be defined in detail in the Statistical Analysis Plan (SAP).

### 11.3.2 Demographic and Baseline Characteristics Analysis

Baseline data of subjects will be summarized by treatment group, including demographics, medical history, diagnosis and treatment history, and prior treatments, using descriptive statistics. The medical coding for prior treatments and concomitant medications will utilize the WHODrug dictionary. Continuous variables will be statistically described using counts (including the number of missing cases), means, standard deviations, medians, minimums, and maximums. Categorical variables will be statistically described using the number of subjects and percentages.

### 11.3.3 Efficacy Analysis

Based on the FAS, the analysis of the primary efficacy endpoints of this study will employ Analysis of Covariance (ANCOVA) as the primary analytical method. The model will treat treatment as a factor and baseline weight as a covariate, examining the different residual variances across the treatment groups.The primary analysis results will include point estimates of the inter-group differences (treatment group - control group) in the change values relative to baseline, along with the corresponding 95% confidence intervals.

The analysis of secondary endpoints will be conducted based on the Full Analysis Set (FAS). For continuous variables, an ANCOVA model will be constructed with treatment group as the effect and baseline weight as a covariate. The model will calculate the least squares estimate of the inter-group difference between the treatment group and the placebo group, along with the corresponding 95% confidence interval.For categorical variables, the Newcombe hybrid score method will be used to calculate the rate differences between groups and their 95% confidence intervals.

The efficacy analysis of this study will be summarized according to the 80 mg dose group, 120 mg dose group, and 160 mg dose group (including both the fast titration and slow titration subgroups of the 160 mg dose).

### 11.3.4 Safety Analysis

Based on the Safety Set (SS), all AEs will be grouped and summarized using the latest version of the Medical Dictionary for Regulatory Activities (MedDRA) or the updated Chinese version, categorized by System Organ Class (SOC) and Preferred Term (PT). If a single subject experiences multiple AEs, the incidence will be counted as one case. For subjects who experience the same event multiple times, only the highest severity will be counted as one occurrence of that event.

Summarize the occurrence counts, cases, and percentages of Treatment-Emergent Adverse Events (TEAEs) according to System Organ Class (SOC) and Preferred Term (PT), and categorize TEAEs based on their relationship to the study drug and the Common Terminology Criteria for Adverse Events (CTCAE) grade. Separate summaries will be provided for TEAEs related to the study drug, Serious Adverse Events (SAEs), TEAEs leading to treatment discontinuation, and TEAEs leading to death. A detailed listing will describe all AEs, including the following information: （AE Name Start Date End Date Outcome Severity Relationship to Study Drug Treatment Measures Taken for AE Actions Taken Regarding the Study Drug Due to AE.）

Based on the Safety Set (SS), a cross-tabulation will be created for physical examinations, 12-lead ECGs, and laboratory test results compared to baseline measurements, recording the counts and percentages for each. For vital signs (blood pressure, pulse, temperature), statistical descriptions will be provided, including the values at each time point and the changes relative to baseline.

The safety analysis of this study will be summarized according to the 80 mg dose group, 120 mg dose group, 160 mg fast titration group, and 160 mg slow titration group.

### 11.3.5 Pharmacokinetic Analysis

Based on the PKCS, descriptive summaries of concentration data will be provided according to PK sampling times and doses. Individual subject blood concentration-time curves will be plotted by dose using linear and semi-logarithmic coordinates, along with the corresponding summary curves (mean ± standard deviation curves). When calculating summary statistics and plotting summary curves (mean) according to sampling times, the planned PK sampling times will be used; when plotting individual subject curves by time, the actual PK sampling times will be used.

For each dose group, descriptive summaries of the PK parameters will be provided: Cmax, Cmin, Tmax, AUC0-last, and AUC0-tau. If the data permit, additional parameters such as AUC_0-inf_, t_1/2_, CL/F, and Vz/F will also be included.

If the data allow, a power function model and dose normalization method will be used to explore the linear relationship between the primary exposure parameters (Cmax and AUC_0-tau_) and the dose.

### 11.3.6 Population Pharmacokinetic Analysis and Exposure-Response Analysis

All blood concentration and related data obtained from this study will be combined with existing clinical study data of VCT220 tablets to establish a PopPK model (using a nonlinear mixed-effects model [NONMEM]) to characterize the pharmacokinetic (PK) features of VCT220. Furthermore, exposure-response analyses will be conducted, including exploratory analyses of the correlation between exposure-efficacy and exposure-safety. The results of these analyses will be reported separately. Detailed methods and content regarding the PopPK model and exposure-response model analyses will be elaborated in the quantitative pharmacology analysis plan.

# 12 Research Management

## 12.1 Ethics

### 12.1.1 Ethical Requirements

The implementation of this study will strictly adhere to the "Good Clinical Practice" (GCP), the Declaration of Helsinki, relevant regulations, and the review opinions of the ethics committee.

The researcher must ensure that this study is reviewed and approved by a qualified ethics committee that meets GCP requirements. Before the study begins, the researcher should submit the study protocol, informed consent form, and any other necessary materials to the ethics committee for review and approval. The sponsor can only provide the investigational drug after receiving the approval from the ethics committee. Additionally, the ethics committee must be informed of any subsequent amendments to the study protocol that may affect the safety of the subjects and their continued participation in the study, as well as any SAEs that occur during the study. The researcher is responsible for reporting the progress of the study to the ethics committee. Furthermore, the researcher must promptly provide copies of all communications with the ethics committee to the sponsor. When reviewing and approving the study protocol, the ethics committee must confirm the protocol title, protocol number, and indicate the reviewed protocol documents and review date. During the course of the study, if there are any new revisions to the study protocol, informed consent form, etc., written approval from the relevant regulatory authorities must be obtained again in accordance with regulations.

### 12.1.2 Informed Consent

The informed consent form (along with the study protocol) must be reviewed and approved by the ethics committee.

The researcher must provide information about the study both orally and in writing. The researcher or their designated personnel are responsible for explaining the contents of the informed consent form to the subjects (or their guardians or witnesses) in a manner and language that the subjects can understand, making it easy for them to comprehend. When the researcher obtains new information that may affect the subjects' decision to continue participating in the study, they must promptly inform the subjects (or their guardians) and make the appropriate records.

The final text of the informed consent form and other materials provided to the subjects should include the following content: an overview of the study, the purpose of the study, the process and duration of the study, the steps that subjects need to follow in the study, the obligations of the subjects, the expected potential benefits to the subjects (including the possibility of no benefit) and risks involved, information that subjects may be assigned to different groups in the study and the likelihood of being assigned to each group; treatment and corresponding compensation available to subjects in the event of study-related harm; compensation that subjects may receive for participating in the clinical study; confirmation that participation in the study is voluntary; and principles of confidentiality regarding the subjects' personal information, etc.

Before signing the informed consent, the researcher or their designated personnel should provide the subjects (or their guardians) with sufficient time and opportunity to understand the details of the clinical research. The subjects (or their guardians) and the researcher obtaining the informed consent should each sign the informed consent form and indicate the date (if the subject is not signing personally, the relationship should be noted). The informed consent form should be retained in one copy by both the researcher and the subject. If important new information related to the study drug is discovered, the informed consent form must be modified in writing and approved by the ethics committee before obtaining the subjects' (or their guardians') consent again.

### 12.1.3 Confidentiality of Subjects

Investigator have the responsibility to maintain the anonymity of the subjects. Subjects can only be identified in case report forms or other documents using uppercase letters, numbers, and/or codes, and not by their names. investigator must securely store records such as subject enrollment forms and subject identification code lists that contain information linking subject codes to their names and home addresses. investigator must strictly keep confidential any documents that can reveal the identity of the subjects.

## 12.2 Responsibilities of All Parties

### 12.2.1 Responsibilities of investigator

The responsibilities of investigator include, but are not limited to, the following:

1） investigator should collaborate with the sponsor to agree on the study protocol and sign it, implementing it after approval from the ethics committee.

2） investigator must thoroughly read and understand the content of the study protocol and strictly adhere to it during execution.

3）investigator should be familiar with the nature, effects, efficacy, and safety of the study drug (including relevant information from preclinical studies) and should also be aware of all new information related to the drug discovered during the clinical study.

4）Clinical research must be conducted in medical institutions with good medical facilities, laboratory equipment, and adequate staffing, which should be equipped to handle emergencies to ensure the safety of subjects. Laboratory test results must be accurate and reliable.

5）investigator should obtain consent from their medical institution or supervising authority, ensuring they have sufficient time to responsibly complete the clinical study within the specified timeframe. investigator must ensure that all study personnel are well-informed about the study protocol and the study drug, clearly defining their roles and responsibilities in the study, supervising all personnel in executing the study protocol, and implementing quality management measures to ensure an adequate number of eligible subjects are enrolled in the clinical study.

6)Before the implementation of the clinical study, investigator should explain the details of the study approved by the ethics committee to the subjects and obtain written consent.

7)investigator or their authorized clinical doctors are responsible for making medical decisions related to the clinical study, such as ensuring that subjects receive appropriate treatment in the event of adverse events during the study. investigator, as clinical doctors or authorized clinical doctors, must assume all medical decision-making responsibilities related to the clinical study.

8)investigator are obligated to take necessary measures to ensure the safety of subjects and document these measures. For any adverse events related to the study drug, investigator must ensure that subjects receive proper medical treatment and inform them of the relevant circumstances truthfully. In the event of a Serious Adverse Event (SAE) during the clinical study, investigator must immediately take appropriate treatment measures for the subjects and report to the drug regulatory authority, health administration, sponsor, and ethics committee, signing and dating the report.

9）investigator should submit annual reports of the clinical study to the ethics committee or provide progress reports as required by the ethics committee. If any situation arises that may significantly affect the implementation of the study or increase the risk to subjects, investigator should promptly report it in writing to the sponsor, ethics committee, and clinical trial institution.

10）investigator must ensure that data is recorded in the medical records and case report forms in a truthful, accurate, complete, timely, and legal manner.

11） investigator should accept monitoring and auditing by monitors or auditors dispatched by the sponsor, as well as inspections by drug regulatory authorities, to ensure the quality of the clinical study.

12）investigator should agree with the sponsor on the costs related to the clinical study and specify them in the contract. investigator must not charge subjects for the costs associated with the study drug during the clinical study.

13）After the completion of the clinical study, investigator should write a summary report, sign it, date it, and submit it to the sponsor.

### 12.2.2 Responsibilities of the Sponsor

The responsibilities of the sponsor include, but are not limited to, the following:

1） The sponsor is responsible for initiating and applying for the clinical study and providing the research funding.

2）The sponsor provides the investigator's brochure, which includes information and data on the chemical, pharmaceutical, toxicological, pharmacological, and clinical aspects of the study drug (including previous and ongoing studies).

3）The sponsor and the investigator jointly design the clinical study protocol and sign the mutually agreed-upon study protocol and contract.

4）The sponsor provides the investigator with study drugs and control drugs that are easily identifiable, correctly coded, and labeled with special tags, ensuring their quality. The study drugs should be appropriately packaged and stored according to the needs of the study protocol. The sponsor should establish a management system and record-keeping system for the study drugs.

5）The sponsor appoints qualified monitors, who must be accepted by the investigator.

6）The sponsor should establish a quality management system for the clinical study, fulfilling management responsibilities that include the design, implementation, recording, evaluation, reporting of results, and archiving of documents related to the clinical study. The sponsor may organize audits of the clinical study to ensure quality.

7）The sponsor should conduct quality management based on risk, identifying risks that affect key aspects and data of the clinical study. The sponsor should promptly investigate any serious adverse events that occur with the investigator, take necessary measures to ensure the safety and rights of subjects, and report to the drug regulatory authority and health administration in a timely manner.

8）The sponsor is responsible for submitting the summary report of the study to the National Medical Products Administration (NMPA).

9）The sponsor should purchase clinical trial liability insurance for the study. The sponsor should provide comprehensive medical coverage for subjects participating in the clinical study and bear the costs of treatment and corresponding economic compensation for subjects who suffer damage or death related to the study. The sponsor should provide legal and financial guarantees to the investigator, except in cases caused by medical accidents.

## 12.3 Protocol Amendments

Any necessary changes to the protocol must be made in the form of a protocol amendment. These amendments must be submitted for approval or filing with the ethics committee after obtaining the signatures and consent of the sponsor and the principal investigator.

## 12.4 Training

According to GCP principles, clinical monitors must possess qualifications recognized by the sponsor. Before the commencement of the clinical study, the head of the research center should provide training to the investigators on the study protocol. Investigators must thoroughly read and understand the content of the clinical study protocol, master the GCP principles, standardize recording methods and judgment criteria, and strictly adhere to the protocol during execution.

## 12.5 Standard Operating Procedures

The sponsor shall comply with the current GCP guidelines and the existing "Regulations on Drug Registration." The study drugs must meet the relevant requirements of Good Manufacturing Practice (GMP) and undergo strict quality inspections.Laboratory tests shall be conducted by the research center in accordance with Standard Operating Procedures (SOPs).

The methods and quality control for laboratory testing at the research center should be standardized. The clinical laboratory of the research center must perform internal quality control as required and obtain a quality evaluation certificate from the Ministry of Health's clinical testing center.

## 12.6 Quality Control and Assurance

1）Standard Operating Procedures (SOPs): All research processes must establish SOPs.

2）Research Center Qualifications: The clinical trial research center is a drug clinical trial base designated by the National Medical Products Administration (NMPA) that meets clinical conditions.

3）Qualifications of Trial Personnel: Trial personnel must undergo clinical trial training and work under the guidance of senior professionals. Both investigators and other trial personnel must receive training in GCP and the study protocol.

4） Laboratory Quality Control Measures: Laboratories must establish SOPs and quality control procedures for experimental observation indicators.

5）Monitoring: Monitors must receive training in GCP and the study protocol and possess the necessary knowledge for clinical research monitoring, including medicine and pharmacy. Monitors will conduct regular monitoring throughout the research process. They must ensure that the clinical facilities of the research center meet requirements, that investigator adhere to the study protocol, and that research results are accurately recorded. During each monitoring visit, monitors must also verify the electronic Case Report Form (eCRF) against original documents and check the storage conditions and usage records of the study drugs.

6） Auditing: In accordance with GCP requirements, the research center must also undergo audits conducted by auditors appointed by the sponsor. Auditors will systematically review clinical research-related activities and documents to evaluate whether the research is conducted according to the study protocol, SOPs, and relevant regulatory requirements, and whether research data is recorded in a timely, truthful, accurate, and complete manner. Audits should be performed by individuals who are not directly involved in the clinical research.

### 12.6.1 Monitoring

A monitoring plan should be developed for clinical research, and a Standard Operating Procedure (SOP) for monitoring should be established. The monitoring plan must comply with relevant laws and regulations, including monitoring strategies, responsibilities for monitoring all parties involved in the research, methods of monitoring, and the reasons for using different monitoring methods. Emphasis should be placed on protecting the rights of subjects, ensuring data authenticity, and addressing various risks in clinical research.

The specific content of monitoring includes:

1）Pre-Study Confirmation: Confirm that the research institution has appropriate conditions, including staffing and training, that laboratory equipment is complete and functioning well, that all necessary research-related inspection conditions are met, that there is an estimated sufficient number of subjects, and that participating investigator are familiar with the requirements of the study protocol.

2）Drug Management Verification: Verify that the study drugs are supplied, stored, dispensed, and retrieved in accordance with relevant regulations, and that appropriate records are maintained.

3）Protocol Compliance Monitoring: Monitor the investigator'compliance to the study protocol during the research process, verify that investigator fulfill the responsibilities outlined in the study protocol and contract, and ensure that these responsibilities are not delegated to unauthorized personnel. Ensure that investigator receive the latest version of the investigator's brochure, all study-related documents, and necessary study supplies, and that they implement these in accordance with relevant laws and regulations.

4）Subject Eligibility Confirmation: Confirm that enrolled subjects meet eligibility criteria and report on enrollment rates and the progress of the clinical research.

5）Case Report Form (CRF) Accuracy: Confirm the accuracy and completeness of data entered in the CRF and ensure consistency with source documents. Record any follow-up failures, unimplemented studies, or unperformed tests, and whether corrections for errors or omissions have been made in the CRF. Verify that withdrawals and losses to follow-up of enrolled subjects are documented in the CRF with explanations. Ensure that any corrections, additions, or deletions are made by the investigator or authorized personnel, with signatures, dates, and, if necessary, reasons for the modifications.

6）Adverse Event Reporting: Confirm that all Adverse Events (AEs) are reported in accordance with relevant laws and regulations, the study protocol, ethics committee requirements, and sponsor requirements, within the specified timeframes.

7）Protocol Deviations Communication: Communicate promptly with investigator regarding any deviations from the study protocol, SOPs, or relevant legal requirements, and take appropriate measures to prevent recurrence.

8）Assistance with Notifications and Applications: Assist investigator with necessary notifications and applications, and report research data and results to the sponsor.

9）Written Monitoring Reports: Complete a written monitoring report after each monitoring visit, detailing the date, time, monitor's name, and names of investigator and other personnel contacted. The sponsor should review and follow up on issues identified in the monitoring report and maintain documentation of these actions.

### 12.6.2 Auditing and Inspection

The sponsor shall establish auditing procedures for clinical research and the research quality management system to ensure the implementation of these auditing procedures during clinical trials. The sponsor or designated auditors will conduct audits of clinical trial sites, including systematic and independent checks of clinical research-related activities and documents.

After reasonable notice, investigator must allow auditors appointed by the sponsor to conduct research-related audits and inspections by regulatory authorities. The primary purpose of the audit or inspection is to assess whether the implementation of clinical research-related activities, as well as the recording, analysis, and reporting of research data, complies with the study protocol, SOPs, and relevant laws and regulations. investigator must ensure that auditors have direct access to all trial documents, original records, and raw data. Any issues observed and discovered by auditors during the auditing process must be documented in writing.

The drug regulatory authority may request the sponsor to provide audit reports as needed.

## 12.7 Early Termination or Suspension of the Study

Investigator's Request

Investigators may find themselves unable to continue the study due to certain circumstances. According to GCP requirements, if an investigator terminates or suspends the clinical research without prior discussion with the sponsor, they must immediately report this to the clinical trial institution, the sponsor, and the ethics committee, providing a detailed written explanation.

**Sponsor's Request**

The sponsor may request the early termination of the study or the early termination of the study at a specific research center for the following reasons:

1） Funding Issues: Insufficient funding to continue the study.

2）Management Issues: Problems related to the management of the study.

3）Non-compliance by Investigators: Investigators are unable to adhere to the study protocol, GCP, etc.

4） Insufficient Subject Recruitment: Investigators are unable to recruit enough subjects.

5） Safety Considerations: Concerns regarding the safety of participants.

According to GCP requirements, if the sponsor terminates or suspends the clinical research, they must notify the investigators and the drug regulatory authority. The investigators are then required to report this to the clinical trial institution and the ethics committee, providing a detailed written explanation.

**Ethics Committee**

If the ethics committee terminates or suspends an already approved clinical study, the investigator must immediately report this to the clinical trial institution and the sponsor, providing a detailed written explanation.

**Government Regulatory Authority Requirements**

According to the "Drug Registration Management Measures," the National Medical Products Administration (NMPA) may order the sponsor to modify the clinical research protocol, suspend, or prematurely terminate the study under the following circumstances:

1）Failure of the Ethics Committee: The ethics committee has not fulfilled its responsibilities.
2）Inadequate Safety Assurance: The study cannot effectively ensure the safety of the subjects.
3）Failure to Submit Safety Updates: The sponsor has not submitted safety update reports as required during the research period.
4）Delayed Handling of SUSARs: The sponsor has not timely addressed and reported Serious Unexpected Suspected Adverse Reactions (SUSARs).
5）Evidence of Inefficacy: There is evidence proving that the clinical research drug is ineffective.
6）Quality Issues with the Drug: There are quality problems with the clinical research drug.
7）Fraudulent Activities: There are instances of fraud in the clinical research.
8）Other GCP Violations: Other violations of Good Clinical Practice (GCP) regulations.

## 12.8 Record Keeping and Retention of Study Data

According to GCP requirements, for clinical studies used to apply for drug registration, essential documents must be retained for at least 5 years after the research drug has been approved for marketing. For clinical studies not used for drug registration, essential documents must be retained for at least 5 years after the termination of the clinical study.

## 12.9 Confidentiality and Publication of Research Results

All information related to this study (including but not limited to the following documents: protocol, investigator's brochure) must be kept strictly confidential. Investigators must recognize that the scientific or medical information derived from this study may have commercial value for the sponsor. Investigators should maintain confidentiality regarding information and data related to this study. If there is a desire to publicly publish information related to the study or conclusions drawn from the research, prior consultation with the sponsor is required, along with obtaining the sponsor's written consent. To protect their own interests, the sponsor may require investigators not to publish any information related to the study product until it has received marketing approval.

The sponsor has the right to publish or disseminate information or data related to this study, or to submit it to regulatory authorities. If the sponsor wishes to include the investigator's name in any publication, advertisement, or related content, prior consent from the investigator must be obtained.

# 13 References

[1] Y. Wang, L. Zhao, L. Gao, et al. Health policy and public health implications of obesity in China [J/OL]. Lancet Diabetes & Endocrinol, 2021, 9(7): 446-461.
[2] Report on Nutrition and Chronic Disease Status of Chinese Residents (2020) [J/OL]. 42(06): 521.
[3] Correction to Lancet Diabetes Endocrinol 2021; 9: 373-92. Lancet Diabetes Endocrinol. 2021;9(7):e2. doi:10.1016/S2213-8587(21)00149-2.
[4] S. Sarma, S. Sockalingam, S. Dash. Obesity as a multisystem disease: Trends in obesity rates and obesity-related complications [J/OL]. Diabetes, Obesity & Metabolism, 2021, 23 Suppl 1: 3-16.
[5] Chinese Medical Association Endocrinology Branch. Expert Consensus on Comprehensive Management of Type 2 Diabetes with Obesity in China [J]. Chinese Journal of Diabetes, 2016, 8(011): 662-666. DOI:10.3760/cma.j.issn.1674-5809.2016.11.006.
[6] Jensen M D, Ryan D H, Apovian C M, et al. 2013 AHA/ACC/TOS guideline for the management of overweight and obesity in adults: a report of the American College of Cardiology/American Heart Association Task Force on Practice Guidelines and The Obesity Society. [J]. Journal of the American College of Cardiology, 2014, 63(25): 2985-3023. DOI:10.1016/j.jacc.2013.11.004.
[7] Chinese Medical Association Endocrinology Branch, Chinese Association of Traditional Chinese Medicine Diabetes Branch, Chinese Medical Doctor Association Surgery Branch Obesity and Diabetes Surgery Committee, et al. Clinical Multidisciplinary Consensus on Obesity (2021 Edition) [J]. Chinese Journal of Digestive Surgery, 2021, 20(11): 16. DOI:10.3760/cma.j.cn115610-20210829-00422.
[8] W. T. Garvey, J. I. Mechanick, E. M. Brett, et al. American Association of Clinical Endocrinologists and American College of Endocrinology Comprehensive Clinical Practice Guidelines for Medical Care of Patients with Obesity [J/OL]. Endocrine Practice, 2016, 22(Suppl 3): 1-203.
[9] China International Exchange and Promotive Association for Medical and Health Care Nutrition and Metabolism Management Branch, Chinese Nutrition Society Clinical Nutrition Branch, Chinese Medical Association Diabetes Branch, et al. Guidelines for Medical Nutrition Therapy for Overweight/Obesity in China (2021) [J/OL]. Chinese Journal of Medical Frontiers (Electronic Edition), 2021, 13(11): 1-55.
[10] Chinese Nutrition Society Obesity Prevention and Control Branch, Chinese Nutrition Society Clinical Nutrition Branch, Chinese Preventive Medicine Association Behavioral Health Branch, et al. Expert Consensus on Obesity Prevention and Control for Chinese Residents [J]. Chinese Journal of Epidemiology, 2022, 43(5): 18. DOI:10.3760/cma.j.cn112338-20220402-00253.
[11] Zhang H, Yi M, Chen R. Research Progress on the Efficacy and Safety of Orlistat [J/OL]. Hainan Medical Journal, 2016, 18: 3.
[12] Guidelines for Primary Care Diagnosis and Treatment of Obesity (2019), Chinese Journal of General Practitioners, 2020, 19(02): 95-101.
[13] Ji Linong, Zou Dajin, Hong Tianpei, et al. Expert Guidance on Clinical Application of GLP-1 Receptor Agonists [J]. Chinese Journal of Diabetes, 2018, 26(5): 353-361. DOI:10.3969/j.issn.1006-6187.2018.05.001.
[14] VCT220 Investigator's Brochure.
[15] Novo Nordisk. SAXENDA® (liraglutide) Injection Label (June 15, 2022) [EB/OL]. <https://www.accessdata.fda.gov/drugsatfda_docs/label/2022/206321s015lbl.pdf>
[16] Novo Nordisk. WEGOVY (semaglutide) Injection Label (December 23, 2022) [EB/OL]. https://www.accessdata.fda.gov/drugsatfdadocs/label/2022/215256s005lbl.pdf
[17] Wharton S, Blevins T, Connery L, et al. Daily Oral GLP-1 Receptor Agonist Orforglipron for Adults with Obesity. N Engl J Med. 2023;389(10):877-888. DOI:10.1056/NEJMoa2302392.
[18] National Medical Products Administration Drug Evaluation Center. Technical Guidelines for Clinical Trials of Weight Control Drugs [EB/OL]. 2021.
[19] National Medical Products Administration Drug Evaluation Center. Technical Guidelines for the Evaluation of Adverse Events Related to Drug Clinical Trials (Draft for Comments) [EB/OL]. 2023.

# 14 Appendix

# Appendix 1: Definition of Comorbidities

According to the inclusion criteria of the protocol, subjects with a BMI ≥ 24 kg/m² and < 28 kg/m² must have at least one comorbidity associated with prediabetes (impaired fasting glucose and/or reduced glucose tolerance), hypertension, dyslipidemia, fatty liver, or obstructive sleep apnea syndrome caused by overweight. The specific definitions for hypertension and dyslipidemia are as follows:

**Hypertension:**

Treatment with a stable dose of antihypertensive medication for ≥ 12 weeks at screening, or

Systolic blood pressure ≥ 140 mmHg or diastolic blood pressure ≥ 90 mmHg at screening.

**Dyslipidemia:**

Treatment with a stable dose of lipid-lowering medication for ≥ 12 weeks at screening, or

Low-density lipoprotein cholesterol (LDL-C) ≥ 3.4 mmol/L, or

Triglycerides (TG) ≥ 1.7 mmol/L, or

Total cholesterol (TC) ≥ 5.2 mmol/L, or

Male high-density lipoprotein cholesterol (HDL-C) < 1.0 mmol/L, or

Female HDL-C < 1.3 mmol/L at screening.

# Appendix 2: Instructions for Measuring Height, Weight, and Waist Circumference

(1) Height Measurement

1) Before measurement, ensure that the subject is not wearing a hat, shoes, or socks, and has their hair down.
2) Use a calibrated stadiometer for measurement.
3) The head should be in a neutral position, with the eyes looking straight ahead.
4) Gently lower the measuring arm to the subject's head until the bottom surface touches the highest point of the skull.
5) Check the subject's posture to ensure it is correct; confirm the posture is correct before taking the reading.
6) Read the exact height measurement, recorded in centimeters (cm), and retain one decimal place.

(2) Weight Measurement

1) Before each measurement, confirm that the subject is fasting and has emptied their bladder, removing shoes, socks, and outer clothing, wearing only light clothing (empty pockets) or underwear.
2) Use a calibrated electronic scale consistently; throughout the study, use the same scale to measure the same subject's weight, ideally at the same time each measurement.
3) Ensure the scale reads zero before weighing.
4) Make sure the scale is placed on a stable, flat surface (do not place it on carpet, sloped surfaces, or rough uneven surfaces).
5) Instruct the subject to step onto the scale, placing their feet on either side. They should stand still with arms at their sides, then record their weight in kilograms (kg), retaining one decimal place.
6) Measure weight twice each time, taking the average of the two measurements as the result.

(3) Waist Circumference Measurement

1) Before each measurement, confirm that the subject is fasting and has emptied their bladder, wearing only light clothing or underwear.
2) The subject should stand naturally, relaxing their shoulders and abdomen, breathing steadily, with arms at their sides and feet together, evenly distributing their weight.
3) The horizontal position for waist circumference measurement is at the midpoint between the anterior superior iliac spine and the lower edge of the rib cage along the midaxillary line.
4) Use a measuring tape to encircle the waist at the specified horizontal position; the tape should be snug against the skin (not over clothing) but not constricting, ideally measured by the same designated person.
5) Do not consciously suck in or push out the abdomen; take the reading at the end of a normal exhalation, recorded in centimeters (cm), retaining one decimal place.
6) Measure waist circumference twice each time, taking the average of the two measurements as the result. If the difference between the two measurements exceeds 1 cm, discard that set of measurements and repeat the two measurements.

# Appendix 3: Patient Health Questionnaire-9 (PHQ-9)

In the past two weeks, how often have you been bothered by any of the following problems? (Please mark "√" next to your choice)

| Item | Not at all | Several days | More than half the days | Nearly every day |
| --- | --- | --- | --- | --- |
| Little interest or pleasure in doing things | 0 | 1 | 2 | 3 |
| Feeling down, depressed, or hopeless | 0 | 1 | 2 | 3 |
| Trouble falling asleep, difficulty staying asleep, or sleeping too much | 0 | 1 | 2 | 3 |
| Feeling tired or having little energy | 0 | 1 | 2 | 3 |
| Poor appetite or overeating | 0 | 1 | 2 | 3 |
| Feeling bad about yourself — or that you are a failure or have let yourself or your family down | 0 | 1 | 2 | 3 |
| Trouble concentrating on things, such as reading the newspaper or watching television | 0 | 1 | 2 | 3 |
| Moving or speaking so slowly that other people could notice, or the opposite — being so fidgety or restless that you have been moving around a lot more than usual | 0 | 1 | 2 | 3 |
| Thoughts that you would be better off dead, or of hurting yourself in some way | 0 | 1 | 2 | 3 |
| Total Score | | | | |

If you find that you have the above issues, how much difficulty do these problems cause you in your work, handling family matters, or interacting with others?

🞎 No difficulty at all

🞎 A little difficulty

🞎 Very difficult

🞎 Extremely difficult

# Appendix 4: Hypoglycemia Diagnosis and Treatment Process

The study drug in this research is GLP-1RA, which has the effect of lowering blood sugar levels. During the treatment process, subjects may experience episodes of hypoglycemia. If a subject experiences hypoglycemia, the following diagnosis and treatment process can be referenced to correct the hypoglycemia.

**Hypoglycemia Diagnosis and Treatment Process**

# Appendix 5: BMI Calculation Formula

BMI Calculation Formula: Weight (kg) / Height （m）^2^

# Appendix 6: QTcF Calculation Formula

QTcF＝QT／RR^0.33^

# Appendix 7: Glomerular Filtration Rate Calculation Formula

The CKD-EPI formula is used to calculate the glomerular filtration rate (GFR):

Serum creatinine (Scr) unit conversion: 1 mg/dL = 88.4 μmol/L

| Gender | Scr Range | EPI-GFR Formula Unit: ml·min⁻¹·(1.73 m²)⁻¹ |
| --- | --- | --- |
| Female | Scr≤0.7mg/dL | EPI-GFR=144×[Scr(mg/dL) / 0.7)]^-0.329^×0.993^age^ |
|  | Scr＞0.7mg/dL | EPI-GFR=144×[Scr(mg/dL) / 0.7)]^-1.209^×0.993^age^ |
| Male | Scr≤0.9mg/dL | EPI-GFR=141×[Scr(mg/dL) / 0.9)]^-0.411^×0.993^age^ |
|  | Scr＞0.9mg/dL | EPI-GFR=141×[Scr(mg/dL) / 0.9)]^-1.209^×0.993^age^ |

# Appendix 8: Dietary and Exercise Guidance

The following standards can be referenced to calculate caloric intake and expenditure based on the subject's BMI, providing individualized dietary recommendations.

The overall principle of lifestyle intervention for weight loss is to achieve a negative energy balance, with diet and exercise being important scientific methods. The choice of different dietary patterns should be individualized based on factors such as the patient's gender, age, BMI, and physical activity level.

1.Nutritional Intervention for Obesity

The fundamental cause of obesity is that energy intake exceeds energy consumption. Therefore, the first step in the nutritional prevention and control of obesity is to manage total energy intake, ensuring that the body receives adequate protein and other nutrients, while maintaining a negative energy balance between intake and consumption for a sustained period. A combined approach of dietary control and physical activity is essential for achieving and consolidating therapeutic effects.

Nutritional intervention is the most basic method for weight loss, but due to differences in region, ethnicity, and lifestyle habits, it is difficult to standardize. The "Scientific Research Report on Dietary Guidelines for Chinese Residents 2021" proposed principles for a healthy diet: nutritional balance, long-term benefits, and improvement of quality of life and health status, which should also be followed by obese patients.

The principles of a healthy diet are:(1)Recommended: Whole grains, vegetables, fruits, soy and its products, dairy and its products, fish, meat, nuts, and water (including tea and coffee). (2)Not Recommended:①Salty, pickled, and smoked foods ②High salt ③High sugar and added sugar foods④High fat and fried foods⑤Red meat⑥Alcohol and sugary beverages⑦Dining out and takeout meals.

2.Exercise Intervention for Obesity

A reasonable exercise intervention (including aerobic exercise, resistance training, and combined aerobic and resistance training) can reduce obesity, improve blood pressure, blood lipids, and insulin resistance, lower the incidence of hypertension, type 2 diabetes, and cancer, decrease overall mortality and cardiovascular disease mortality, enhance muscle mass and bone density, as well as alleviate anxiety and depression, and improve mental health, cognitive health, and sleep.

The process for developing a weight loss exercise prescription includes the following steps: First, determine the cause, degree, and health status of the patient's obesity; second, establish safe and effective exercise intensities. If conditions allow, a graded exercise test can be used to determine the safe exercise intensity; finally, create a scientifically sound and effective weight loss exercise prescription. An exercise prescription includes six basic elements: exercise type, exercise intensity, exercise duration, exercise frequency, exercise precautions, and adjustments to the exercise plan. An example of an exercise prescription is shown in the table below:

Example of a Weight Loss Exercise Prescription

| Patient Information | Male .90 kg . 29.4 kg/m² |
| --- | --- |
| Weight Loss Requirement | Weekly Weight Loss Goal: 0.5 kg |
| Exercise Plan | Total Caloric Expenditure Required: 2800 kcal from exercise + 700 kcal reduction from diet = 3500 kcal |
| Exercise Type | Primary Activities: Aerobic exercises such as swimming, brisk walking, and aerobics, combined with resistance training. |
| Exercise Intensity | Intensity Level: Moderate |
| Exercise Frequency | Schedule: 5 days of aerobic exercise and 2 days of resistance training per week. |
| Exercise Duration | Engage in 5 sessions of aerobic exercise each week, with each session consisting of 40 minutes of swimming or 55 minutes of brisk walking, along with 2 sessions of resistance training using dumbbells or other equipment (each session includes 2 sets of 8-12 repetitions, performed twice a week). A 90 kg adult burns approximately 700 kcal per hour swimming and 500 kcal per hour brisk walking. Therefore, each aerobic session can burn about 440 kcal, totaling 2200 kcal for 5 sessions. Each resistance training session burns about 300 kcal, totaling 600 kcal for 2 sessions. The overall caloric expenditure amounts to 2800 kcal. |
| Exercise Precautions | If you experience discomfort such as chest pain, tightness, or palpitations, you should reduce the intensity of your exercise or stop exercising. It is important to perform warm-up activities before exercising and cool-down activities afterward. Wear appropriate footwear, avoid exercising on hazy days, and ensure to keep warm after exercising. |
| Adjustment of Exercise Plan | After a period of exercise, you can assess the weight loss results and make adjustments as needed. |

Patients with moderate to severe obesity often have comorbidities such as fatty liver, type 2 diabetes, hypertension, and coronary heart disease. For these patients, the first priority during exercise should be to ensure safety (assessing exercise risks, controlling exercise intensity, etc.), followed by effectiveness. The preferred exercise for these obese patients is prolonged moderate-intensity aerobic exercise, while those with lower exercise capacity may opt for low-intensity aerobic activities. Resistance training 2 to 3 times a week can also have significant positive effects. It has been well established that both aerobic and resistance exercises can reduce or reverse fatty liver, improve symptoms of type 2 diabetes, hypertension, and coronary heart disease, and lower the incidence of cardiovascular mortality.

Since there is a dose-dependent relationship between exercise volume and health benefits, as well as the risk of cardiovascular disease mortality, it is recommended to engage in a greater amount of exercise to achieve more health benefits, provided there are no contraindications.

Patients with certain obesity-related comorbidities often need to take medication. It is important to guide these patients to reasonably schedule the timing of their medication and the interval before exercising to avoid events such as exercise-related hypoglycemia, hypotension, and exacerbation of fatty liver. For diabetic patients, hypoglycemia can occur during exercise and may also present as delayed hypoglycemia after exercise. Therefore, it is essential to enhance blood glucose monitoring before, during, and after exercise. If symptoms of hypoglycemia such as dizziness, palpitations, fatigue, trembling, or cold sweats occur, exercise should be stopped immediately, and appropriate measures should be taken promptly.

**Statistical Analysis Plan**

**Evaluation of the Efficacy and Safety of VCT220 in Overweight/Obese Subjects: A Multicenter, Randomized, Double-Blind, Placebo-Controlled Phase II Clinical Study**

| **Study Number：** | **VCT220-II-02** |
| --- | --- |
| **Applicant：** | **Vincentage Pharma Co., Ltd.** |
| **Cooperative**  **Research**  **Organization：** | **R&G PharmaStudies Co., Ltd.** |
| **Version：** | **1.0** |
| **Date：** | **August 21, 2024** |

1 Table of Contents

[Version Update History	4](#_Toc175731587)

[1.	Introduction	8](#_Toc175731588)

[2.	Study Objective	8](#_Toc175731589)

[2.1	Primary Objective	8](#_Toc175731590)

[2.2	Secondary Objectives	8](#_Toc175731591)

[2.3	Exploratory Objectives	8](#_Toc175731592)

[3.	Study Design	8](#_Toc175731593)

[3.1	Overall Design	8](#_Toc175731594)

[3.2	Dosing Regimen	10](#_Toc175731595)

[3.3	Randomization	10](#_Toc175731596)

[3.4	Blinding Method	11](#_Toc175731597)

[3.5	Research Process	11](#_Toc175731598)

[The research process is shown in Table 1.	11](#_Toc175731599)

[4.	Assessment Indicators	20](#_Toc175731600)

[4.1	Efficacy Indicators	20](#_Toc175731601)

[4.1.1	Primary Efficacy Endpoint	20](#_Toc175731602)

[4.1.2	Secondary Efficacy Endpoints	20](#_Toc175731603)

[4.2	Safety Indicators	20](#_Toc175731604)

[4.3	Pharmacokinetic Indicators	23](#_Toc175731605)

[5.	Sample Size Calculation	23](#_Toc175731606)

[6.	Analysis Populations	23](#_Toc175731607)

[7.	Statistical Analysis	24](#_Toc175731608)

[7.1	General Principles	24](#_Toc175731609)

[7.1.1	Handling Missing Data	25](#_Toc175731610)

[7.1.2	Definition of Baseline	27](#_Toc175731611)

[7.1.3	Study Days	28](#_Toc175731612)

[7.1.4	Treatment Groups for Statistical Analysis	28](#_Toc175731613)

[7.2	Subject Distribution	28](#_Toc175731614)

[7.2.1	Subject Distribution	28](#_Toc175731615)

[7.2.2	Protocol Deviations	29](#_Toc175731616)

[7.3	Demographic and Baseline Characteristics	29](#_Toc175731617)

[7.3.1	Demographics	29](#_Toc175731618)

[7.3.2	History of Obesity/Overweight	29](#_Toc175731619)

[7.3.3	Past/Concurrent Medical History	29](#_Toc175731620)

[7.3.4	Surgical History	29](#_Toc175731621)

[7.3.5	Clinical Trial History	30](#_Toc175731622)

[7.3.6	Personal History	30](#_Toc175731623)

[7.3.7	Infection Tests and HBV-DNA Testing	30](#_Toc175731624)

[7.3.8	Patient Health Questionnaire-9 (PHQ-9)	30](#_Toc175731625)

[7.4	Past/Concurrent Medications	30](#_Toc175731626)

[7.5	Past/Concurrent Non-Drug Treatments	30](#_Toc175731627)

[7.6	Efficacy Analysis	31](#_Toc175731628)

[7.6.1	Primary Efficacy Endpoint	31](#_Toc175731629)

[Time from First Dose Date	31](#_Toc175731630)

[Visit	31](#_Toc175731631)

[7.6.2	Secondary Efficacy Variables	33](#_Toc175731632)

[7.7	Safety Analysis	34](#_Toc175731633)

[7.7.1	Drug Exposure and Compliance	34](#_Toc175731634)

[7.7.2	Adverse Events	35](#_Toc175731635)

[7.7.3	Laboratory Tests	37](#_Toc175731636)

[7.7.4	12-Lead Electrocardiogram (ECG)	37](#_Toc175731637)

[7.7.5	Vital Signs	38](#_Toc175731638)

[7.7.6	Physical Examination	38](#_Toc175731639)

[7.7.7	Ultrasound of the Liver, Gallbladder, Pancreas, Spleen, and Both Kidneys	38](#_Toc175731640)

[7.7.8	Other Safety Results	38](#_Toc175731641)

[8.	Changes to the Protocol	39](#_Toc175731642)

[9.	Statistical Analysis Table Template	39](#_Toc175731643)

# Version Update History

| **SAP Version/Date** | **Update** |
| --- | --- |
| 1.0 / August 21, 2024 | Not applicable |

# 2 Introduction

This Statistical Analysis Plan (SAP) is prepared based on the study protocol "A Multi-Center, Randomized, Double-Blind, Placebo-Controlled Phase II Clinical Study to Evaluate the Efficacy and Safety of VCT220 Tablets in Overweight/Obese Subjects" (Protocol Number: VCT220-II-02, Version: 1.0, Version Date: October 23, 2023). It aims to describe the statistical analysis methods for safety and efficacy data of the clinical study.

The preparation of the SAP refers to the "Good Clinical Practice (GCP)" issued by the National Medical Products Administration (NMPA), "Guidelines for Planning and Reporting Data Management and Statistical Analysis of Drug Clinical Trials," and "Biostatistical Guidelines for Drug Clinical Trials," developed jointly by statisticians and sponsors. The SAP will be finalized and approved before the database lock (DBL) after the completion of the Phase II study.

# 3 Study Objective

## 3.1 Primary Objective

Evaluate the weight loss effects of different doses of VCT220 tablets in overweight/obese subjects without diabetes.

## 3.2 Secondary Objectives

Evaluate the safety of different doses of VCT220 tablets in overweight/obese subjects without diabetes.

Assess the pharmacokinetic (PK) characteristics of different doses of VCT220 tablets in overweight/obese subjects without diabetes.

## 3.3 Exploratory Objectives

Conduct a preliminary exploration of the correlation between the exposure to VCT220 tablets and their efficacy and adverse events (AE) in overweight/obese subjects without diabetes.

# 4 Study Design

## 4.1 Overall Design

This study is a multicenter, randomized, double-blind, placebo-controlled phase II clinical trial aimed at evaluating the efficacy, safety, and pharmacokinetic (PK) characteristics of different doses of VCT220 tablets in non-diabetic overweight/obese subjects.

The study consists of three phases: the screening phase (up to 2 weeks), the treatment phase (16 weeks), and the safety follow-up phase (2 weeks). A total of 240 subjects meeting the inclusion criteria were randomly assigned in a ratio of 3:1 to the experimental drug group and the placebo group, receiving either VCT220 tablets or placebo treatment. The specific grouping and number of subjects in the study are as follows:80 mg treatment group (N=60),80 mg dose-matched placebo group (N=20),120 mg treatment group (N=60),120 mg dose-matched placebo group (N=20),160 mg rapid titration treatment group (N=30),160 mg rapid titration matched placebo group (N=10),160 mg slow titration treatment group (N=30),160 mg slow titration matched placebo group (N=10)

Subjects were enrolled in each treatment group in parallel. In total, 180 subjects received VCT220 tablets, while 60 subjects received placebo treatment. The randomly assigned subjects were administered the corresponding doses of VCT220 tablets or placebo once daily, taken orally with a meal at breakfast, for a continuous 16 weeks. After completing the 16-week treatment, all subjects will enter a 2-week safety follow-up period.

The dosing regimen for each treatment group (and its corresponding placebo group) during the treatment phase is shown in Figure 1.

Figure 1: Study Flow Chart

## 4.2 Dosing Regimen

Investigational Drug:

Name: VCT220 Tablets

Specification: Tablets, 10mg/tablet, 50mg/tablet

#### 4.2.1.1 Control Drug:

Name: VCT220 Placebo Tablets

Specification: Tablets

Characteristics of Placebo:

The appearance (shape, color, size, packaging), odor of the placebo is identical to that of the VCT220 tablets, and it does not contain any active ingredients.

#### 4.2.1.2 Dosing Regimen:

In this study, VCT220 tablets or placebo (VCT220 placebo tablets) are administered orally once daily, taken with meals, and it is recommended to take them with breakfast.

The number and dosage of medications taken by each treatment group during the treatment phase are detailed in section 5.2.1 of the protocol.

Dose Adjustment Plan:

If a subject experiences intolerable gastrointestinal adverse reactions during the dose titration phase, and the investigator determines that the current treatment regimen cannot be continued, treatment should be discontinued. Dose adjustments for subjects in the study are not permitted

## 4.3 Randomization

This trial will use a block randomization method, with subjects randomly assigned to the following treatment groups in a ratio of 6:2:6:2:3:1:3:1: 80 mg treatment group (N=60), 80 mg dose-matched placebo group (N=20), 120 mg treatment group (N=60), 120 mg dose-matched placebo group (N=20), 160 mg rapid titration group (N=30), 160 mg dose-matched rapid titration placebo group (N=10), 160 mg slow titration group (N=30), and 160 mg dose-matched slow titration placebo group (N=10).
For any randomized subjects who withdraw from the clinical trial for any reason, regardless of whether they have received the study drug, their randomization number will be retained, and the withdrawn subjects will not be allowed to participate in this trial again.

## 4.4 Blinding Method

This study employs a double-blind design, wherein the study drug is provided by the sponsor or its designated unit, ensuring that the trial medication and control drug are consistent in packaging appearance, odor, and color.
Any documents related to the grouping information of the study drug will be stored separately in a restricted access location until the database is locked. The project statistician will apply for unblinding, which will be completed after approval from the sponsor. The unblinding in this study will occur collectively after the database is locked, revealing the groups corresponding to the randomization numbers for the purpose of statistical analysis of all grouped data. The unblinding documents will be co-signed by the principal investigator, sponsor, and statisticians.

## 4.5 Research Process

This study includes the following phases:Screening Period (up to 2 weeks),Treatment Period (16 weeks),Safety Follow-Up Period (2 weeks)

# The research process is shown in Table 1.

| Project/Study Duration | Screening Period | Treatment Period | | | | | | | Safety Follow-up Period | Early Withdrawal |
| --- | --- | --- | --- | --- | --- | --- | --- | --- | --- | --- |
| Visit^&^ | **V0** | **V1** | **V2** | **V3** | **V4** | **V5** | **V6** | **V7** | **V8** |  |
| Visit Time (Weeks) | **W-2~W-1** | **W1**# | **W3** | **W5** | **W7** | **W9** | **W13** | **W17** | **W18** |  |
| Visit Time (Days) | **D-14~D-1** | **D1** | **D15** | **D29** | **D43** | **D57** | **D85** | **D113** | **D126** |  |
| Time Window (Days) | **-** | **-** | **±3** | **±3** | **±3** | **±3** | **±3** | **+3** | **±7** |  |
| Research Management Process |  |  |  |  |  |  |  |  |  |  |
| Informed Consent | X |  |  |  |  |  |  |  |  |  |
| Collection of Demographic Information^1^ | X |  |  |  |  |  |  |  |  |  |
| Inquiry and Record of Medical History^2^ | X |  |  |  |  |  |  |  |  |  |
| History of Previous Treatments^3^ | X |  |  |  |  |  |  |  |  |  |
| Confirm Inclusion/Exclusion Criteria | X | X |  |  |  |  |  |  |  |  |
| Randomization^4^ |  | X |  |  |  |  |  |  |  |  |
| Safety Clinical Procedures/Evaluation |  |  |  |  |  |  |  |  |  |  |
| Vital Signs^5^ | X | X | X | X | X | X | X | X | X | X |
| Complete Physical Examination^6^ | X | X |  |  |  |  |  | X | X | X |
| Symptom-Oriented Physical Examination^7^ |  |  | X | X | X | X | X |  |  |  |
| 12-Lead Electrocardiogram (ECG)^8^ | X | X | X | X | X | X | X | X | X | X |
| Complete Blood Count (CBC)^9^ | X | X | X | X | X | X | X | X | X | X |
| Blood Biochemistry^10^ | X | X | X | X | X | X | X | X | X | X |
| Urinalysis^11^ | X | X | X | X | X | X | X | X | X | X |
| Serum Amylase and Lipase | X | X | X | X | X | X | X | X | X | X |
| Serum Pregnancy Test^12^ | X | X |  | X |  |  |  | X | X | X |
| Thyroid Function^13^ | X |  |  | X |  |  |  | X | X | X |
| Coagulation Function^14^ | X |  |  | X |  |  |  | X | X | X |
| Calcitonin | X |  |  | X |  |  |  | X | X | X |
| Infectious Disease Panel^15^ | X |  |  |  |  |  |  |  |  |  |
| PHQ-9 | X |  |  |  |  |  |  |  |  |  |
| Ultrasound of Liver, Gallbladder, Pancreas, Spleen, and Kidneys^16^ | X |  |  |  |  |  |  | X | X | X |
| Efficacy Clinical Procedures/Evaluation |  |  |  |  |  |  |  |  |  |  |
| Height Measurement^17^ | X |  |  |  |  |  |  |  |  |  |
| Fasting Weight Measurement^18^ | X | X | X | X | X | X | X | X | X | X |
| Fasting Waist Circumference^19^ | X | X | X | X | X | X | X | X | X | X |
| FPG* | X | X* | X* | X* | X* | X* | X* | X* | X | X* |
| HbA1c* | X | X* |  | X* |  |  |  | X* |  | X* |
| Fasting Insulin* |  | X* |  | X* |  |  |  | X* |  | X* |
| Fasting C-Peptide* | X | X* |  | X* |  |  |  | X* |  | X* |
| Fasting Lipids^20^* | X | X* |  | X* |  |  |  | X* |  | X* |
| Biological Sample Collection |  |  |  |  |  |  |  |  |  |  |
| Sparse Pharmacokinetic Blood Sample Collection^21^* |  | X | X | X | X | X | X |  |  | X |
| Intensive Pharmacokinetic Blood Sample Collection^22^* |  | X | X | X | X | X | X |  |  | X |
| Medication/Diary Card |  |  |  |  |  |  |  |  |  |  |
| Medication Dispensing |  | X | X | X | X | X | X |  |  |  |
| Medication Retrieval |  |  | X | X | X | X | X | X |  | X |
| Diary Card Distribution^23^ |  | X | X | X | X | X | X |  |  |  |
| Diary Card Retrieval^23^ |  |  | X | X | X | X | X | X |  | X |
| Other |  |  |  |  |  |  |  |  |  |  |
| Adverse Events |  | X | X | X | X | X | X | X | X | X |
| Concomitant Treatment | X | X | X | X | X | X | X | X | X | X |
| Dietary and Exercise Guidance^24^ | X | X | X | X | X | X | X | X | X | X |

Notes:

& Subjects should be in a fasting state (at least 8 hours of fasting) during all routine visit assessments.

*FPG, HbA1c, fasting insulin, fasting C-peptide, fasting lipid levels, sparse PK blood samples, and intensive PK blood samples during the treatment period visits and early withdrawal visits will be uniformly tested by the central laboratory. Other unmarked tests, including FPG, HbA1c, fasting C-peptide, and fasting lipid levels from the screening period, as well as all safety assessments conducted during the study (complete blood count, urinalysis, blood biochemistry, blood lipase, blood amylase, blood pregnancy test, thyroid function, coagulation function, calcitonin, and four infection markers), and FPG tests during the safety follow-up period, will be conducted by the local laboratories at each center. If there are tests that cannot be performed by the local laboratory, they may be sent to the central laboratory for testing.

#If the 12-lead ECG, complete blood count, urinalysis, blood biochemistry, blood lipase, and blood amylase tests conducted during the screening period are within 7 days before Day 1 (D1), then these tests do not need to be repeated on D1; the screening results will serve as baseline. Any abnormal results from the screening period must be retested to establish the baseline for this study. However, a blood pregnancy test must still be performed on D1. The results of FPG, HbA1c, fasting insulin, fasting C-peptide, and fasting lipid tests conducted on D1 will be considered as baseline. The first dose will be administered after all D1 tests have been completed.

1. Demographic information includes date of birth, gender, and ethnicity.

2. Medical history collection: including a history of obesity/overweight (including associated prediabetes, hypertension, dyslipidemia, fatty liver, obstructive sleep apnea syndrome caused by overweight), other significant medical history, and personal history.

3. Previous treatment history: including treatment for target indications and treatment for other diseases.

4. Randomization: to be completed before the first dose.

5. Vital signs: include blood pressure (mmHg), temperature (℃), and pulse (beats/min). Subjects should rest for at least 5 minutes before measuring vital signs, which will be taken while the subject is at rest. If there is a conflict between the scheduled measurement of vital signs and blood sample collection, vital signs should be measured first.

6. A complete physical examination includes skin/mucous membranes, lymph nodes, head and neck (including thyroid), chest (heart, lungs), abdomen (liver, gallbladder, spleen, kidneys), musculoskeletal system, and nervous system.

7. During the treatment period, the investigator will conduct a symptom-oriented physical examination based on clinical observations and symptoms.

8. 12-lead ECG: record heart rate, PR interval, RR interval, QRS complex, QT interval, QTcF, and ECG diagnosis results. Subjects should rest for at least 5 minutes before the ECG measurement. If the QTcF exceeds 450 msec or the QRS exceeds 120 msec during the screening period, the ECG should be repeated twice (with at least 1 minute interval on the same day) and the subject's eligibility will be based on the average of the three QTcF or QRS values.

9. Complete blood count: includes white blood cell count (WBC), red blood cell count (RBC), hemoglobin (Hb), platelet count (PLT), hematocrit (HCT), neutrophil count (NEUT#), neutrophil percentage (NEUT%), lymphocyte count (LYMPH#), lymphocyte percentage (LYMPH%), eosinophil count (EO#), eosinophil percentage (EO%), basophil count (BASO#), basophil percentage (BASO%), monocyte count (MONO#), and monocyte percentage (MONO%).

10. Blood biochemistry: to be completed in a fasting state before medication; includes total protein (TP), albumin (ALB), globulin (GLO), total bilirubin (TBIL), direct bilirubin (DBIL), alanine aminotransferase (ALT), aspartate aminotransferase (AST), urea/urea nitrogen (BUN), creatinine (Cr), uric acid (UA), lactate dehydrogenase (LDH), creatine kinase (CK), creatine kinase isoenzyme (CK-MB), alkaline phosphatase (ALP), gamma-glutamyl transferase (GGT), potassium (K), sodium (Na), chloride (Cl), magnesium (Mg), calcium (Ca), and phosphorus (P).

11. Urinalysis: includes urine pH, urine protein (PRO), urine ketones (KET), urine specific gravity (SG), urine blood (BLD), urine glucose (GLU), urine urobilinogen (URO), urine bilirubin (BIL), urine red blood cells, and urine white blood cells.

12. Blood pregnancy test: applicable only to reproductive-age female subjects; women who have been postmenopausal for over a year or who have undergone permanent sterilization (e.g., tubal ligation, hysterectomy, or bilateral salpingectomy) are considered not of childbearing potential. If a female subject reports absence of menstruation or irregular menstrual cycles at any time during the study, a blood pregnancy test should be immediately performed to rule out or confirm pregnancy. If the blood pregnancy test result is positive, the study drug should be discontinued immediately.

13. Thyroid function: includes thyroid-stimulating hormone (TSH), free triiodothyronine (FT3), and free thyroxine (FT4).

14. Coagulation function: includes international normalized ratio (INR), activated partial thromboplastin time (APTT), and prothrombin time (PT).

15. Infection panel: includes hepatitis B virus surface antigen (HBsAg), hepatitis C virus (HCV) antibodies, human immunodeficiency virus (HIV) antibodies, and syphilis antibodies (Tp-Ab); if HBsAg is positive, additionally conduct quantitative hepatitis B virus deoxyribonucleic acid (HBV-DNA) testing.

16. Abdominal ultrasound: check areas include the liver, gallbladder, pancreas, spleen, and bilateral kidneys.

17. Height: measured in centimeters (to one decimal place). Measurement standards are detailed in Appendix 2.

18. Fasting weight: measured in kilograms (to one decimal place). All measurements should be repeated twice. Measurement standards are detailed in Appendix 2.

19. Fasting waist circumference: waist circumference is defined as the midpoint between the anterior superior iliac spine and the lower margin of the rib cage along the midaxillary line. Measured in centimeters (to one decimal place). All measurements should be repeated twice. Measurement standards are detailed in Appendix 2.

20. Fasting lipid profile: includes total cholesterol (TC), triglycerides (TG), high-density lipoprotein cholesterol (HDL-C), and low-density lipoprotein cholesterol (LDL-C).

21. Sparse PK blood sample collection: to be conducted among all subjects, with sampling time points detailed in the “PK Sample Time Table.”

22. Intensive PK blood sample collection: planned for approximately 40 subjects, with sampling time points detailed in the “PK Sample Time Table.”

23. Diary card: issued after the first dose at each follow-up visit, guiding subjects in filling out the diary card, which includes adverse events, concomitant medications, and dosing records; subjects will bring back the previous period’s diary card to the center during the next follow-up, where the investigator will enter corresponding data based on the record in the diary card, assess the subject's medication situation, and provide relevant guidance and suggestions.

24. Dietary and exercise guidance: the investigator will provide individualized dietary suggestions and exercise guidance to subjects according to the center’s regulations, and will reinforce guidance if any unreasonable lifestyle habits (such as binge eating) are encountered.

# 5 Assessment Indicators

## 5.1 Efficacy Indicators

### 5.1.1 Primary Efficacy Endpoint

Percentage change in weight from baseline after 16 weeks of treatment.

### 5.1.2 Secondary Efficacy Endpoints

Percentage change in weight from baseline after 8 and 12 weeks of treatment;

Proportion of subjects with weight reduction ≥5%, ≥10%, ≥15%, and ≥20% from baseline after 8, 12, and 16 weeks of treatment;

Change in weight from baseline after 16 weeks of treatment;

Change in BMI from baseline after 16 weeks of treatment;

Change in waist circumference from baseline after 16 weeks of treatment;

Change in uric acid levels from baseline after 16 weeks of treatment;

Change in lipid profile (TC, TG, LDL-C, HDL-C) from baseline after 16 weeks of treatment;

Change in blood glucose levels (FPG, HbA1c, fasting insulin, fasting C-peptide) from baseline after 16 weeks of treatment.

## 5.2 Safety Indicators

Safety assessment includes adverse events, vital signs, physical examinations, 12-lead electrocardiograms (ECGs), laboratory tests, and other relevant evaluations.

**Adverse Events (AE):**

Refers to all adverse medical events that occur in subjects after receiving the investigational drug. These may manifest as symptoms, signs, diseases, or laboratory abnormalities, but are not necessarily causally related to the investigational drug.

Treatment-Emergent Adverse Events (TEAE): Defined as any adverse event that occurs after the first administration of the study drug, or an event that was present before the first administration but has worsened in severity from the time of the first administration to within 2 weeks after the last administration.

Serious Adverse Events (SAE): Refers to the following adverse medical events that occur in subjects after receiving the investigational drug:

1. Death;
2. 2. Life-threatening events;

3. Permanent or severe disability or loss of function;

4.Hospitalization or prolongation of existing hospitalization;

5.Congenital anomalies or birth defects;

6.Other significant medical events. Even if a significant medical event does not immediately threaten life, cause death, or require hospitalization, it is often considered serious if medical intervention is needed to prevent any of the above outcomes.

Criteria for Assessing the Severity of Adverse Events: Based on the NCI CTCAE v5.0, severity is graded as follows: Grade 1 (Mild), Grade 2 (Moderate), Grade 3 (Severe or medically significant but not immediately life-threatening), Grade 4 (Life-threatening; requires urgent intervention), Grade 5 (Death related to the adverse event).

It is important to distinguish between “severe” adverse events and “serious” adverse events. "Severe" is used to describe the intensity and does not necessarily indicate an SAE. For example, a headache may be severe in intensity but would not be classified as an SAE unless it meets the SAE criteria.

AE Relatedness to Study Drug: Adverse events are categorized into five relationship categories: Related, Probably Related, Possibly Related, Probably Not Related, and Not Related.

**Laboratory Tests**

Laboratory tests include the following assessments:Complete Blood Count (CBC),Urinalysis,Blood Biochemistry,Lipid Panel,Lipase,Amylase,Thyroid Function Tests,Coagulation Function Tests,Calcitonin,Infection Markers (Four Specific Tests)The specific metrics for each test can be found in Table 2.

| Project | Inspection Indicators |
| --- | --- |
| Complete Blood Count (CBC) | White Blood Cell Count (WBC),Red Blood Cell Count (RBC),Hemoglobin (Hb),Platelet Count (PLT),Hematocrit (HCT), Neutrophil Count (NEUT#),Neutrophil Percentage (NEUT%),Lymphocyte Count (LYMPH#),Lymphocyte Percentage (LYMPH%),Eosinophil Count (EO#),Eosinophil Percentage (EO%), Basophil Count (BASO#), Basophil Percentage (BASO%),Monocyte Count (MONO#),Monocyte Percentage (MONO%) |
| Blood Biochemistry | Total Protein (TP),Albumin (ALB),Globulin (GLO),total Bilirubin (TBIL),Direct Bilirubin (DBIL),Alanine Aminotransferase (ALT),Aspartate Aminotransferase (AST),**（Urea）/（BUN）** Urea / Blood Urea Nitrogen (BUN)**（Cr）**  ,Creatinine (Cr),**（UA）** - Uric Acid (UA),**（LDH）** - Lactate Dehydrogenase (LDH),**（CK）** - Creatine Kinase (CK),**（CK-MB）,**Creatine Kinase-MB (CK-MB),**（ALP）,**Alkaline Phosphatase (ALP), Gamma-Glutamyl Transferase (GGT),Potassium (K),Sodium (Na), Chloride (Cl),Magnesium (Mg)  Calcium (Ca),Phosphorus (P). |
| Urinalysis | Urine pH,Urine Protein (PRO),Urine Ketones (KET),Urine Specific Gravity (SG),Urine Blood (BLD),Urine Glucose (GLU),Urine Urobilinogen (URO),Urine Bilirubin (BIL),Urine Red Blood Cells,Urine White Blood Cells. |
| Lipase, Amylase | Lipase,Amylase |
| Thyroid Function | Thyroid-Stimulating Hormone (TSH),Free Thyroxine (FT4),Free Triiodothyronine (FT3) |
| Coagulation Function | International Normalized Ratio (INR),Activated Partial Thromboplastin Time (APTT),Prothrombin Time (PT) |
| Calcitonin | Calcitonin |
| Infection Panel (or Quadruple Infection Test) | Hepatitis B Surface Antigen (HBsAg),Hepatitis C Virus Antibody (Anti-HCV),Human Immunodeficiency Virus Antibody (Anti-HIV),Treponema Pallidum Antibody Test (Tp-Ab),Quantitative Hepatitis B Virus DNA Test (HBV-DNA). |

**Vital Signs**

Systolic Blood Pressure (mmHg), Diastolic Blood Pressure (mmHg),Body Temperature (°C),Pulse Rate (beats per minute - bpm)

**Physical Examination**

A complete physical examination includes skin/mucous membranes, lymph nodes, head and neck (including the thyroid), chest (heart and lungs), abdomen (liver, gallbladder, spleen, kidneys), musculoskeletal system, and nervous system, among others. During treatment, the investigator conducts symptom-oriented physical examinations based on clinical observations and symptoms.

**12-lead Electrocardiogram**
The electrocardiogram parameters include heart rate, PR interval, RR interval, QRS duration, QT interval, and QTcF.

Ultrasound of the Liver, Gallbladder, Pancreas, Spleen, and Bilateral Kidneys
The examination areas include the liver, gallbladder, pancreas, spleen, and bilateral kidneys.

## 5.3  Pharmacokinetic Indicators

The evaluation and analysis plan for pharmacokinetic indicators will be provided in a separate document.。

# 6 Sample Size Calculation

This study does not conduct formal statistical hypothesis testing. A total of 240 subjects were randomly assigned to the trial drug group or the placebo group in a ratio of 3:1. The specific grouping and number of participants are as follows: 80 mg treatment group (N=60), 80 mg dose-matched placebo group (N=20), 120 mg treatment group (N=60), 120 mg dose-matched placebo group (N=20), 160 mg rapid titration group (N=30), 160 mg rapid titration matched placebo group (N=10), 160 mg slow titration group (N=30), and 160 mg slow titration matched placebo group (N=10). In total, 180 subjects received treatment with VCT220 tablets, and 60 subjects received placebo treatment.

# 7 Analysis Populations

Full Analysis Set (FAS): Defined as all subjects who were randomly assigned and received at least one dose of the study drug. The FAS population will serve as the primary analysis population for the efficacy analysis of this study.

Per Protocol Set (PPS): Defined as all subjects who completed the treatment with the study drug according to the protocol, had no significant protocol deviations affecting the efficacy outcomes, and had measurable primary efficacy endpoints. The PPS will be used for efficacy analysis and is a subset of the FAS; it will be discussed and finalized during the data review meeting before database lock.

Safety Analysis Set (SS): Defined as all subjects who received at least one dose of the study drug. The safety analysis population is the primary population for safety metrics and will be analyzed based on the actual treatment group received.

Pharmacokinetic Concentration Set (PKCS): Defined as all subjects who were randomly assigned and received at least one dose of the study drug, with at least one evaluable concentration data point after dosing.

Pharmacokinetic Parameter Set (PKPS): Defined as all subjects who were randomly assigned and received at least one dose of the study drug, with at least one evaluable pharmacokinetic parameter after dosing.

# 8 Statistical Analysis

## 8.1 General Principles

Statistical analyses will be conducted using the statistical software SAS 9.4 (or later versions).
This trial does not involve formal hypothesis testing or multiple testing issues; the primary analyses will focus on descriptive statistical methods. Nominal p-values for inter-group comparisons will be provided as necessary, solely to describe the strength of association between outcomes and treatment groups, and will not be used as the basis for formal statistical inferences.

Unless otherwise specified, continuous variables will be described using counts, means, standard deviations, medians, minimums, and maximums; categorical variables will be described using counts and percentages. Percentages will not be calculated if the count is 0. Unless specified otherwise, the denominator for calculating percentages will be the total number of subjects in the corresponding subgroup of the analysis population. All decimal places will be rounded to a maximum of three decimal places. The specification for the number of decimal places for basic descriptive statistics can be found in Table 2.

Unless otherwise noted, results from unscheduled or repeated tests will not be included in the analysis based on planned visits, but will still be included in analyses not specifically based on visits, such as the worst post-treatment outcome analysis. All test results (including unscheduled and repeated test results) should be listed.

MedDRA and WHODrug coding will generally use the latest dictionary versions, and the specific dictionary version number will reference the Data Management Plan (DMP).

****Table 2: Specification for the Number of Decimal Places for Basic Descriptive Statistics****

| **Name** | **Description** | **Decimal Places (dp)** |
| --- | --- | --- |
| N | Number of Subjects in the Corresponding Analysis Set | Always displayed as0 dp |
| n | 8.1.1.1 For Categorical Variables,Number of Subjects per Category  8.1.1.2 For Continuous Variables,Number of Subjects with Complete Results | Always displayed as0 dp |
| % | Percentage | Displayed as1 dp |
| Mean | Arithmetic Mean | Original Data + 1 dp |
| SD | Standard Deviation | Original Data + 2 dp |
| Median | Median | Derived Data/Parameter + 1 dp |
| Min | Minimum | Same as Original Data |
| Max | Maximum | Same as Original Data |
| 95% CI | 95% Confidence Interval | Derived Data/Parameter + 1 dp |
| P-value | P-value | 3 dp |

### 8.1.2 Handling Missing Data

(1) Laboratory Test Values:
If the laboratory test value is recorded as less than (or equal to) or greater than (or equal to) the detection limit value (e.g., <x, ≤x, >x, ≥x), when summarizing descriptive statistics, treat it as the detection limit value (i.e., =x). However, in the data listing, continue to report according to the CRF (Case Report Form) by listing the actual recorded results as “<x”, “≤x”, “>x”, or “≥x”.

(2 ) Missing Dates for Adverse Events:
For missing dates of adverse events, if a comparison to the first drug administration date is necessary and there are no specific instructions, fill in the missing date and time using the method outlined below before making determinations. In the data listing, still report according to the originally collected data.

Filling in Missing Start Dates for Adverse Events:

- If the year and month are known and the year-month is less than the year-month of the first use of the investigational drug:
  Use the last day of the known month to fill in the missing date.
- If the year and month are known and the year-month is equal to the year-month of the first use of the investigational drug:
  Set the start date of the adverse event (AE) to be equal to the date of the first use of the investigational drug.
- If the year and month are known and the year-month is greater than the year-month of the first use of the investigational drug:
  Use the first day of the known month to fill in the missing date.
- If only the year is known and the year is less than the year of the first use of the investigational drug:
  Use "December 31" to fill in the missing date.
- If only the year is known and the year is equal to the year of the first use of the investigational drug:
  Set the start date of the AE to be equal to the date of the first use of the investigational drug.
- If only the year is known and the year is greater than the year of the first use of the investigational drug:
  Use "January 1" to fill in the missing date.
- If the year, month, and day are all missing:
  Use the date of the first use of the investigational drug as the corresponding start date.
- The missing end dates for adverse events (AEs) will be filled in as follows:
- If the year and month are known, use the last day of the known month to fill in the date.
- If only the year is known, use "December 31" to fill in the date.
- If the year, month, and day are all missing, no filling will be done.
- If the filled AE end date is later than the death date or known last survival date, it will be filled to the subject's death date or known last survival date.
- If the filled start date is after the end date, the end date will be set as the corresponding start date.

(3) For missing date and time of prior/concomitant medication and prior/concomitant non-drug treatment, if a comparison with the date and time of the first medication is required, and there are no specific instructions, fill in the missing date and time according to the following methods before making a judgment. When listing data, it should still be presented according to the original data collected.

- The missing start dates for prior/concomitant medication or prior/concomitant non-drug treatment will be filled in as follows:
- If the year and month are known, use the first day of the known month to fill in the date.
- If only the year is known, use "January 1" to fill in the date.
- If the year, month, and day are all missing, no filling will be done for the start date, and the accompanying treatment will be classified as prior medication/non-drug treatment for statistical purposes.
- The missing end dates for prior/concomitant medication or prior/concomitant non-drug treatment will be filled in as follows:
- If the year and month are known, use the last day of the known month to fill in the date.
- If only the year is known, use "December 31" to fill in the date.
- If the filled end date is after the last visit date, then the last visit date will be used as the corresponding end date.
- If the year, month, and day are all missing, no filling will be done for the end date, and the accompanying treatment will be classified as concomitant medication/non-drug treatment for statistical purposes.

(4) According to the principle of conservatism, if the severity of an adverse event is missing, it will be filled in as "≥ Grade 3" for the purposes of statistical analysis in charts.

(5) According to the principle of conservatism, if the assessment of the relationship between the adverse event and the investigational drug is missing, it will also be regarded as an adverse event related to the investigational drug for the purposes of statistical analysis in charts. Specifically, the classifications of "related," "probably related," "possibly related," and missing relationship to the investigational drug will all be defined as adverse events related to the investigational drug.

(6) Missing safety data will not be filled in.

Note: The purpose of filling in is to assist in judgment during summary table creation. When listing data, any filled dates will still be presented according to the original data collected.

### 8.1.3 Definition of Baseline

Unless otherwise specified, the baseline value is defined as the last available value before treatment administration. The change from baseline is defined as the difference between the post-baseline measurement and the baseline value.

### 8.1.4 Study Days

Study days are used to describe the number of days from the date of an event or assessment relative to the date of the start of the study treatment. The date of the first administration of the study drug is considered Day 1 of the study.

The formula for study days is as follows:

Study Days (days) = Event/Assessment Date - Date of First Study Drug Administration + 1

### 8.1.5 Treatment Groups for Statistical Analysis

In this study, the analysis will be conducted based on the treatment groups assigned to the subjects at the time of randomization (the placebo group will need to be combined). Additionally, the subjects in the 160 mg rapid titration group and the 160 mg slow titration group will be combined (referred to as the 160 mg investigational drug combined group) for statistical analysis in the tables. The results for the 160 mg investigational drug combined group will not be presented separately in charts and listings.

## 8.2 Subject Distribution

### 8.2.1 Subject Distribution

Subject distribution will be summarized by each investigational drug group, the placebo group, and the total group.

The summary will include the number and percentage of subjects screened, randomized, those with screening failures, and the main reasons for screening failure (percentages calculated based on screened subjects). It will also summarize the number and percentage of subjects entering the Full Analysis Set (FAS) and the Safety Set (SS) (percentages calculated based on the number of subjects randomized).

Furthermore, the summary will include the number and percentage of subjects who experienced emergency unblinding, death, completed treatment, prematurely withdrew from treatment, the main reasons for early withdrawal from treatment, completed the study, prematurely withdrew from the study, and the main reasons for early withdrawal (percentages calculated based on the number of subjects randomized).

Detailed information regarding subject screening and reasons for screening failure will be presented in a list format.

Additionally, a list will provide details on the dates and main reasons for completing treatment or early withdrawal from treatment, as well as the dates and main reasons for completing or prematurely withdrawing from the study.

### 8.2.2 Protocol Deviations

Protocol deviations will be summarized by each investigational drug group, the placebo group, and the total group. The summary will include the number and percentage of subjects with major and minor protocol deviations based on the Full Analysis Set (FAS). Additionally, a list of all subjects with protocol deviations will be provided.

## 8.3 Demographic and Baseline Characteristics

Demographic and baseline characteristics analysis will be conducted based on the Full Analysis Set (FAS), summarized by investigational drug group, placebo group, and the total group.

### 8.3.1  Demographics

Statistical descriptions will include the subjects' age (in years), gender, reproductive capability (for females), ethnicity, height (in cm), weight (in kg), Body Mass Index (BMI in kg/m²) and its classification (normal [BMI < 24], overweight [BMI ≥ 24 and < 28], obese [BMI ≥ 28]). Additionally, detailed demographic information for each subject will be listed.

### 8.3.2 History of Obesity/Overweight

Summarize the number and percentage of subjects with a history of obesity/overweight in each treatment group, along with the number and percentage of subjects corresponding to each category of medical history. Detailed information regarding each subject's history of obesity/overweight will be listed.

### 8.3.3 Past/Concurrent Medical History

Summarize medical history using the MedDRA coding system for System Organ Class (SOC) and Preferred Terms (PT), including the number and percentage of subjects. Detailed information regarding each subject's past/concurrent medical history will be listed.

### 8.3.4 Surgical History

Summarize surgical history using the MedDRA coding system for SOC and PT, including the number and percentage of subjects. Detailed information regarding each subject's surgical history will be listed

### 8.3.5 Clinical Trial History

Summarize the number and percentage of subjects who have previously participated in clinical trials. Basic information regarding all subjects' clinical trial history will be listed.

### 8.3.6  Personal History

Summarize the number and percentage of subjects with a history of allergies, and detailed information regarding each subject's allergy history will be listed.

### 8.3.7 Infection Tests and HBV-DNA Testing

Infection tests include Hepatitis B Virus Surface Antigen (HBsAg), Hepatitis C Virus Antibody (HCV-Ab), Human Immunodeficiency Virus Antibody (HIV-Ab), and Treponema Pallidum Antibody (Tp-Ab). If HBsAg is positive, quantitative testing for Hepatitis B Virus Deoxyribonucleic Acid (HBV-DNA) will be conducted.

Summarize the clinical significance assessments of the infection tests during the screening period and the clinical significance assessment of HBV-DNA when HBsAg is positive, including the number and percentage of subjects. Detailed information regarding subjects' infection tests and HBV-DNA quantitative testing will be listed.

### 8.3.8  Patient Health Questionnaire-9 (PHQ-9)

Summarize the number and percentage of subjects who underwent PHQ-9 screening, and provide descriptive statistics of the total scores for each treatment group. Detailed information regarding each subject's PHQ-9 screening will be listed.

## 8.4  Past/Concurrent Medications

Past medications are defined as having an end date prior to the date of the first dose. Concurrent medications are defined as having an end date after the date of the first dose or having a completely missing end date.

Past/concurrent medications will be coded using the WHODrug dictionary, and the number and percentage of subjects will be summarized according to treatment subgroups (ACT 2) and preferred drug names (PN). A list will describe the detailed information regarding each subject's past/concurrent medications.

## 8.5 Past/Concurrent Non-Drug Treatments

Past non-drug treatments are defined as having an end date prior to the date of the first dose. Concurrent non-drug treatments are defined as having an end date after the date of the first dose or having completely missing treatment dates.

Past/concurrent non-drug treatments will be coded using MedDRA, and the number and percentage of subjects will be summarized according to System Organ Class (SOC) and Preferred Terms (PT). A list will describe the detailed information regarding each subject's past/concurrent non-drug treatments.

## 8.6  Efficacy Analysis

The efficacy analysis of this study will be based on the Full Analysis Set (FAS) and Per Protocol Set (PPS), with FAS being the primary analysis set for efficacy. The analysis based on PPS will serve as supportive results and will be conducted according to the treatment regimens assigned to subjects at randomization (the placebo group will need to be combined).

Furthermore, subjects in the 160mg rapid titration group and the 160mg slow titration group will also be combined for the efficacy analysis.

### 8.6.1 Primary Efficacy Endpoint

The primary efficacy endpoint of this study is the percentage change in body weight from baseline after 16 weeks of treatment.

For subjects who have unscheduled visits or premature withdrawal visits, the results of the primary efficacy endpoint will be derived according to the following rules and included in the corresponding scheduled visits for statistical analysis.

| Time from First Dose Date^[1]^ | Visit |
| --- | --- |
| D1 | V1 |
| D15±3 | V2 |
| D29±3 | V3 |
| D43±3 | V4 |
| D57±3 | V5 |
| D85±3 | V6 |
| D113+3 | V7 |

Note 1: Calculation of Time from First Dose Date

**Main Analysis:**

The analysis of the primary efficacy endpoint in this study employs Analysis of Covariance (ANCOVA), using the treatment group as the explanatory variable and baseline body weight as a covariate. It calculates the least squares means and their standard errors, as well as 95% confidence intervals for the percentage change in body weight from baseline after 16 weeks in each treatment group. Additionally, the difference in least squares means between each treatment group and the placebo group is reported, along with the corresponding 95% confidence intervals and nominal P-values for the comparisons between each group and the placebo group.

Adverse Events: For participants who terminated treatment for any reason before the primary endpoint visit, follow-up will continue, and data from these subjects will be included in the analysis. Similarly, if participants used other medications that may affect efficacy before the primary endpoint visit, follow-up will continue, and data from these subjects will also be included in the analysis.

For missing weight data after 16 weeks of treatment, multiple imputation (MI) will be employed based on the Missing At Random (MAR) assumption. The detailed multiple imputation process is as follows:

1. Based on SAS 9.4's Proc MI, multiple imputation will be performed using the Fully Conditional Specification (FCS) method. The imputation will be conducted

separately for each treatment group, and the imputation model will include baseline body weight and weight data from each planned visit. A total of 1,000 imputations will be planned, generating 1,000 datasets.

2. Each imputed dataset will be analyzed using the ANCOVA method.
3. The results from each analysis will be combined using Rubin's formula (via SAS 9.4's Proc MIANALYZE).

**Sensitivity Analysis 1:**

For the missing weight data after 16 weeks of treatment, the Last Observation Carried Forward (LOCF) method will be used for imputation, and the statistical analysis method will be the same as that of the primary analysis.

Sensitivity Analysis 2:

For the missing weight data after 16 weeks of treatment, no imputation will be performed. Instead, a Mixed Model Repeated Measurements (MMRM) approach will be utilized. The baseline weight will be included as a covariate, with treatment groups, planned visits, and the interaction between treatment groups and planned visits being treated as fixed effects for the sensitivity analysis.

Post-Hoc Analysis 1:

Based on the Per Protocol Set (PPS), the statistical analysis method will be consistent with the primary analysis.

Post-Hoc Analysis 2:

For the primary endpoint, any data collected after the use of other medications that may affect efficacy will not be included in the analysis. In the statistical analysis, it will be assumed that participants using these potentially efficacy-affecting medications are similar in baseline characteristics to participants in the same treatment group who did not use such medications. The handling of missing data and statistical analysis will follow the same procedures as the primary analysis.

Additionally, baseline weight values and the percentage change in weight from baseline will be summarized for each planned visit by treatment group. Line graphs will be used to illustrate the temporal changes in weight and percentage change in weight from baseline for each treatment group at different visits.

A summary table will present the specific results of fasting weight measurements for each participant.

### 8.6.2  Secondary Efficacy Variables

All secondary efficacy variables will be descriptively summarized and compared between groups. The secondary efficacy variables include:

Percentage change in weight from baseline at 8 and 12 weeks of treatment.

Proportion of participants with a percentage decrease in weight from baseline of ≥5%, ≥10%, ≥15%, and ≥20% at 8, 12, and 16 weeks of treatment.

Change in weight from baseline after 16 weeks of treatment.

Change in BMI from baseline after 16 weeks of treatment.

Change in waist circumference from baseline after 16 weeks of treatment.

Change in uric acid levels from baseline after 16 weeks of treatment.

Change in lipid profile (TC, TG, LDL-C, HDL-C) from baseline after 16 weeks of treatment.

Change in blood glucose levels (FPG, HbA1c, fasting insulin, fasting C-peptide) from baseline after 16 weeks of treatment.

The statistical analysis of secondary efficacy variables will be based on available observational data without imputation for missing data.

The analysis method for continuous variables will be the same as that for primary efficacy variables, utilizing Analysis of Covariance (ANCOVA). The baseline values of each efficacy indicator will be included as covariates, with treatment groups as explanatory variables.

This method will estimate the least squares means and standard errors for each treatment group’s efficacy indicators and calculate the intergroup least squares estimated differences relative to the placebo group, along with their 95% confidence intervals.

Summarize the data of each indicator and the corresponding changes relative to baseline for each treatment group at each planned visit. Use line charts to display the longitudinal changes of BMI, waist circumference, uric acid, lipid profile (TC, TG, LDL-C, HDL-C), and blood glucose levels (FPG, HbA1c, fasting insulin, fasting C-peptide) for each treatment group at different visits.

For categorical variables, the Clopper-Pearson exact method will be used to calculate point estimates and 95% confidence intervals for the rates in each treatment group. The Newcombe confidence interval method will be employed to calculate the rate differences between groups and their 95% confidence intervals. The comparisons of intergroup differences will be conducted using the Chi-square test or Fisher's exact probability method.

In addition to analyzing the proportion of participants whose weight decreased by ≥5% from baseline after 16 weeks of treatment, statistical analysis will also be conducted on the proportion of participants whose weight decreased by ≥5%, ≥10%, ≥15%, and ≥20% from baseline after 8 weeks of treatment, as well as the proportion of participants whose weight decreased by ≥5%, ≥10%, ≥15%, and ≥20% from baseline after 12 weeks of treatment, and the proportion of participants whose weight decreased by ≥10%, ≥15%, and ≥20% from baseline after 16 weeks of treatment.Additionally, we will explore the percentage change in weight from baseline after 8 and 12 weeks, without imputing missing data. The statistical analysis methods will be the same as those used for the primary efficacy analysis.

Detailed results for each secondary efficacy endpoint will be presented in tabular format for each participant's visit data.

## 8.7 Safety Analysis

The safety analysis of this study will be based on the Safety Set (SS) and will be conducted according to the actual treatment received by the participants.

### 8.7.1  Drug Exposure and Compliance

In the Safety Set (SS) population, the following drug exposure indicators will be summarized by each treatment group and the placebo group:

Duration of Treatment (days) = Last Dose Date - First Dose Date + 1;

Duration of Drug Exposure (days): The actual number of days the participant received the drug during the treatment period, excluding periods where the dosage was 0;

Actual Total Dose Administered (mg): The sum of the drug doses actually administered to the participant during the treatment period;

Planned Total Dose (mg): The sum of all planned drug doses during the scheduled treatment cycles;

Compliance (%) = (Actual Total Dose Administered (mg) / Planned Total Dose (mg)) × 100%;

Compliance Classification: Good compliance (compliance range between 80% and 120%), Poor compliance (compliance < 80% or > 120%).

The number and percentage of participants corresponding to each classification of drug exposure time will be statistically summarized, with the classifications as follows: ≤ 2 weeks (≤ 14 days), 2-4 weeks (15-28 days), 4-6 weeks (29-42 days), 6-8 weeks (43-56 days), 8-12 weeks (57-84 days), 12-16 weeks (85-112 days), and > 16 weeks (> 113 days).

A list will be provided describing each participant's medication records, drug exposure, and compliance status.

### 8.7.2 Adverse Events

All adverse events (AEs) will be coded using the MedDRA coding system. Definitions related to adverse events can be found in Section 4.2 of the SAP, and handling of missing data is addressed in Section 7.1.1 of the SAP.

When calculating the number of cases and incidence rates, if a single participant experiences multiple AEs, it will be recorded as one case. In calculating the number of events, the actual occurrences of AEs reported by the participant will be used. For recurrent events of the same type occurring over a continuous period, the summary tables will consolidate these events by the highest severity and highest relatedness, counting them as a single event for statistical purposes.

Unless otherwise specified, the summary will focus solely on treatment-emergent adverse events (TEAEs). TEAEs will be summarized by treatment group, System Organ Class (SOC), and Preferred Term (PT) to report the incidence, number of cases, and corresponding percentages. Additionally, TEAEs will be summarized according to their relationship with the investigational drug and the CTCAE grade. The following classifications will be summarized separately:

- All TEAEs
- TEAEs related to the investigational drug
- Serious TEAEs
- Serious TEAEs related to the investigational drug
- TEAEs with severity ≥ Grade 3
- TEAEs related to the investigational drug with severity ≥ Grade 3
- TEAEs leading to medication interruption
- TEAEs related to the investigational drug leading to medication interruption
- TEAEs leading to treatment discontinuation
- TEAEs related to the investigational drug leading to treatment discontinuation
- TEAEs leading to study withdrawal
- TEAEs related to the investigational drug leading to study withdrawal
- TEAEs leading to death
- TEAEs related to the investigational drug leading to death

Additionally, the occurrence, number of cases, and percentages of gastrointestinal disorders, nausea, vomiting, diarrhea, and appetite loss, along with their highest CTCAE grades during the treatment period in each group will be summarized. The duration of these adverse events and the number of days from the first occurrence of the event to the first dose will be descriptively analyzed.

Line charts will be used to display the percentage change over time for gastrointestinal disorders, nausea, vomiting, diarrhea, and appetite loss in each treatment group during the treatment period. Bar charts will illustrate the number of occurrences of the different grades of these events across the treatment groups during the treatment period.

Duration of Adverse Event (days) = Adverse Event End Date - Adverse Event Start Date + 1.

Days from First Occurrence of Adverse Event to First Dose (days) = Earliest Adverse Event Date - First Dose Date + 1

A comprehensive list will be provided documenting all adverse events, serious adverse events, AEs related to the investigational drug, AEs with severity ≥ Grade 3, AEs leading to medication interruption, AEs leading to treatment discontinuation, AEs leading to study withdrawal, and AEs leading to death. This list will include all adverse events occurring during the study period, with severity and relatedness presented as per the original results.

### 8.7.3  Laboratory Tests

Descriptive statistics will be utilized to summarize laboratory test results (including blood routine, blood biochemistry (excluding uric acid), blood amylase, blood lipase, thyroid function, coagulation function, and calcitonin) by treatment group, as well as the relative changes from baseline for each planned visit.

A cross-tabulation will be employed to summarize the changes in baseline clinical assessments and the worst clinical assessments during the treatment period for each treatment group. The clinical assessment results will be determined by the investigator, categorized as: Normal / Clinically Insignificant Abnormal / Clinically Significant Abnormal.

Line graphs will illustrate the longitudinal changes in alanine aminotransferase (ALT), aspartate aminotransferase (AST), blood amylase, blood lipase, and calcitonin at different visits for each treatment group.

For each laboratory test item, detailed information on laboratory indicators (including blood routine, blood biochemistry, urine routine, blood amylase, blood lipase, blood pregnancy, thyroid function, coagulation function, and calcitonin) for each participant will be provided, encompassing both planned and unplanned visits.

### 8.7.4 12-Lead Electrocardiogram (ECG)

Descriptive statistics will be used to summarize the 12-lead ECG metrics (heart rate, PR interval, RR interval, QRS duration, QT interval, and QTcF interval) by treatment group, including data from all planned visits and the relative changes from baseline. Line graphs will be created to show the longitudinal changes in heart rate across different visits for each treatment group.

A cross-tabulation will be employed to summarize the changes in baseline clinical assessments and the worst clinical assessments during the treatment period for each treatment group.

The number of participants and corresponding percentages for each treatment group will be summarized based on the following classifications for baseline QTcF and maximum post-baseline QTcF (including data from both planned and unplanned visits):

- QTcF Values：≤450ms、450ms~480ms、480ms~500ms、>500ms
- Increase in QTcF Relative to Baseline After Treatment:：≤30ms、30ms~60ms、60~90ms、>90ms

12-Lead Electrocardiogram (ECG) Detailed Information

### 8.7.5 Vital Signs

Descriptive statistics will be used to summarize vital signs (systolic blood pressure, diastolic blood pressure, temperature, and pulse) by treatment group, including visit data and relative changes from baseline. Line graphs will illustrate the longitudinal changes in systolic and diastolic blood pressure across different visits for each treatment group.

A cross-tabulation will be created to summarize the changes in baseline clinical assessments and the worst clinical assessments during treatment for vital sign metrics (systolic blood pressure, diastolic blood pressure, temperature, and pulse).

The detailed information for the vital sign assessments (including both planned and unplanned visits) is provided in the table below:

### 8.7.6 Physical Examination

A cross-tabulation will be utilized to summarize the changes in baseline clinical assessments for each examination site and the worst clinical assessments observed during the treatment period.

Below are the detailed lists for the complete physical examination and the symptom-oriented physical examination assessments, including both planned and unplanned visits.

### 8.7.7 Ultrasound of the Liver, Gallbladder, Pancreas, Spleen, and Both Kidneys

A cross-tabulation will be used to summarize changes in the baseline clinical evaluation and the worst clinical evaluation during treatment for the ultrasound examinations of the liver, gallbladder, pancreas, spleen, and both kidneys.

A detailed listing of the ultrasound examination results (including planned and unplanned visits) for the liver, gallbladder, pancreas, spleen, and both kidneys will be provided.

### 8.7.8  Other Safety Results

A list will describe the detailed results of other examinations for subjects, including examination items, examination dates, results, units, normal value ranges, clinical significance assessments, and descriptions of abnormal results.

A list will describe the reasons for emergency unblinding of subjects and the dates of unblinding.

A list will describe detailed information regarding subjects' deaths, including dates of death, causes of death, and autopsy results, among others.

# 9 Changes to the Protocol

SAP Version 1.0 (Version Date: August 21, 2024), written in accordance with Study Protocol Version 1.0 (Version Date: October 23, 2023), has three differences compared to Protocol Version 1.0:

1.A supportive analysis set called the Per Protocol Set (PPS) has been added in this SAP as an efficacy endpoint.

Per Protocol Set (PPS): Defined as all subjects in the trial who completed the study drug treatment as specified in the protocol, without significant protocol deviations affecting efficacy results, and for whom the primary efficacy endpoints can be assessed. The PPS is used for efficacy analysis and is a subset of the Full Analysis Set (FAS), discussed and finalized at the data review meeting before database lock.

2.In this SAP, based on the sponsor's medical advice, several secondary efficacy endpoints have been added, including:

- The percentage change in weight from baseline after 8 and 12 weeks of treatment;
- The proportion of subjects with a weight reduction of ≥5%, ≥10%, ≥15%, and ≥20% from baseline after 8 and 12 weeks of treatment;
- The proportion of subjects with a weight reduction of ≥10%, ≥15%, and ≥20% from baseline after 16 weeks of treatment;
- The change in uric acid levels from baseline after 16 weeks of treatment.
- To avoid ambiguity, the term "absolute change value" in the description of secondary efficacy indicators has been modified to "change value.

# 10 Statistical Analysis Table Template

The statistical analysis table template will be provided in a separate document.
